# Natural plant polyphenols contribute to the ecological and healthy swine production

**DOI:** 10.1186/s40104-024-01096-3

**Published:** 2024-11-04

**Authors:** Huadi Mei, Yuanfei Li, Shusong Wu, Jianhua He

**Affiliations:** 1 College of Animal Science and Technology, Hunan Agriculture University, Changsha, 410128 China; 2 Jiangxi Province Key Laboratory of Genetic Improvement of Indigenous Chicken Breeds, Institute of Biotechnology, Nanchang Normal University, Nanchang, Jiangxi 330000 China

**Keywords:** Health, Physiological function, Polyphenols, Production performance, Swine

## Abstract

The absence of trace amounts of natural bioactive compounds with important biological activities in traditional dietary models for global farm animals, coupled with an incomplete theoretical system for animal nutrition, has led to unbalanced and inadequate animal nutrition. This deficiency has adversely impacted animal health and the ecological environment, presenting formidable challenges to the advancement of the swine breeding industry in various countries around the world toward high-quality development. Recently, due to the ban of antibiotics for growth promotion in swine diets, botanical active compounds have been extensively investigated as feed additives. Polyphenols represent a broad group of plant secondary metabolites. They are natural, non-toxic, pollution-free, and highly reproducible compounds that have a wide range of physiological functions, such as antioxidant, anti-inflammatory, immunomodulatory, antiviral, antibacterial, and metabolic activities. Accordingly, polyphenols have been widely studied and used as feed additives in swine production. This review summarizes the structural characteristics, classification, current application situation, general properties of polyphenols, and the latest research advances on their use in swine production. Additionally, the research and application bottlenecks and future development of plant polyphenols in the animal feed industry are reviewed and prospected. This review aims to stimulate the in-depth study of natural plant polyphenols and the research and development of related products in order to promote the green, healthy, and high-quality development of swine production, while also providing ideas for the innovation and development in the theoretical system of animal nutrition.

**Supplementary Information:**

The online version contains supplementary material available at 10.1186/s40104-024-01096-3.

## Introduction

Antibiotics have been extensively used in animal production due to their healing and growth-promoting properties [1]. However, the resulting generation of drug residues and emergence of drug resistance have become a massive threat to public health. As a result, developed countries, like the European Union, Japan, South Korea, the United States, and some developing countries, such as China and Thailand, have successively banned the addition of antibiotics to feed [2]. Therefore, there is an urgent need to develop and use safe and effective natural feed additives as stimulators of production and immunity to ensure sustainable growth of the livestock and poultry industry.

In addition, under the model of modern intensive and large-scale rearing, farm animals are often continuously trapped in a sub-healthy state for extended periods of time, which results in high morbidity and mortality rates, posing a significant challenge to the high-quality and sustainable development of global animal production. Nutrition stands out as a critical factor impacting animal health, playing a key role among various factors that influence the well-being of animals. As a material basis for maintaining various life activities and a crucial guarantee for animal health, adequate and well-balanced nutrition has the potential to enhance both the health and productivity of animals. For a long time, the animal nutrition community has focused on the supply and balance of proteins, amino acids, fats, carbohydrates, minerals, and vitamins. However, the demand for plant-derived bioactive compounds has been overlooked. Plant-derived feed ingredients contain a variety of biologically active compounds and play an important role in regulating redox homeostasis, immune function, metabolic homeostasis, and endocrine function in animals. Unfortunately, the current animal dietary pattern seriously lacks such natural bioactive compounds due to a variety of factors, including modern selective breeding for crop and feed processing.

Polyphenols are a major group of such bioactive phytochemicals found in plants or plant-based foods, like strawberries, mangoes, apples, rhubarb, tea, onions, red wine, etc. [3]. To date, more than 8,000 types of phenolic compounds have been identified in plants [4], and found to have a variety of physiological activities, such as antioxidant, anti-inflammatory, antibacterial, immunomodulatory, and intestinal health-promoting properties [5–8]. Recently, polyphenols have been widely used as feed additives in animal production due to their effectiveness and economic viability [9, 10]. In particular, comprehensive research findings indicate that the inclusion of polyphenols in swine diets has results in significant improvements in production performance, meat quality, immune response, antioxidant capacity, and metabolic function, while effectively reducing their mortality rates and odor emissions. Therefore, the use of polyphenols as feed additives for swine has promising potential for widespread application. Accordingly, in this review, we summarize the structural characteristics, classification, current application situation, general properties of polyphenols, and the recent research advances on their use in swine production. Additionally, we summarize the challenges in the research and application of plant polyphenols in the feed industry, looking forward to future research developments. This review will help stimulate the in-depth study of natural plant polyphenols, and research and development of related products, in order to promote the green, healthy, and high-quality development of swine production, while also providing new insights into the innovation and development of the theoretical framework of swine nutrition.

## Plant polyphenols and their classifications

Polyphenols are defined as compounds with at least two benzene rings and one or more hydroxyl substituents and are mainly divided into four types according to their chemical structures: flavonoids, phenolic acids, lignans, and stilbenes [11]. Polyphenol compounds are widely found in nature, especially in plants. To date, over 8,000 polyphenol compounds with different structures have been identified from fruits, vegetables, and other plant foods [3, 4].

### Flavonoids

**Fig. 1:**
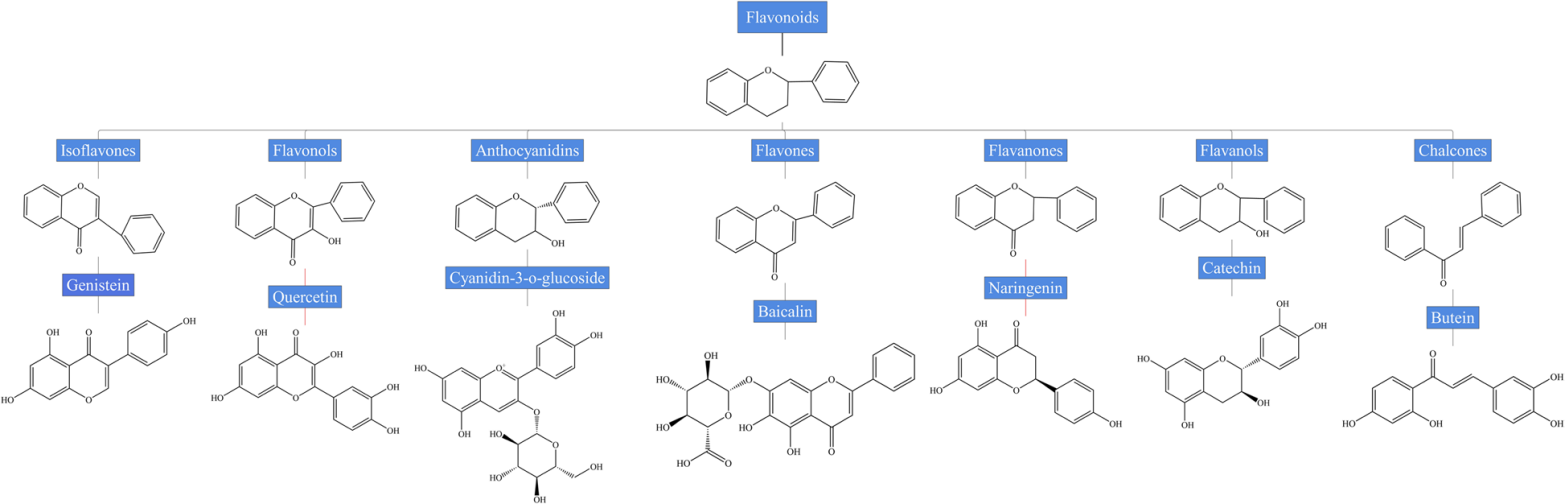
Chemical structures and classification of common flavonoids: flavones, flavanones, flavanols, chalcones, flavonols, anthocyanidins, and isoflavones

Flavonoids are the most abundant natural polyphenols widely found in plants. They have a common basic 15-carbon skeleton structure consisting of two benzene rings (A and B rings) connected by a heterocyclic pyrene ring (C rings) [12]. Flavonoids can be further subdivided into seven main subclasses, namely flavones, flavanones, flavanols, chalcones, flavonols, anthocyanidins, and isoflavones depending on the oxidation state of the heterocycle, substitution content of the C ring, and B ring position (Fig. 1) [11]. To date, over 4,000 types of flavonoids have been identified in plants, including baicalin, quercetin, naringenin, silybin, genistein, cyanidin-3-*O*-glucoside, etc. [13, 14].

### Phenolic acids

**Fig. 2:**
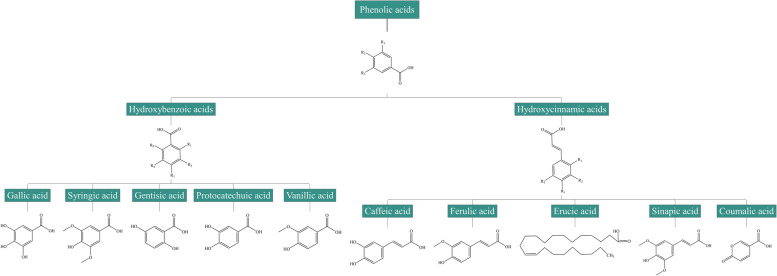
Chemical structures and classification of common phenolic acids: hydroxybenzoic acids and hydroxycinnamic acid

Phenolic acids contain a carboxylic acid group and an aromatic ring with one or more hydroxyl groups connected [15]. In terms of their basic skeleton structure, phenolic acids are classified as hydroxylated derivatives of cinnamic acid or benzoic acid (Fig. 2). Phenolic acids with C_3_-C_6_ backbones are hydroxycinnamic acids, the most abundant phenolic acids in plants, and include erucic acid, ferulic acid, sinapic acid, caffeic acid, coumaric acid, etc. [16]. Phenolic acids with C_1_-C_6_ backbones are hydroxybenzoic acids, and encompass gallic acid, syringic acid, gentisic acid, protocatechuic acid, vanillic acid, etc. [17].

### Lignans and stilbenes

**Fig. 3:**
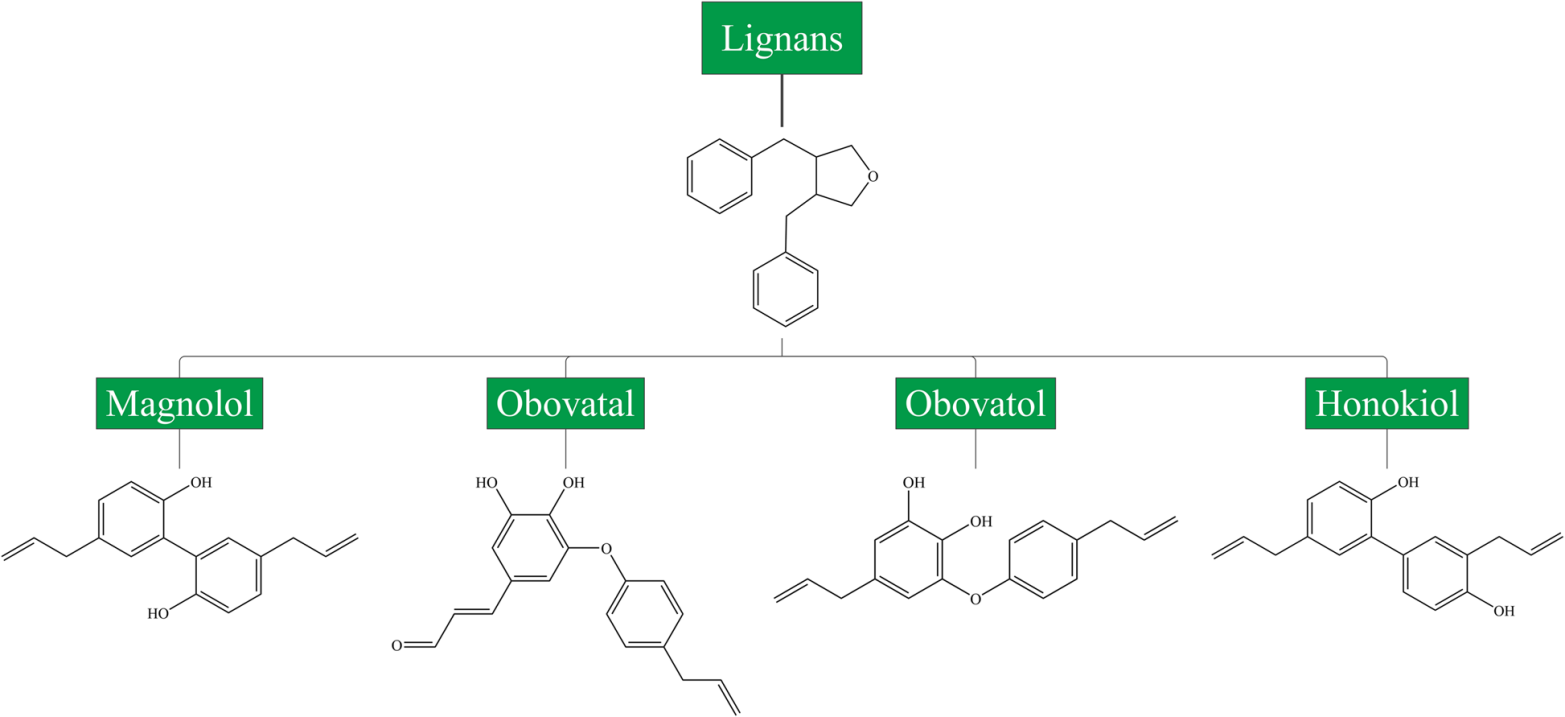
Chemical structures and classification of common lignans

**Fig. 4:**
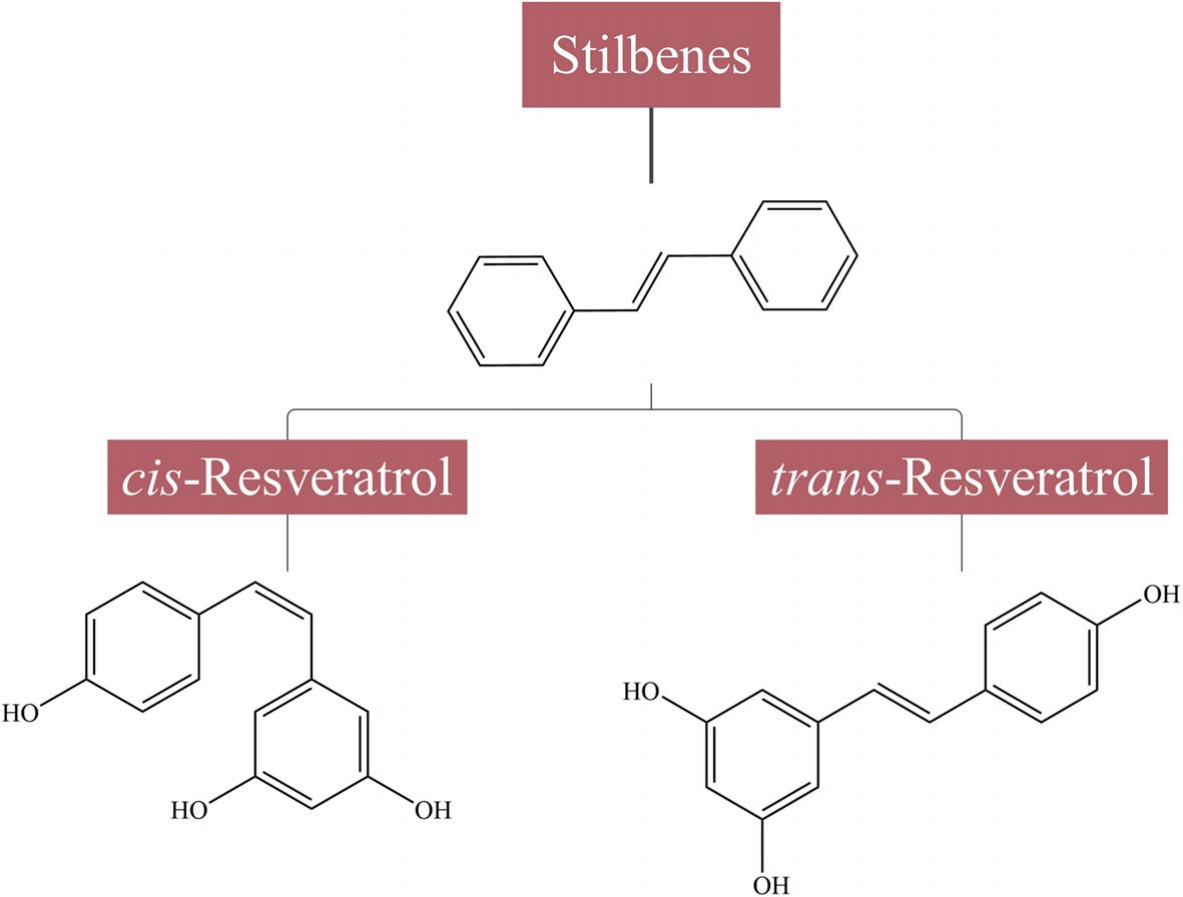
Chemical structures and classification of common stilbenes

Lignans are formed through oxidative dimerization of two or more phenylpropane units by the phenylpropanoids pathway and are characterized by a structure of 2,3-dibenzylbutane [18]. Representative lignans include magnolol, honokiol, obovatol, and obovatal, which are mainly derived from *Magnolia* plants (Fig. 3) [19]. Stilbenes are characterized by a structure of double bonds connecting phenolic rings (Fig. 4) [12]. There are 2 isomeric forms of stilbene, namely *trans*-stilbene and *cis*-stilbene, with the former being the most common form in plants. Resveratrol (3,4′,5-trihydroxy-*trans*-stilbene) is the most common stilbene and has been studied extensively and in-depth [20].

## The current situation of plant polyphenols

There is a long history of understanding and using plant polyphenols by humans. Plant polyphenols have been widely used in the leather industry since ancient times, and now they are mainly used in food, agriculture, medicine, and other fields [21]. The global market for plant polyphenols is concentrated mainly in the countries of the Asia Pacific (China, Japan, India, and South Korea) and North American countries (the United States and Canada), as well as some African countries (Egypt, Nigeria, and Morocco) [22]. The global Polyphenols market size was valued at USD 2,160.69 million in 2021 and is expected to expand at a CAGR of 6.4% during the forecast period, reaching USD 3,135.56 million by 2027 [22]. By optimizing the use of these polyphenols within the feed and breeding sectors, there is potential for a significant expansion in their market size.

Plant polyphenols are currently considered a promising alternative to antibiotics and have great potential for application in livestock and poultry production. However, it should be emphasized that the catalogue of feed materials and feed additives issued by the Ministry of Agriculture and Rural Affairs of the People’s Republic of China includes only approximately 80 natural plant feed ingredients and 10 natural plant extract feed additives that are rich in plant polyphenols. The polyphenol compounds or extracts listed in the catalogue of feed additives, include curcumin, chlorogenic acid, propyl gallate, flavonoids from vine tea, daidzein, glycyrrhiza antioxidants, rosemary extract, *Eucommia ulmoides* leaf extract, tea polyphenols, and quercetagetin. Additionally, among more than 1,700 feed additives recently issued by the European Union, there are no more than 100 polyphenols or polyphenol-rich extracts. However, the selected polyphenol compounds represent just a very small fraction of the 8,000 plant polyphenols that have been identified to date. Therefore, more basic studies associated with product development and application need to be conducted in the future. In particular, governments in many countries have recently prioritized the development of natural plant feeds and actively encouraged the research and development of new products. Undoubtedly, natural plant feed ingredients will usher in a good development opportunity under the active guidance and support of the national governments. It is also of great strategic significance for the green and healthy breeding of swine.

## Bioactive properties of polyphenols

Polyphenols have a variety of health-promoting properties, including but not limited to antioxidant, anti-inflammatory, antibacterial, antiviral and immunomodulatory activities, microflora regulation, metabolic function, and intestinal protective properties. These health-promoting properties have been identified in a variety of animals and in vitro models.

### Antioxidant function

Under normal conditions, the production and elimination of reactive oxygen species (ROS) are under dynamic equilibrium. Once this dynamic equilibrium is disrupted, oxidative stress ensues, resulting in damage to biological macromolecules (including lipids, proteins, and nucleic acids), thereby exerting detrimental effects on animal growth performance and overall health [23, 24]. Polyphenols have been reported to prevent or reduce oxidative stress associated with various diseases, such as cardiovascular disease, diabetes mellitus type 2, neurodegenerative disorders, tissue injury, metabolic syndrome, inflammatory diseases, and infectious diseases [25–27]. In addition, polyphenols can relieve oxidative stress induced by certain hazardous substances, including hydrogen peroxide (H_2_O_2_), mycotoxins, and pesticides [28–30]. The beneficial effect of polyphenols on the antioxidant capacity can be attributed to their ability to scavenge ROS directly or indirectly by activating the antioxidant enzyme systems, thereby protecting the body from oxidative stress. Polyphenols are considered potent antioxidants and can effectively eliminate a variety of ROS, including superoxide anion, H_2_O_2_, hydroxyl radical, singlet oxygen, nitrogen oxide, and peroxynitrite. Specifically, magnolol showed an ability to scavenge H_2_O_2_ and superoxide anion generated from homocysteine-induced endothelial dysfunction in porcine coronary arteries [31]. Quercetin administration was shown to decrease hydroxyl radical levels in the hippocampus and cerebral cortex of mice [32] and accelerate the quenching of singlet oxygen in erythrocytes [33]. Curcumin exhibited significant peroxynitrite scavenging activity in RAW264.7 macrophages [34]. Additionally, catechin, cyanidin-3-glucoside, cyanidin-3-rutinoside, epicatechin, and protocatechuic acid were found to effectively eliminate peroxyl, hydroxyl, and peroxynitrite radicals in in vitro experiments [35]. These effects can be explained by the presence of conjugated double bonds or hydroxyl groups in polyphenols. As a result, they can donate hydrogen or electrons to neutralize free radicals and capture unpaired electrons of free radicals, thereby serving as an antioxidant by terminating the free radical chain reaction [36, 37]. For polyphenolic compounds, the more conjugated double bonds or hydroxyl groups they have, the greater their antioxidant capacity.

**Fig. 5:**
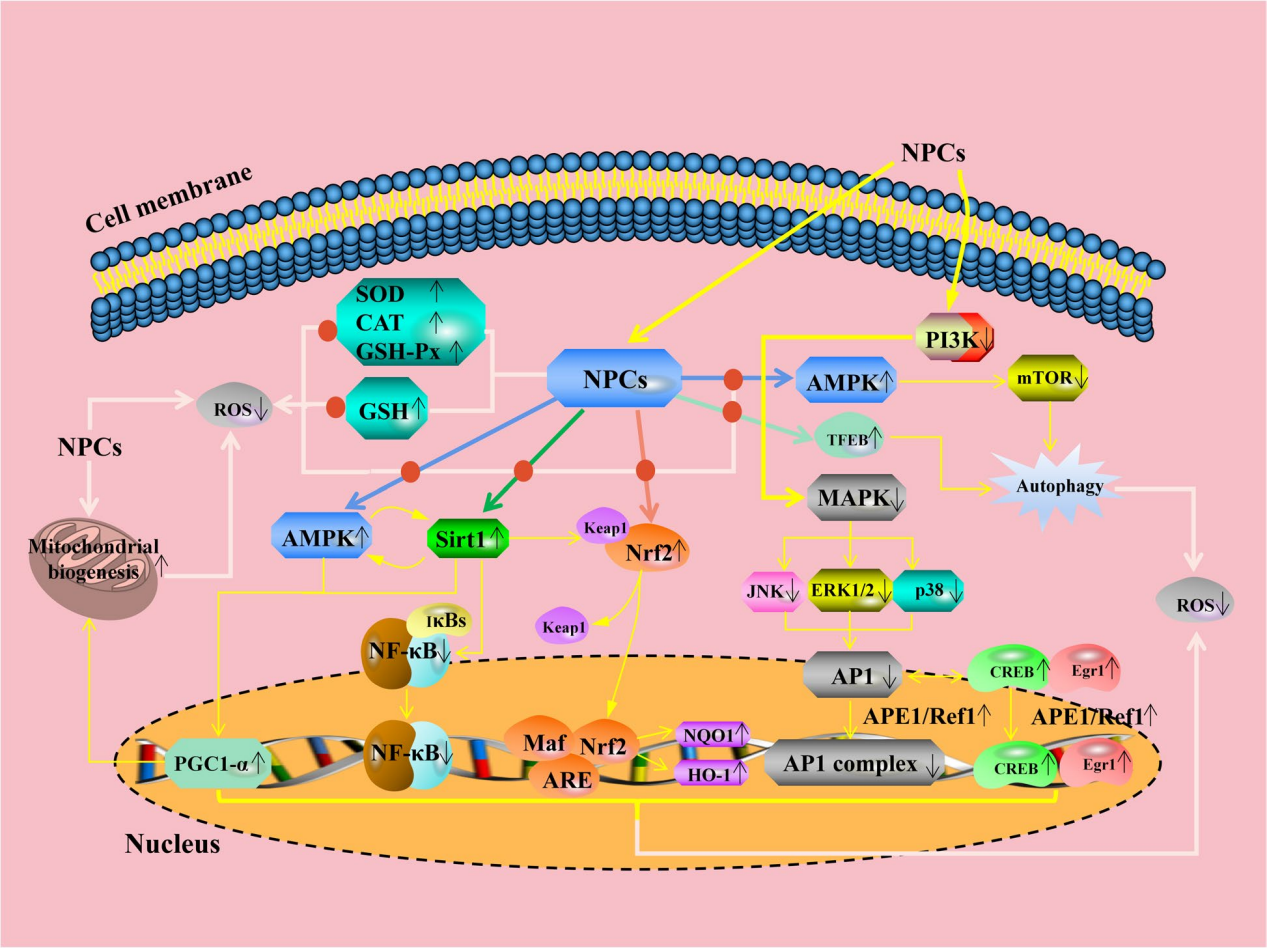
The antioxidant molecular mechanisms of plant polyphenols. NPCs can regulate the enzyme-mediated antioxidant defense system or the non-enzyme-dependent antioxidant defense system. It can also promote the expression of genes involved in mitochondrial biogenesis and/or enhance the expression of genes encoding antioxidant enzymes involved in ROS scavenging via regulating the AMPK, Sirt1, Nrf2, MAPK, and AP1 signaling pathways, etc. NPCs can induce autophagy via regulating mTOR-dependent or TFEB-dependent pathways to scavenge ROS. AMPK: adenosine-monophosphate-activated protein kinase; AP1: activator protein 1; APE1: Apurinic/apyrimidinic endonuclease 1; ARE: antioxidant responsive element; CAT: catalase; CREB: cAMP response element binding; Egr1: early growth response factor 1; ERK1/2: extracellular signal-regulated kinase 1/2; GSH: glutathione; GSH-Px: glutathione peroxidase; HO-1: heme oxygenase 1; IκB: NF-κB inhibitor; JNK: c-Jun N-terminal kinase; Keap1: Kelch-like ECH-associated protein 1; Maf: musculoaponeurotic fibrosarcoma; MAPK: mitogen-activated protein kinases; mTOR: mammalian target of the rapamycin; NF-κB: nuclear factor-kappa B; NPCs: Natural phenolic compounds; NQO1: NAD(P)H:quinone oxidoreductase 1; Nrf2: nuclear factor erythroid 2-related factor 2; p38: p38 MAPK; PGC-1α: proliferator-activated receptor γ coactivator‐1α; PI3K: phosphatidylinositol 3-kinase; Ref1: redox effector 1; ROS: reactive oxygen species; Sirt1: sirtuin1; SOD: superoxide dismutase; TFEB: transcription factor EB

Increasing evidence indicates that another way by which polyphenols exert their antioxidant effects is through the activation of the antioxidant enzyme system. Feeding piglets polyphenol-rich grape pomace increased the activities of catalase (CAT), superoxide dismutase (SOD), and glutathione peroxidase (GSH-Px) in the liver, kidneys, and spleen [38]. Similar results were obtained in an in vitro model indicating that ferulic acid protected intestinal porcine epithelial cell line-J2 (IPEC-J2) cells against deoxynivalenol (DON)-induced oxidative stress by increasing the activity of SOD and levels of glutathione (GSH) [39]. Nuclear factor erythroid 2-related factor 2 (Nrf2) is a transcription factor that can regulate the transcription of antioxidant response element-related genes [40, 41]. Many studies have demonstrated that polyphenols can enhance the antioxidant capacity and reduce oxidative stress by regulating the Nrf2 signaling pathway [42, 43]. In addition, quercetin was found to increase the mRNA levels of *Nrf2* and its downstream genes in IPEC-J2 cells, thereby alleviating DON-induced intestinal oxidative damage [44]. A study in piglets with paraquat-induced intestinal oxidative damage has found that ellagic acid increased serum SOD activity, reduced serum malondialdehyde (MDA) levels, and upregulated the expression levels of heme oxygenase 1 (*HO-1*) and NAD(P)H:quinone oxidoreductase 1 in small intestinal mucosa by facilitating Nrf2 nuclear translocation, and thus alleviated oxidative injury [45]. Protocatechuic acid was reported to ameliorate palmitic acid-induced oxidative damage in endothelial cells through activating the Nrf2/HO-1 signaling pathway via the activation of the adenosine-monophosphate-activated protein kinase (AMPK)-dependent signaling pathway [46]. Peng et al. [47] found that quercetin increased the mRNA expressions of antioxidant enzyme activities, improved mitochondrial function, and reduced ROS production, thereby alleviating lipopolysaccharide (LPS)-induced oxidative stress in RAW264.7 cells. They suggested that all these regulatory changes observed were related to the activation of the sirtuin 1 (Sirt1)/proliferator-activated receptor γ coactivator‐1α (PGC-1α) signaling pathway. In a diabetes‑induced atherosclerosis mouse model, quercetin has been found to inhibit oxidative stress and inflammatory responses via modulation of the AMPK/Sirt1/NF‑κB signaling pathway [48]. Additionally, resveratrol was demonstrated to improve systemic oxidative stress by activating the AMPK/Sirt1/Nrf2 signaling pathway [49]. Recently, curcumin was found to protect mouse ovaries from oxidative damage via regulating the AMPK/mammalian target of the rapamycin (mTOR) signaling pathway mediated autophagy [50]. Zhou et al. [51] reported that resveratrol promoted the formation of autophagosomes and lysosomes as well as their fusion into an autolysosome, thereby attenuating endothelial oxidative injury. They also found that all these changes hinge upon the activation of transcription factor EB (TFEB). Generally, polyphenols protect against oxidative stress mainly by the following mechanisms (as summarized in Fig. 5): (1) Directly scavenging free radicals; (2) Increasing the activities of endogenous antioxidant enzymes; (3) Promoting the expression of genes involved in mitochondrial biogenesis and/or enhance the expression of genes encoding antioxidant enzymes involved in ROS scavenging via regulating the AMPK, Sirt1, and Nrf2 signaling pathways, etc.; (4) Inducing autophagy via regulating mTOR-dependent or TFEB-dependent pathways.

### Anti-inflammatory and immunomodulatory activities

Inflammation is a defensive response to infections and tissue injury that serves as a protective mechanism against a wide range of exogenous and endogenous stimuli. While proper inflammation can protect the body against infection and damage, excessive inflammation may lead to body injury. Polyphenols have demonstrated efficacy in reducing inflammation by limiting the production and secretion of inflammatory factors [52, 53]. In a study conducted by Wen et al. [54], the inclusion of caffeic acid in the diet of LPS-challenged piglets decreased the serum levels of inflammatory markers, including interleukin-1 β (IL-1β), interleukin-6 (IL-6), and tumor necrosis factor α (TNF-α). Similarly, pterostilbene treatment downregulated the mRNA expression levels of *IL-1β*, *IL-6*, and *TNF-α* in the liver of LPS-challenged piglets [55]. The significant anti-inflammatory effects of polyphenols were inseparable from the regulation of nuclear factor-kappa B (NF-κB), mitogen-activated protein kinases (MAPK), and activator protein 1 (AP1) signaling pathways. Upon exposure to various stimuli, such as LPS, viruses, bacteria, or oxidative stress, the activation of NF-κB, MAPK, and AP1 signaling pathways ensues, thus inducing the inflammatory response. These pathways mediate the regulation of the expression of genes associated with inflammation and promote the production of pro-inflammatory factors [56, 57]. Endale et al. [58] found that quercetin disrupted phosphatidylinositol 3-kinase (PI3K) and myeloid differentiation factor-88 association, thereby inhibiting the MAPK/AP1 and IκB kinase/NF-κB-induced production of inflammatory cytokines and associated inflammatory mediators in LPS-induced RAW264.7 cells. Magnolol was reported to exert their anti-inflammatory effect through the regulation of the transcription of genes associated with inflammation by inhibiting the activation of the NF-κB and MAPK signaling pathways [59]. Moreover, magnolol was able to reverse the IL-1β-induced increase in the expression of genes related to inflammation [*IL-6*, cyclooxygenase-2 (*COX-2*), matrix metalloproteinases (*MMPs*), etc.] in a rat arthritis model by blocking the AP1 signaling pathway, thereby alleviating arthritis [60]. Similarly, resveratrol reduced the production and secretion of inflammatory mediators through the inhibition of the MAPK and AP1 pathways, thereby exerting potent anti-inflammatory effects [61]. In LPS-challenged RAW264.7 macrophages, pomegranate peel polyphenols, punicalagin, and ellagic acid reduced the levels of pro-inflammatory cytokine (TNF-α, IL-1β, and IL-6) and inflammatory mediators [nitric oxide, prostaglandin E2, COX-2, and inducible nitric oxide synthase (iNOS)], thereby alleviating inflammation [62]. They surmised that the anti-inflammatory effect might be due to the inhibition of MAPK activation. Peroxisome proliferator-activated receptor γ (PPARγ) is a ligand-activated transcription factor that can inhibit the transcription of inflammation-related genes by interfering with NF-κB and AP1 signaling pathways [63, 64]. Galangin has been reported to reduce the mRNA expression levels of pro-inflammatory cytokines (*TNF-α* and *IL-6*) and inflammatory mediators (*iNOS*, *COX-2*, and *MMPs*) in LPS-stimulated microglia by acting as an agonist of PPARγ [65]. In a study conducted on mouse skin, magnolol was found to reduce the mRNA expression levels of *iNOS* and *COX-2* by suppressing the activation of the NF-κB pathway through inhibiting the activation of the MAPK and PI3K/protein kinase B (AKT) pathways [66]. Furthermore, magnolol has been found to promote the activation of the PI3K/Nrf2/HO-1 signaling pathway and suppress the activation of Nod-like receptor pyrin domain containing 3 (NLRP3) in a mouse model of alcoholic liver injury, ultimately leading to a significant reduction in the mRNA expression levels of *TNF-α* and *IL-1β* [67]. Tian et al. [68] discovered that resveratrol inhibited the NF-κB pathway activation in a mouse model of non-alcoholic fatty liver disease via activating the AMPK-Sirt1 pathway, therefore improving hepatic inflammatory response. In addition to the above signaling pathway, polyphenols, like quercetin reduced the adhesion and migration of leukocytes and the activation of T cells by inhibiting the protein expression levels of vascular cell adhesion molecule 1 and cluster of differentiation 80, thereby improving H_2_O_2_-induced endothelial cell inflammation [69]. Collectively, these findings indicated that polyphenols were able to regulate the levels and the mRNA expression of inflammatory mediators and pro-inflammatory cytokines to protect against inflammation, mainly by activating the PPARγ and Nrf2 signaling pathways, inhibiting the MAPK, NF-κB, and AP1 signaling pathways, and suppressing the NLRP3 inflammasome activation.

**Fig. 6:**
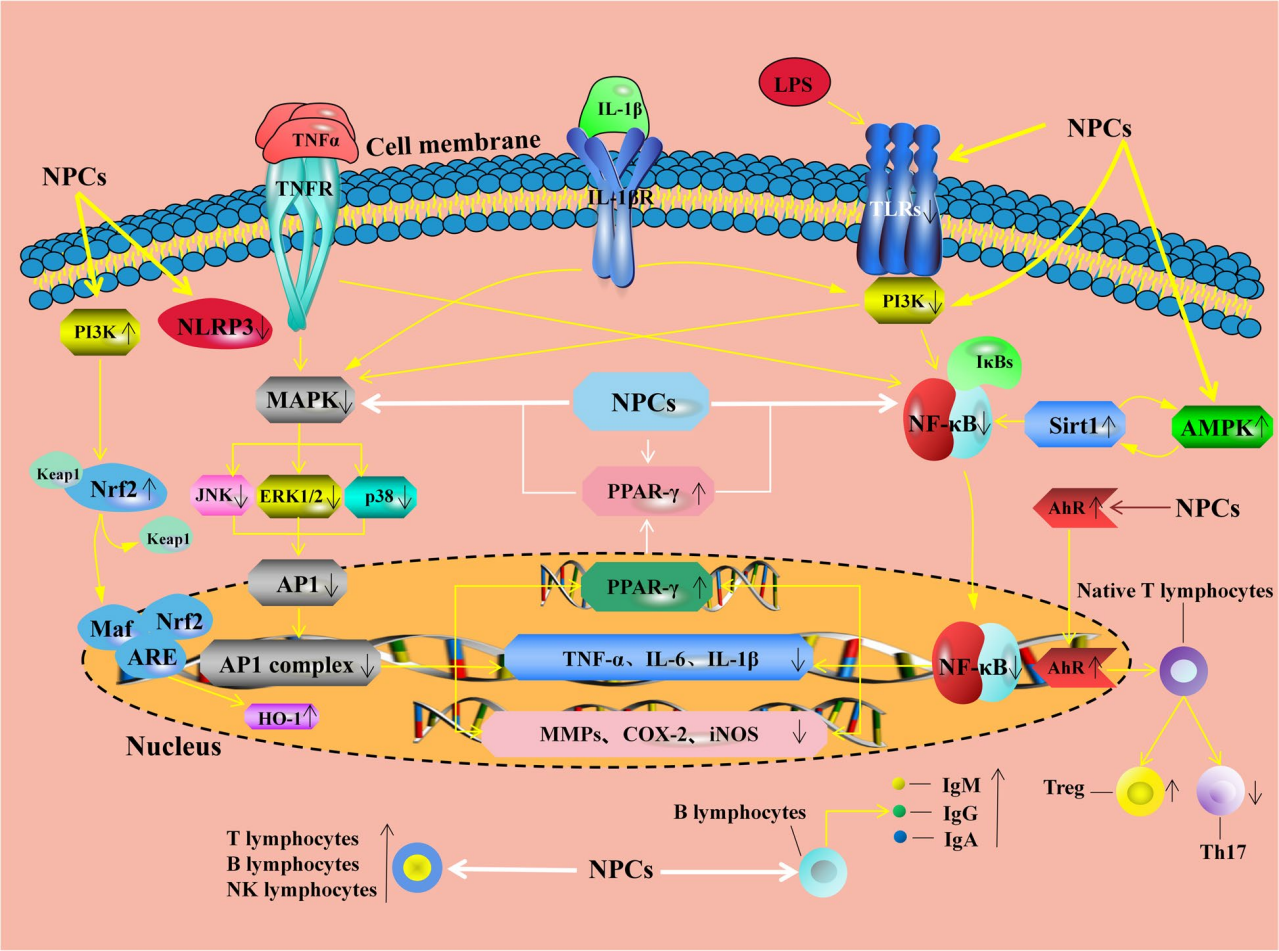
The anti-inflammatory and immunomodulatory molecular mechanisms of plant polyphenols. NPCs can regulate the production and secretion of inflammatory mediators and pro-inflammatory cytokines by activating the AMPK, Sirt1, PPARγ, and Nrf2 signaling pathways, inhibiting the MAPK, NF-κB, and AP1 signaling pathways, and suppressing the NLRP3 inflammasome activation. NPCs can increase the number of T lymphocytes, B lymphocytes, and NK lymphocytes. NPCs can promote the differentiation and maturation of B lymphocytes and act as an AhR agonist to induce T lymphocytes to differentiate into Tregs. AhR: aryl hydrocarbon receptor; AMPK: adenosine-monophosphate-activated protein kinase; AP1: activator protein 1; ARE: antioxidant responsive element; COX-2: cyclooxygenase-2; ERK1/2: extracellular signal-regulated kinase 1/2; HO-1: heme oxygenase 1; IκB: NF-κB inhibitor; IgA: immunoglobulin A; IgG: immunoglobulin G; IgM: immunoglobulin M; IL-1β: interleukin-1 β; IL-1βR: interleukin-1 β receptor; IL-6: interleukin-6; iNOS: inducible nitric oxide synthase; JNK: c-Jun N-terminal kinase; Keap1: Kelch-like ECH-associated protein 1; LPS: lipopolysaccharide; Maf: musculoaponeurotic fibrosarcoma; MAPK: mitogen-activated protein kinases; MMPs: matrix metalloproteinases; NF-κB: nuclear factor-kappa B; NLRP3: Nod-like receptor pyrin domain containing 3; NPCs: Natural phenolic compounds; Nrf2: nuclear factor erythroid 2-related factor 2; p38: p38 MAPK; PI3K: phosphatidylinositol 3-kinase; PPARγ: peroxisome proliferator-activated receptor γ; Sirt1: sirtuin1; TLRs: Toll-like receptors; TNF-α: tumor necrosis factor α; TNFR: TNF-α receptor

Besides their anti-inflammatory activity, polyphenols could play an immunomodulatory role by enhancing cellular and humoral immunity. For example, quercetin increased the serum immunoglobulin G (IgG) level in LPS-challenged pigs [70]. Resveratrol was reported to raise the serum IgG level in piglets challenged with a combination of *Escherichia coli* KCTC 2571 and *Salmonella enterica *serover Typhimurium [71]. It has also been reported that resveratrol promotes the development, maturation, proliferation, and transformation of T lymphocytes and increases the serum levels of immunoglobulin M (IgM) and IgG [72]. Additionally, tea polyphenols were found to promote the recovery of T lymphocyte proliferation and activation in piglets exposed to diquat [73]. Green tea catechin metabolites increased the activity of CD4^+^ T cells and the cytotoxic activity of NK cells in the spleen of mice [74]. In aged rats, cassia anthocyanidins increased the number of T and B cells, reduced ROS production, and thus enhanced immune function and prevented *Escherichia coli* infection [75]. In a dextran sulfate sodium (DSS)-induced colitis mouse model, quercetin was observed to downregulate neutrophil and macrophage activities and maintain the homeostasis between Treg and Th17 by activating aryl hydrocarbon receptor (AhR), thereby restoring the dynamic balance of the innate and adaptive intestinal immune systems [76]. These studies found that polyphenols are beneficial for strengthening the immune system of the host. Other studies reported that polyphenols can reduce detrimental immune responses. Green tea epigallocatechin-3-gallate reduced the differentiation of Th1, Th9, and Th17 cells and inhibited IL-6-induced suppression of Treg development, ultimately alleviating the autoimmune response in mice [77]. LPS-challenged mice exhibited reduced macrophage activation and dendritic cell maturation after fisetin treatment [78]. Similarly, in vitro experiments revealed that resveratrol prevented autoimmune reactions by inhibiting the maturation of human monocyte-derived dendritic cells [79]. The major anti-inflammatory and immunomodulatory mechanisms of plant polyphenols are presented in Fig. 6.

### Intestinal epithelial barrier protective function

The intestine serves not only as the main organ for digestion and absorption of nutrients, but also as the first barrier to prevent the passage of enteric pathogens, endotoxins, and antigens into the systemic circulation [80]. Thus, maintaining the integrity of the intestinal barrier function is critical for human and animal health. Intestinal barrier integrity is primarily maintained by the tight junction (TJ) structures formed by TJ proteins [81]. It has been demonstrated that *Escherichia coli* stimulation decreased *trans*-epithelial electrical resistance in Caco-2 cells, but this effect was reversed after treatments with anthocyanidins and procyanidin, suggesting recovery of the intestinal epithelial barrier integrity [82]. A study in piglets revealed that piglets fed a diet supplemented with chlorogenic acid exhibited enhanced intestinal barrier function, which was supported by increased expression of the *Occludin* gene in the duodenum, jejunum, and ileum [83]. Resveratrol was also found to improve intestinal barrier function in piglets by increasing the expression levels of the zonula occludin-1 (*ZO-1*) and interleukin-10 (*IL-10*) in the jejunum [84]. A study conducted by Gong et al. [85] showed that taxifolin reversed the LPS-induced decrease in the mRNA and protein expression levels of Claudin-1, ZO-1, and Occludin as well as an increase in the secretion of TNF-α, IL-1β, and IL-6, thereby protecting Caco-2 cells from LPS-induced impairment of barrier integrity. Also, pterostilbene alleviated intestinal barrier injury in DSS-induced mice by enhancing the distribution of ZO-1 and Occludin and increasing the mRNA and protein expression levels of ZO-1, Occludin, and Claudin-4 [86]. They surmised that the beneficial effects of taxifolin and pterostilbene on the intestinal barrier integrity in in vivo and in vitro models may be attributed to the inactivation of the NF-κB/myosin light chain kinase pathway. Protocatechuic acid and quercetin have been reported to reduce inflammation and barrier integrity damage in intestinal porcine epithelial cell line-J1 (IPEC-1) cells exposed to enterotoxigenic *Escherichia coli* (ETEC) by increasing the protein expression levels and enhancing the distribution of ZO-1, Occludin, and Claudin-1 and decreasing the mRNA and protein expression levels of TNF-α, IL-6, and interleukin-8 [87]. They suggested that the suppression of necroptosis and pyroptosis signaling pathways could be responsible for the effects of protocatechuic acid and quercetin on IPEC-1 cells. In conclusion, polyphenols could enhance intestinal barrier integrity or repair damaged intestinal barrier integrity in in vivo and in vitro models by enhancing the expression and distribution of TJ proteins as well as reducing inflammation and permeability.

### Antibacterial function

Pathogenic bacteria are the main cause of diarrhea, food poisoning, infections, and a severe threat to human and animal health worldwide [88, 89]. Polyphenols have been reported to have a broad-spectrum antibacterial activity. They can reduce bacterial adhesion, disrupt or alter cytoplasmic membrane permeability, and inhibit ribosome, nucleic acid, and protein synthesis, thereby inhibiting biofilm formation and ultimately affecting the growth, reproduction, and metabolism of pathogenic bacteria. In particular, green tea polyphenols showed the ability to inhibit *Streptococcus suis* infection by downregulating the expression of genes involved in virulence and growth [90]. In addition, epigallocatechin gallate inhibited the growth, hemolytic activity, and biofilm formation of *Streptococcus suis* by inhibiting the expression of proteins associated with DNA replication, synthesis of cell wall and cell membrane, and virulence [91]. Emodin was reported to inhibit the expression of some key proteins in the ATP-binding cassette transport system, carbohydrate metabolism pathway, and bacterial cell division by inhibiting the ribosome synthesis, resulting in the growth inhibition of *Haemophilus parasuis* [92]. They also found that emodin inhibited the adhesion and invasion of *Haemophilus parasuis* to porcine kidney cells and promoted the phagocytic activity of porcine alveolar macrophages against *Haemophilus parasuis*. A study by Guo et al. [93] showed that olive oil polyphenols increased cytoplasmic leakage and membrane depolarization, reduced bacterial protein and ATP levels, and suppressed the growth of *Salmonella *Typhimurium and *Staphylococcus aureus*. Some studies have shown that the population size and adhesion rate of ETEC in IPEC-J2 cells were decreased by treatment with tea polyphenols [94]. In addition, epigallocatechin gallate was found to suppress hemolysis, cell invasion, cell adhesion, and apoptosis by inhibiting the type III secretion system of enteropathogenic and enterohemorrhagic *Escherichia coli*, *Salmonella enterica* serovar Typhimurium, and *Yersinia pseudotuberculosis* [95]. Furthermore, polyphenols such as quercetin could bind to the ATP binding site of *Escherichia coli* and inhibit the supercoiling activity of DNA gyrase, leading to suppression of the gyrase ATPase activity, induction of DNA lysis, and ultimately exertion of antibacterial effects [96].

### Antiviral activity

Polyphenols have a wide range of antiviral activities that prevent infection and invasion by many swine-origin viruses. In Marc-145 cells, tea polyphenols were found to inhibit porcine reproductive and respiratory syndrome virus (PRRSV) infection by inhibiting the attachment, internalization, and release of PRRSV, limiting the synthesis of viral non-structural protein 2, and preventing the translocation of NF-κB p65 into the nucleus [97]. Similarly, procyanidins showed a strong antiviral effect in Marc-145 cells infected with PRRSV, which manifested as the inhibition of RNA synthesis, protein expression, and generation of virus following treatment with procyanidins [98]. Also, resveratrol treatment in piglets promoted resistance to pseudorabies virus (PRV) infection by inhibiting viral reproduction [99]. Moreover, theaflavin inhibited the African swine fever virus (ASFV) replication in primary porcine alveolar macrophages, and the potential mechanism was attributed to the activation of the AMPK signaling pathway [100]. Kaempferol inhibited ASFV protein and DNA synthesis by inducing autophagy in ASFV-infected Vero cells, thereby exerting an antiviral effect [101]. Chrysin and naringenin inhibited porcine epidemic diarrhea virus (PEDV) replication by interacting with the viral replicase proteins [102]. In addition, catechin epigallocatechin gallate interacted with porcine circovirus type 2 capsid protein to disturb the binding of the capsid to the cell surface receptor heparan sulfate, ultimately helping porcine kidney cells against circovirus invasion [103]. These studies clearly show that polyphenols prevent porcine virus infection by inhibiting viral attachment, internalization, replication, translation, and release, suggesting that polyphenols have significant application prospects in preventing and treating diseases in swine production.

### Metabolic regulation function

The metabolic regulatory effects of polyphenols have been extensively demonstrated in numerous studies. They have been found to prevent or successfully treat various metabolic-related diseases, including obesity and non-alcoholic fatty liver disease [104, 105]. A study in 3T3-L1 cells revealed that cyanidin-3-*O*-glucoside decreased lipid accumulation by enhancing insulin sensitivity through inhibition of the activation of the PPARγ and NF-κB pathways [106]. Baicalin was reported to reduce adipogenesis in 3T3-L1 cells by inhibiting the transcription of genes involved in adipogenesis (e.g., *PPARγ* and CCAAT/enhancer binding protein α) through the activation of the β-catenin pathway [107]. Furthermore, studies in human fat cells have demonstrated that resveratrol reduces lipid accumulation by activation of the β-adrenergic system [108]. In vitro, resveratrol has been shown to inhibit the proliferation and differentiation of swine preadipocytes by regulating Sirt1 [109]. In finishing pigs, dietary supplementation with flavonoids from mulberry leaves decreased the plasma concentrations of total cholesterol, free fatty acids, and triglycerides (TGs), inhibited fat production in dorsal subcutaneous adipose tissue, and increased the contents of C18:3n3 (α-linolenic acid, ALA) and n-3 polyunsaturated fatty acid (PUFA) in dorsal subcutaneous adipose tissue, abdominal subcutaneous adipose tissue, and visceral adipose tissue [110]. They suggested that the improvement of fatty acid distribution and lipid metabolism can plausibly be attributed to the modulation of the PPARγ-liver X receptor α (LXRα)-ATP binding cassette subfamily A member 1 (ABCA1) signaling pathway. Dietary grape seed proanthocyanidin extract supplementation raised the gene expression levels of carnitine-palmitoyl transferase 1 (*CPT1*), peroxisome proliferator-activated receptor α (*PPARα*), hormone-sensitive lipase (*HSL*), *PGC-1α*, and *Sirt1* in the liver and lowered acetyl-CoA carboxylase (*ACC*) gene expression level by activating the AMPK signal, which in turn accelerated fatty acid oxidation and lipolysis and decreased lipogenesis, thus improving hepatic lipid metabolism in finishing pigs [111]. In a mouse model of fatty liver induced by a high-fat diet, a reduction in diet-induced obesity and hepatic fat deposition was observed in mice offered with blue honeysuckle extract treatment [112]. Wu et al. [113] discovered that polyphenols extracted from *Lonicera caerulea* L. berries prevented the progression of non-alcoholic fatty liver disease into a more severe condition called non-alcoholic steatohepatitis by modulation of the ratio of Firmicutes to Bacteroidetes. Together, these results revealed that polyphenols have excellent activities to improve metabolic dysbiosis.

## Application of polyphenols in swine production

Due to the aforementioned multiple of bioactivities of polyphenols, they have been extensively used as effective feed additives in swine production (Additional file 1). In this section, we examine the effect of dietary supplementation with polyphenols on swine production and discuss the underlying mechanisms in detail.

### Growth performance

Evidence is growing that the polyphenol compounds derived from different plants are being recognized as effective additives for improving growth performance and reducing diarrhea in animals. For piglets, most evidence indicates that polyphenols have strong growth-promoting effects, especially in piglets under various stress conditions. Intrauterine growth retardation (IUGR) is a common pregnancy complication with low birth weight and growth retardation of offspring due to uteroplacental insufficiency [114]. Some studies have shown that dietary supplementation with 400 mg/kg curcumin improved the average daily gain (ADG) and average daily feed intake (ADFI) or total feed intake in IUGR piglets [115, 116]. Wan et al. [117] found that the feed to gain (F/G) ratio of IUGR piglets was increased after ferulic acid treatment. Nevertheless, a study conducted by Zhang et al. [118] found no improved effects of dietary supplementation with 200 mg/kg curcumin on body weight (BW), ADG, and F/G ratio in IUGR piglets, but found a lower ADFI. Additionally, there were no significant effects on BW, ADG, ADFI, and F/G whether the IUGR piglets were fed diets supplemented with 300 mg/kg resveratrol or 300 mg/kg pterostilbene [119]. These controversial results regarding the effects of polyphenols on the growth performance of IUGR piglets may be related to the polyphenols species, supplementation levels, and feeding duration. Furthermore, dietary supplementation with polyphenols reversed the adverse effects of oxidative stress, mycotoxin, or pathogens on the growth performance of piglets. In diquat-induced oxidative stress models, the BW, ADG, and ADFI of piglets were improved by dietary supplementation with 1,000 mg/kg puerarin or chlorogenic acid [120–122]. DON is a type of mycotoxin widely distributed in cereal-based foods/feed that usually has a wide range of toxic effects in humans and other animals, such as oxidative stress, inflammation, growth suppression, and even death [114]. The BW, ADG, and ADFI were increased, and the F/G ratio was decreased by dietary supplementation with 1,000 mg/kg baicalin or 300 mg/kg resveratrol in piglet diets containing DON [123–125]. For pathogens, dietary supplementation with 0.5 mg/kg resveratrol increased the BW and survival rate in a piglet model infected with PEDV [126]. Dietary supplementation with resveratrol at doses of 10, 30, and 90 mg/kg increased the BW and survival rate in a piglet model infected with PRV [99]. Higher ADG with lower F/G and diarrhea rate was observed in a piglet model infected with transmissible gastroenteritis virus (TGEV) fed a diet containing 400 mg/kg eugenol [127]. In LPS-challenged piglets, increased BW at d 28 and ADG from d 21 to 28 were observed in piglets fed diets supplemented with 500 mg/kg caffeic acid [54].

Most of the evidence indicates that dietary polyphenols have limited efficacy in enhancing growth performance of pigs in other growth stages, especially in finishing pigs. For instance, dietary supplementation with naringin at doses of 500, 1,000, and 1,500 mg/kg, grape seed proanthocyanidin extract at a dose of 200 mg/kg, resveratrol at doses of 200, 400, and 600 mg/kg, apple polyphenols at doses of 400 and 800 mg/kg or red-osier dogwood polyphenol extract at a dose of 5,000 mg/kg had no significant effects on ADG, ADFI, and F/G ratio in finishing pigs [128–132]. Additionally, Luo et al. [133] found that dietary supplementation with thymol at a dose of 100 mg/kg had no noticeable effects on BW and F/G ratio in finishing pigs, but a reduction in BW gain was noted, indicating that, to some extent, thymol may have a negative effect on growth performance. Collectively, these results indicated that dietary supplementation with polyphenols prevented adverse stress effects or infections and thus promoted their growth and development. As the pig matures, its digestive, immune systems, and gut microbiota gradually reach a fully developed and stable state, thereby enhancing the adaptability to both internal and external stimuli [134]. Accordingly, under good rearing conditions, it may be somewhat challenging to significantly improve the growth performance of a healthy pig by dietary supplementation with polyphenols, particularly in finishing pigs. This may explain why a majority of studies did not find increased growth performance of finishing pigs in response to supplementation with polyphenols. In addition, another popular explanation for these results may be the potential allocation of additional energy derived from feed absorption in response to polyphenol supplementation to enhance the immune system or other metabolic pathways, rather than solely focusing on growth promotion [135].

### Meat quality

There is abundant evidence indicating that phenolic compounds can improve the quality of pork either directly or indirectly. It has been reported that the dietary supplementation with daidzein at a dose of 62.5 mg/kg reduced drip loss and shear force of the *longissimus thoracis* (LT) muscle of growing-finishing pigs [136]. The value for redness (a*) at 45 min and 48 h and the value for lightness (L*) at 24 h in the *longissimus dorsi* (LD) muscle were increased and decreased, respectively, in growing-finishing pigs fed with dihydromyricetin at doses of 100, 300, and 500 mg/kg [137]. Liu et al. [138] reported that dietary supplementation with flavonoids from mulberry leaves at doses of 400 and 800 mg/kg reduced the shear force and a* value, and increased the marbling score in the *longissimus lumborum* (LL) muscle of finishing pigs. Dietary supplementation with chlorogenic acid at a dose of 400 mg/kg for 30 d, reduced the yellowness (b*) value in the LD muscle of finishing pigs [139]. The dietary supplementation with chlorogenic acid at a dose of 50 mg/kg increased the pH value at 24 h in the LT muscle of finishing pigs [140]. The values for L* and b* in the LD muscle of finishing pigs were decreased in pigs fed a diet supplemented with apple polyphenols at a dose of 800 mg/kg [131]. Meat pH is an important indicator of meat quality as it is related to shelf life, color, and water-holding capacity of meat. The value of pH is mainly determined by the degree of muscle glycolysis after animal slaughter, with a swift decrease in pH potentially resulting in protein denaturation, which, in turn, can contribute to the development of paleness and reduce the water-holding capacity of meat, thereby reducing its overall nutritional value [141, 142]. Meat color is the main factor affecting consumer preferences for meat products, due to its ability to serve as a barometer to assess both the freshness and overall integrity of the meat [143]. The shear force is negatively correlated with meat tenderness, affecting the sensory quality of meat and consumer acceptance [144]. Furthermore, meat characterized by low water-holding capacity tends to have poor visual aesthetics, ultimately leading to reduced consumer acceptance and a decline in sales [145]. Consequently, the conducive effects of polyphenols on the quality of pork have great economic significance.

Intramuscular fat (IMF) and inosinic acid (IMP) are also important indicators affecting meat quality. IMF is one of the key indices of meat quality, which is positively correlated with the flavor, juiciness, and tenderness of meat [146]. IMP is considered an umami flavor enhancer, as it can affect the freshness of meat after cooking [147]. Studies have shown that dietary supplementation with polyphenols promoted the deposition of IMF and IMP in muscle. The dietary supplementation with chlorogenic acid at doses of 200, 400, and 800 mg/kg, apple polyphenols at doses of 400 and 800 mg/kg, or naringin at a dose of 1.5 g/kg increased the IMP content in the LD muscle of finishing pigs [128, 131, 139]. This result can be attributed to the inhibition of IMP degradation by polyphenols, as indicated by a reduction in the gene expression of 5′-nucleotidase cytosolic II [140]. In addition, dietary supplementation with daidzein at a dose of 62.5 mg/kg or chlorogenic acid at a dose of 100 mg/kg increased the IMF content in the LT muscle of growing-finishing pigs [136, 140]. Also, supplementation with resveratrol at a dose of 600 mg/kg led to an increase in IMF content in the LD muscle of growing-finishing pigs [148]. The regulatory effect of polyphenols on IMF content in the muscles may be attributed to the regulation of lipid metabolism, such as increasing the expression of lipid synthesis-related genes (fatty acid synthase, *ACC1*, *PPARγ*, and *CPT1*) and decreasing the expression of lipid lipolysis-related genes (*HSL* and *PPARα*) [136, 140, 148].

Polyphenols have been reported to improve meat quality by improving amino acid compositions and fatty acid profiles in the muscles of pigs. Dietary supplementation with chlorogenic acid at a dose of 100 mg/kg increased the contents of total amino acids and flavor amino acids in the LT muscle of growing-finishing pigs [140]. Additionally, dietary supplementation with chlorogenic acid or apple polyphenols at a dose of 800 mg/kg increased the contents of flavor amino acids, essential amino acids, and total amino acids in the LD muscle of finishing pigs [131, 139]. It has been suggested that the mechanism by which polyphenols promote the accumulation of these amino acids in the muscles may involve the modulation of the AKT/mTOR signal [131, 135, 149]. Higher C22:6n-3 (docosahexaenoic acid, DHA) and ∑n-3 PUFA levels, and ratio of ∑PUFA/∑saturated fatty acid (SFA) were found in the LD muscle of finishing pigs fed a diet supplemented with apple polyphenols at a dose of 400 mg/kg [131]. Moreover, a diet supplemented with grape seed proanthocyanidin extract at a dose of 200 mg/kg increased the contents of crude protein, ALA, C18:2n6 (linoleic), C20:5n3 (eicosapentaenoic, EPA), ∑PUFA and ∑n-3 PUFA, as well as the ratio of ∑PUFA/∑SFA in the LD muscle of finishing pigs [129]. Previous studies have shown that dietary supplementation with flavonoids from mulberry leaves increased the contents of ALA and ∑n-3 PUFA and reduced the ratio of ∑n-6 PUFA/∑n-3 PUFA in the LL muscle of finishing pigs [138]. They suggested that the mechanism by which dietary flavonoids from mulberry leaves regulate fatty acid levels in pork was associated with the regulation of the PPARγ-LXRα-ABCA1 pathway. Additionally, the mechanism by which polyphenols promote PUFA deposition in muscle, especially n-3 PUFA, likely involves the upregulation of gene expression for fatty acid desaturase (∆5 and ∆6) and fatty acid elongase, as well as the activation of Nrf2 pathway [150]. Pork with higher levels of ∑n-3 PUFA and a beneficial ∑PUFA/SFA ratio, as well as a lower ratio of ∑n-6/∑n-3 PUFAs is readily accepted and desirable by consumers due to its superior nutritional profile and health-enhancing properties [151, 152]. These results demonstrated that dietary polyphenols can contribute to the production of high-quality pork.

Furthermore, the beneficial effect of polyphenols on the quality of pork was associated with their antioxidant activities and capacity to regulate the type of muscle fibers. The enhanced antioxidant capacity was found to contribute to the improvement of the meat quality traits, including muscle pH, tenderness, drip loss, and meat color [153]. Indeed, the role of dietary polyphenols as antioxidants in pig muscles has been widely reported. Polyphenols such as naringin, dihydromyricetin, curcumin, resveratrol, etc., have been shown to increase antioxidant enzyme activities and the expression of related genes and proteins, and reduce the levels of oxidative injury biomarkers (including MDA, thiobarbituric acid reactive substance, and protein carbonyl) by activating the Nrf2 signaling pathway, ultimately improving oxidative stability and the quality of pork [111, 118, 128, 129, 140, 154–158]. Importantly, recent research has revealed that polyphenols have the potential to regulate the type of muscle fibers. The muscle fiber type was found to be closely related to postmortem metabolic rate and meat quality traits. According to the main myosin heavy chains (MyHC) isoforms found in adult mammalian skeletal muscles, there are four types of muscle fibers, namely MyHC I in slow-oxidative type I, MyHC IIa in fast oxide-glycolytic type IIa, MyHC IIb in fast glycolytic type IIb, and MyHC IIx in fast glycolytic type IIx [159, 160]. A higher content of IIb fibers in pork was associated with enhanced glycolytic capacity, leading to decreased water-holding ability and rapid postmortem pH decline, ultimately contributing to inferior pork quality [161, 162]. In contrast, pork with higher contents of MyHC I and MyHC IIa fibers exhibited enhanced pork quality and were correlated with higher water-holding capacity and tenderness [163]. Moreover, dietary supplementation with daidzein at a dose of 62.5 mg/kg increased the *MyHC I* mRNA level and decreased the *MyHC IIb* mRNA level in the LD muscle of growing-finishing pigs [136]. Dietary supplementation with ellagic acid at doses of 75 and 150 mg/kg increased the slow MyHC protein abundance, decreased the fast MyHC protein abundance, increased the number of slow-twitch muscle fibers, and reduced the number of fast-twitch muscle fibers in the LT muscle of growing-finishing pigs [164]. In addition, dietary supplementation with ferulic acid at a dose of 25 mg/kg decreased the proportion of fast and glycolytic muscle fibers in the LT muscle of finishing pigs [165]. Meanwhile, dietary supplementation with resveratrol at doses of 200, 400, and 600 mg/kg elevated the mRNA levels of *MyHC I* and *MyHC IIa*, and downregulated the mRNA level of *MyHC IIb* in the LT muscle of finishing pigs [130]. Also, dietary supplementation with naringin at doses of 0.5, 1, and 1.5 g/kg increased *MyHC IIa* mRNA level and decreased *MyHC IIb* mRNA level in the LD muscle of finishing pigs [128]. In addition, the levels of certain enzymes in muscle can be used to assess metabolic fiber type changes. For example, a muscle with higher activities of succinate dehydrogenase (SDH) and malate dehydrogenase (MDH) tends to have a relatively higher percentage of oxidative myofibers, while a muscle with higher lactate dehydrogenase (LDH) activity tends to have a higher proportion of glycolytic type of muscle fiber [166]. Abundant evidence has shown that certain polyphenols (dihydromyricetin, grape seed proanthocyanidin extract, ellagic acid, resveratrol, apple polyphenols, etc.) increased the SDH and MDH activities in skeletal muscle from different growth phases of pigs, while at the same time decreasing the LDH activity in skeletal muscle [129, 130, 137, 155, 164, 167, 168]. Together, these results indicate that polyphenols can improve the quality of pork by promoting the transformation of fast glycolytic muscle fibers into slow oxidative muscle fibers. The regulatory effect of polyphenols on muscle fiber type may be exerted through the activation of the AMPK/Sirt1/PGC-1α pathway, thereby enhancing mitochondrial biogenesis and function, and ultimately promoting oxidative metabolism in skeletal muscle, which is a process that facilitates the transformation of fast, glycolytic fibers into slow oxidative fibers [130, 133, 137, 155, 164, 167, 168].

### Digestion and absorption of nutrients in the intestine and intestinal health

The intestine is the main site for the digestion and absorption of nutrients and is the largest immune organ in animals. Therefore, sustaining intestinal health is imperative for the growth and health of animals. Supplementation of the swine diet with polyphenols has various beneficial effects on intestinal health through a variety of ways, including promoting intestinal development, enhancing nutrient transport across the intestinal barrier, improving intestinal morphology, regulating the composition of the gut microbiota, reducing intestinal inflammation, and maintaining optimal redox balance within the intestine.

#### Digestion and absorption of nutrients in the intestine

The basic physiological function of the intestine is digestion and absorption of nutrients. Thus, the digestive and absorptive capacities serve as key indicators to evaluate intestinal health. On the other hand, high-efficiency digestion and absorption of nutrients are also indispensable for the integrity of intestinal structure and normal physiological function.

Indeed, weaned piglets need an enormous amount of nutrients to meet the requirements for the rapid renewal and growth of the intestinal epithelium as well as their own overall growth and development [169]. Unfortunately, immature intestinal development and insufficient secretion and/or low activity of digestive enzymes result in diarrhea and subsequent failure to thrive in early-weaned piglets. Therefore, the maintenance of optimal digestive enzyme activities is of great significance to the healthy growth of piglets. Fang et al. [170] reported that dietary supplementation with grape seed procyanidins at doses of 40 and 70 mg/kg enhanced the activities of amylase and lipase in the jejunum of piglets. Also, Xu et al. [171] found that dietary supplementation with coated tannin at a dose of 1,500 mg/kg enhanced the activities of jejunal maltase and sucrase, as well as that of ileal trypsin. Furthermore, polyphenols have been shown to have the ability to rescue the decrease in the activities of intestinal digestive enzymes caused by stressors, such as LPS, diquat, etc. Emerging evidence shows that the oxidative stress-induced decrease in trypsin, sucrase, lipase, and α-amylase activities in the jejunum of piglets caused by oxidized soybean oil was reversed by dietary supplementation with resveratrol at a dose of 300 mg/kg [172]. Another study has shown that dietary supplementation with chlorogenic acid at a dose of 1,000 mg/kg increased the activities of sucrase, lactase, and maltase in the duodenum and jejunum, as well as alkaline phosphatase activity in the jejunum and ileum of diquat-challenged piglets [121]. Additionally, the jejunal lactase activity in LPS-challenged piglets [173] and the activities of lactase, sucrase, and maltase in the jejunum and ileum in diquat-challenged piglets [174] were improved by dietary supplementation with holly polyphenols at a dose of 250 mg/kg. Taken together, these findings revealed that polyphenols have the potential to enhance the activities of intestinal digestive enzymes, thereby facilitating the breakdown of dietary macromolecules (carbohydrates and proteins) into smaller molecular entities (such as glucose, small peptides, and amino acids) that can be readily absorbed by the host. As a result, ultimately the digestion and absorption of nutrients are accelerated while maintaining optimal intestinal health. These results may also partially explain why dietary polyphenols improved the diarrhea and growth performance of piglets regardless of their physiological conditions.

The integrity of the intestinal villus-crypt morphological structure is the cornerstone of nutrient digestion and absorption and plays an instrumental role in upholding the intestinal barrier function. The villus height (VH) in the jejunum and ileum and the VH to crypt depth (CD) ratio (V/C) in the jejunum of piglets were increased by dietary supplementation with *Eucommia ulmoides* flavones at a dose of 100 mg/kg [175]. Also, dietary supplementation with proanthocyanidin at doses of 30, 300, 600, and 1,200 mg/kg increased VH and V/C ratio in the duodenum and jejunum of piglets, but decreased CD in the duodenum [176]. Moreover, polyphenols were found to counter the impairment of intestinal morphology in piglets subjected to some stressors, such as exposure to PEDV, DON, LPS, etc. Dietary supplementation with baicalin at a dose of 1,000 mg/kg reversed the DON-induced decrease in VH and V/C in the jejunum and ileum of piglets [123]. Dietary supplementation with emodin at a dose of 300 mg/kg increased the V/C in the jejunum of LPS-challenged piglets [177]. Also, dietary supplementation with tannic acid-chelated zinc at a dose of 50 mg/kg increased VH, V/C, and villus surface area in the duodenum and jejunum, but decreased CD in the duodenum, jejunum, and colon of PEDV-infected piglets [178]. The above results indicated that polyphenols promoted intestinal villus development and restored impaired intestinal morphology and structure in piglets exposed to various stressors.

The intestinal nutrient transport is predominantly carried out by nutrient transporters located in the mucosa of the small intestine. Polyphenols could promote nutrient transporter expressions in the small intestine, thus enhancing the intestinal uptake and absorption of nutrients. For instance, the mRNA levels for the duodenal and jejunal fatty acid transport protein 1, jejunal and ileal fatty acid transport protein 4, and the ileal oligopeptide transporter 1 were upregulated by dietary supplementation with proanthocyanidin at doses of 30, 300, and 600 mg/kg in piglets [176]. Moreover, dietary supplementation with chlorogenic acid at a dose of 1 g/kg upregulated the mRNA expression levels of sodium-glucose transport protein 1 (*SGLT1*) and zinc transporter-1 in the duodenum of piglets and *SGLT1*, glucose transporter-2 (*GLUT2*), and divalent metal transporter-1 in the jejunum of piglets [179]. Furthermore, it has been reported that polyphenols could improve intestinal nutrient transport under abnormal physiological conditions. For instance, Chen et al. [121] reported that dietary supplementation with chlorogenic acid at a dose of 1 g/kg increased the mRNA expression levels of duodenal and jejunal *SGLT1* and jejunal *GLUT2* in diquat-challenged piglets. It has also been reported that dietary supplementation with eugenol at a dose of 400 mg/kg upregulated the gene expression of *GLUT-2* and cationic amino acid transporter 1 in the jejunum of TGEV-infected piglets [127]. Overall, these studies indicated that polyphenols contribute to more efficient digestion and absorption of nutrients by the animal’s intestine by enhancing digestive enzyme activity, improving morphology, and increasing intestinal nutrient transport.

#### Intestinal health

Polyphenols have also been reported to exert their intestinal health-promoting effects by maintaining intestinal barrier integrity, improving intestinal microbiota and metabolites, reducing intestinal inflammation, and enhancing intestinal antioxidant and immune capacity. An improvement in the intestinal health of piglets was observed in diets containing polyphenol-rich plant products from grape or hop, as indicated by the decrease in *Streptococcus* spp. and *Clostridium* Cluster XIVa as well as pro-inflammatory genes [180]. Dietary supplementation with embelin at doses of 400 and 600 mg/kg reduced intestinal inflammation by increasing the mRNA expression level of *IL-10* and reducing the mRNA expression levels of *IL-1β* and *NF-κB* in the jejunum and ileum of piglets [181]. The mechanism by which embelin reduces intestinal inflammation may be by suppressing the P300/CBP associating factor/NF-κB pathway. Dietary supplementation with curcumin or resveratrol (each at 300 mg/kg) led to a shift in the intestinal microbiota of piglets, including enrichment of *Lactobacillus* and reduction of *Escherichia coli* number in the jejunum and ileum of piglets [182]. Moreover, dietary supplementation with curcumin or resveratrol reduced the mRNA expression levels of toll-like receptor 4 (*TLR4*), *IL-1β*, and *TNF-α* and increased the mRNA expression level for *IL-10* and the concentrations of immunoglobulin A (IgA) and IgG [182]. Additionally, polyphenols have also been shown to improve intestinal health in pigs with abnormal physiological status. For instance, dietary supplementation with curcumin alleviated intestinal oxidative stress and inflammation in IUGR piglets by promoting the activation of the Nrf2 pathway [183]. Also, dietary supplementation with resveratrol at doses of 30 and 90 mg/kg upregulated the mRNA expression of TJ proteins and increased the activities and mRNA expression of antioxidant enzymes in the jejunum of diquat-challenged piglets by promoting the activation of the AhR/Nrf2 pathway [184]. Xu et al. [174] documented that dietary supplementation with holly polyphenols reduced intestinal oxidative stress and ferroptosis in diquat-challenged piglets by decreasing the protein expression level of transferrin receptor protein 1 and increasing the protein expression levels of heat shock protein β1, solute carrier family 7 member 11, and glutathione peroxidase 4 in the jejunum and ileum. In LPS-challenged piglets, dietary supplementation with caffeic acid at a dose of 500 mg/kg increased the gene expression levels of *Occludin*, *Claudin-1*, *ZO-1*, B-cell lymphoma-2 (*Bcl2*) but decreased the gene expression levels of *NF-κB*, *IL-6*, *IL-1β*, B-cell lymphoma-2-associated X protein (*Bax*), and *Fas* as well as the ratio of Bax/Bcl2 in the colon [54]. The authors concluded that the protective effects of caffeic acid on intestinal health may be achieved by improving intestinal microbiota and metabolic disorders, including increased abundance of *Alloprevotella* and *Prevotellaceae_UCG-001*, and improvement of bile acid metabolic disorders. Hu et al. [185] found that dietary supplementation with protocatechuic acid at a dose of 4,000 mg/kg increased the gene and protein expression levels of ZO-1 and Caudin 1 in the intestine of LPS-challenged piglets. They also found an improvement of the intestinal barrier integrity, which was closely associated with enrichment in the abundances of *Roseburia*, and *Desulfovibrio* and reduction in the abundances of *Prevotella 9*, *Prevotella 2*, *Holdemanella*, and *Ruminococcus torques*. In DON-challenged piglets, dietary supplementation with resveratrol at a dose of 300 mg/kg increased the gene and protein expression levels of TJ proteins, reduced oxidative stress and inflammation, and inhibited cell apoptosis in the jejunum [125]. They suggested that the protective effect of resveratrol on intestinal health might be associated with an increase in the butyrate content and *Roseburia* abundance and a reduction in *Bacteroides* and *unidentified Enterobacteriaceae* abundances.

### Hepatic function

As a major site for nutrient metabolism and detoxification, the liver plays an important role in maintaining body health. Numerous studies have shown that polyphenols can improve hepatic redox balance, maintain glucose and lipid metabolism homeostasis, and reduce hepatic damage. Fang et al. [170] reported that dietary supplementation with grape seed procyanidins at doses of 40 and 70 mg/kg increased the activities for total superoxide dismutase (T-SOD) and GSH-Px and the mRNA expression levels of *SOD*, *GSH-Px*, and *CAT* in the liver of piglets. It has been documented that dietary supplementation with pterostilbene at a dose of 300 mg/kg alleviated early weaning-induced hepatic injury and oxidative stress in piglets by ameliorating mitochondrial dysfunction and endoplasmic reticulum stress [186]. Additionally, dietary supplementation of piglets with ferulic acid at a dose of 4.5 g/kg promoted lipolysis and fatty acid oxidation by increasing the mRNA expression levels of *HSL*, *CPT1*, and *PPARα*, thereby decreasing the hepatic TG level [187]. Moreover, dietary supplementation of piglets with apple polyphenols at doses of 400 and 800 mg/kg improved hepatic lipid metabolism and reduced lipid deposition by regulating the mRNA expression levels of genes associated with fatty acid oxidation, uptake, and de novo synthesis [188]. Additionally, polyphenols have been found to improve liver injury and/or metabolism abnormality caused by IUGR, LPS, and pesticides in piglets. Qi et al. [189] reported that dietary supplementation with ellagic acid at doses of 50 and 100 mg/kg reduced paraquat-induced hepatic fibrosis, steatosis, and apoptosis, and alleviated hepatic oxidative stress and inflammation by suppressing the NF-κB signaling pathway and activating the Nrf2 signaling pathway. They also found that the protective effects of ellagic acid on paraquat-induced hepatic oxidative damage and inflammation are strongly associated with the increase in the relative abundances of *Lactobacillus reuteri* and *Lactobacillus amylovorus*. Dietary supplementation with curcumin at a dose of 400 mg/kg enhanced hepatic antioxidant capacity, reduced hepatic insulin resistance and lipid accumulation in IUGR piglets by activating antioxidant signaling pathways, regulating the mRNA expression levels of genes involved in the insulin-signaling pathway, and regulating lipid metabolism [115, 116]. A study in LPS-challenged piglets has demonstrated that dietary supplementation with pterostilbene at a dose of 300 mg/kg reduced cell apoptosis, oxidative stress, and inflammatory response in the liver [55]. The study concluded that these beneficial effects of pterostilbene may be achieved by suppressing the protein phosphatase 2 A/NF-κB/NLRP3 signaling pathway. In diquat-induced piglets, supplementation with holly polyphenols at a dose of 100 mg/kg improved hepatic morphology and antioxidant capacity by regulating the expression levels of genes associated with ferroptosis [190]. Another study in diquat-challenged piglets showed that dietary supplementation with pterostilbene at a dose of 300 mg/kg reduced hepatic oxidative stress, mitochondrial dysfunction, and cell apoptosis by activating the Nrf2 and Sirt1 signaling pathways [191]. Also, resveratrol had an antioxidant effect in rotavirus-infected piglets, which was evidenced by decreased hepatic MDA levels and increased T-SOD and GSH-Px activities in piglets that received 3, 10, and 30 mg/kg/d of a resveratrol dry suspension [192]. Collectively, these findings indicated that polyphenols can sustain the normal physiological function of the liver by maintaining the redox balance, improving metabolic homeostasis, reducing cell apoptosis, and ameliorating mitochondrial function.

### Odor emissions reduction

Odor pollution is one of the seven public nuisances prevalent worldwide. With the rapid development of the global livestock breeding industry, environmental pollution due to emission of malodorous substances during the livestock breeding process is becoming increasingly serious. Due to their adverse effects on animal and human health as well as the environment, odor emissions have gained much attention worldwide and pose serious challenges to the healthy and sustainable development of the livestock industry. The main malodorous compounds among odor emissions include ammonia, sulfur-containing compounds, volatile fatty acids and amines, indole derivatives, phenols, and other volatile compounds [193, 194]. The role of plant polyphenols in reducing malodorous emissions at the source and terminal has been well documented. For instance, ammonia emission was reduced by more than 95% and methane by up to ∼99% after mixtures of tannic acid and sodium fluoride were cultivated together with pig manure [195]. Whitehead et al. [196] achieved a remarkable reduction in the emissions of fecal hydrogen sulfide and methane after treating pig manure with quebracho tannins, as indicated by the reduction in sulfate-reducing bacteria and methane-producing bacteria. Another study indicated that adding a mixture of plant essential oils (containing cinnamaldehyde and thymol) to pig diets can improve nitrogen digestibility, and also reduce the emissions of ammonia and total fecal nitrogen and the levels of indole and 3-methylindole [197]. The study suggested that the reduction in the emissions of these compounds may be attributed to the inhibition of microbial protease and urease activities by the treatment with a mixture of essential oils. Dietary supplementation with tea polyphenols at a dose of 2 g/kg significantly reduced the emissions of fecal ammonia, p-cresol, phenol, and skatole in piglets, and these changes were accompanied by a higher prevalence of *Lactobacillus* and lower prevalence of Bacteroidaceae and *Clostridium perfrigens* [198]. Additionally, dietary supplementation with magnolol at a dose of 200 mg/kg improved nitrogen utilization efficiency of piglets and altered the structure and metabolic activity of fecal microbiota, as indicated by the enrichment of crude protein apparent digestibility, number of fecal *Lactobacillus* and *Ruminococcus*, as well as the reduction in serum urea nitrogen content and number of fecal *Escherichia coli* [199]. All these regulations led to a reduction in the fecal contents of phenol, p-cresol, skatole, putrescine, cadaverine, and total amines. Yan and Kim [200] found a reduction in fecal ammonia and hydrogen sulfide emissions in growing pigs fed a diet supplemented with eugenol or cinnamaldehyde at a dose of 1 g/kg. The authors attribute these findings, in part, to the growth inhibition of *Escherichia coli*. The mechanisms by which magnolol lowered skatole emission in pigs have been elucidated in a recent study conducted by Li et al. [201]. They found for the first time that the regulation of magnolol-driven microbiota elicits changes in tryptophan metabolism, resulting in a reduction of skatole formation in growing pigs. Together, these studies revealed that plant polyphenols have positive effects on the reduction of malodorous substance emissions at the source and terminal in pig breeding. However, the mechanisms by which polyphenols reduce odor emissions remain a subject of considerable debate, although it is widely accepted that they exert their regulatory effects through the following three mechanisms, namely improving nutrient utilization of the diet by pigs, regulating microbial community and enzyme activities, and inhibiting the related metabolic activities involved in the formation or production of odor substances.

### Reproductive performance and maternal regulation

Due to their significant antioxidant, anti-inflammatory, and metabolic regulatory effects, polyphenols are widely used in sow nutrition. There is evidence indicating that, in sows, dietary supplementation with polyphenols effectively improved the growth and development of fetus and offspring, the health status of sows, placenta, and offspring, and the lactation performance of sows.

#### Lactation performance and milk quality

Breast milk is an important source of energy, nutrients, and immunity for suckling piglets. Thus, improving the breast milk quality benefits the growth and health status of suckling piglets.

Lactation is a highly energy-demanding process that generates significant amounts of free radicals, leading to oxidative stress in sows [202]. An inverse relationship between oxidative stress and lactation performance in sows has been reported [202]. Polyphenols were found to improve antioxidant function in sow mammary glands, as revealed by the antioxidant status of colostrum and milk. Dietary supplementation with resveratrol at a dose of 300 mg/kg during gestation and lactation improved the colostrum and milk antioxidant status, as indicated by the increased SOD level in colostrum and CAT level in 21-d milk, as well as the decreased MDA and H_2_O_2_ levels in 21-d milk [203].

Polyphenols also improved the immune function of the mammary glands of sows, as reflected by the immune status in colostrum and milk. Sows fed diets supplemented with 200 and 300 mg/kg of grape seed polyphenols from d 80 of gestation to d 21 of lactation had higher IgM and IgG levels in colostrum [204]. Higher IgA and IgG levels in colostrum and 17-d milk were also observed when sows received dietary supplementation with garcinol, at a dose of 600 mg/kg, from late gestation to postpartum [205].

Moreover, polyphenols have been reported to improve the lactation yield of sows and the nutrient composition in the colostrum and milk. Sows fed with 40 g/d of silymarin from day 108 of gestation to d 20 of lactation had higher protein and urea contents in milk on d 18 of lactation and serum prolactin level of sows on d 7 of lactation [206]. The total lactation yield was increased in sows when given 200 mg/kg soybean isoflavone and astragalus polysaccharide mixture in their diet during lactation [207]. Dietary supplementation with resveratrol at a dose of 300 mg/kg during gestation and lactation increased the lactose level in colostrum, and total solids and fat levels in 21-day milk [208]. In a study by Zhang et al. [209], dietary supplementation with micelle silymarin at doses of 0.5, 1, and 2 g/kg from d 109 of gestation to d 21 of lactation linearly increased the average daily milk yield during lactation and fat content in 14-d milk. These studies revealed that maternal dietary polyphenols could enhance the antioxidant capacity and immunity of the mammary glands, thereby improving the lactation performance of sows and ameliorating milk quality, and ultimately showing great potential to promote the growth and development, and health state of suckling piglets.

#### Fetal growth and development and placenta health

Evidence is growing that fetal growth and development and placenta health are improved when the diet of sows is supplemented with polyphenols during gestation. Meng et al. [203] reported that dietary supplementation with resveratrol at a dose of 300 mg/kg from d 20 of gestation to d 21 of lactation increased the activities and the mRNA expression levels of antioxidant enzymes in the placenta of sows by promoting the activation of the Sirt1/Nrf2 pathway. Supplementation with resveratrol at a dose of 300 mg/kg from d 75 of gestation and d 21 of lactation increased blood growth hormone (GH) and progesterone levels in sows [210]. The serum GH, type 1 insulin-like growth factor (IGF-1), and prolactin levels in sows were increased following supplementation with a mixture of soybean isoflavone and astragalus polysaccharide at a dose of 200 mg/kg during lactation [207]. Maternal dietary supplementation grape seed polyphenols at a dose of 300 mg/kg from d 80 of gestation to d 21 of lactation improved survival rate and preweaning survivability of piglets, increased the serum levels of progesterone and estradiol of sows, and reduced the number of dead fetuses [204]. Moreover, dietary supplementation with daidzein at a dose of 200 mg/kg from d 1 to 35 of gestation increased the number of viable embryos and the total number of embryos of sows, elevated the serum progesterone and estradiol-17β levels, and increased the concentrations of estrogen, progesterone, and IGF-1 in the amniotic fluid [211, 212]. Furthermore, daidzein supplementation also improved antioxidant capacity and reduced inflammation in ovarian tissue of sows by suppressing the TLR4/NF-κB signaling pathway and activating the Nrf2/HO-1 signaling pathway [212]. Li et al. [213] reported that the maternal supplementation with daidzein at a dose of 200 mg/kg during pregnancy increased the number of total born piglets and born alive piglets per litter, the serum estrogen and progesterone concentrations of sows and placental mRNA expression levels of sodium-coupled neutral amino acid transporter 1 and *IGF-1*. Dietary supplementation with hydroxytyrosol at a dose of 1.5 mg/kg in sows fed a diet rich in n-3 PUFA during d 35 to 100 of pregnancy improved the fatty acid compositions of the fetal muscle, such as by increasing ALA, EPA, DHA, and Σn-3 PUFA levels [214]. Similarly, during d 35 to 100 of gestation, maternal supplementation with hydroxytyrosol at a dose of 1.5 mg/kg increased the Σn-3 PUFA and Σn-6 PUFA levels in the LD muscle of IUGR fetus and improved fetal antioxidant status and glucose metabolism [215, 216]. In short, polyphenols play a crucial role in promoting fetal growth and development, as well as maintaining placental health status through various mechanisms. These include enhancing antioxidant capacity while reducing inflammation in ovarian tissue, boosting placental antioxidant capacity and nutrient transport, improving fetal metabolism, and regulating serum, amniotic fluid and placental levels of hormones associated with pregnancy, growth, and development in sows.

#### Growth and health status of suckling piglets

Suckling piglets mainly rely on mammary secretions for nutrients, antioxidants, and factors for growth and development. Dietary supplementation of sow diet with polyphenols during gestation and/or lactation has been shown to promote the growth and development of suckling piglets. The BW of suckling piglets on d 10 and 21 of lactation and the ADG on d 3 to 10 and 3 to 21 of lactation were linearly increased after supplementation of sow diet with soy isoflavones from d 90 of gestation to d 21 of lactation [217]. Dietary supplementation with resveratrol at a dose of 300 mg/kg from d 20 of gestation to d 21 of lactation increased litter weight and piglet weight at weaning and increased plasma CAT and GSH-Px levels in newborn piglets and weaning piglets [203]. Another study showed that supplementation of sow diet with resveratrol at a dose 300 mg/kg from d 20 of gestation to d 28 of lactation led to a higher ADG of suckling piglets during d 14 to 21 and 21 to 28 of lactation, as well as lower fecal scores during d 15 to 21 after birth and diarrhea during d 3 to 5 post-weaning [218]. They also found that maternal supplementation with resveratrol improved intestinal morphology, reduced intestinal inflammation, and raised the proportion of butyrate-producing bacteria, ultimately promoting the growth of piglets and alleviating diarrhea. Similarly, supplementation of the sow diet with resveratrol at a dose of 300 mg/kg from d 75 of gestation to d 21 of lactation increased the number of live births and litter weight at weaning, raised the relative abundances of *Lactobacillus* and *Alloprevotella*, but reduced the relative abundance of *Escherichia-shigella* in piglet feces [210]. Sun et al. [208] showed that supplementation of the sow diet with resveratrol at a dose of 300 mg/kg from d 20 of gestation to d 21 of lactation raised the plasma levels of high-density lipoprotein cholesterol, low-density lipoprotein cholesterol, insulin, and lipase of suckling piglets by increasing the enzyme activities and expression levels of genes associated with lipolysis, fatty acid uptake from circulating triacylglycerols, and lipogenesis in adipose tissue. A study conducted by Wang et al. [205] reported an increased litter birth weight, litter weaning weight, and litter gain, and reduced piglet mortality in sows fed a diet supplemented with garcinol at doses of 200 and 600 mg/kg during d 90 of gestation to d 21 of lactation. They also found that maternal dietary supplementation with garcinol at a dose of 600 mg/kg improved the acid-base balance of the blood of newborn piglets and increased the plasma IgA and IgG levels in piglets on d 14 of lactation. Additionally, supplementation of the sow diet with micelle silymarin at doses of 0.5, 1, and 2 g/kg increased the ADG of suckling piglets, litter weight, and litter weight gain at weaning, as well as the serum CAT and total antioxidant capacity levels from day 109 of gestation to d 21 of lactation [209]. Also, supplementation of a sow diet rich in n-3 PUFA with hydroxytyrosol at a dose of 1.5 mg/kg from d 35 of pregnancy to birth increased the ADG, BW, and growth rate of suckling piglets, and reduced lipidemic levels, leading to ameliorated fatty acid profiles in fat and muscle [219]. Overall, supplementation of the sow diet with polyphenols could effectively improve the growth performance and health status of suckling piglets, which may be achieved through alteration of the colostrum and milk quality of sows.

## Conclusions and perspectives

**Fig. 7:**
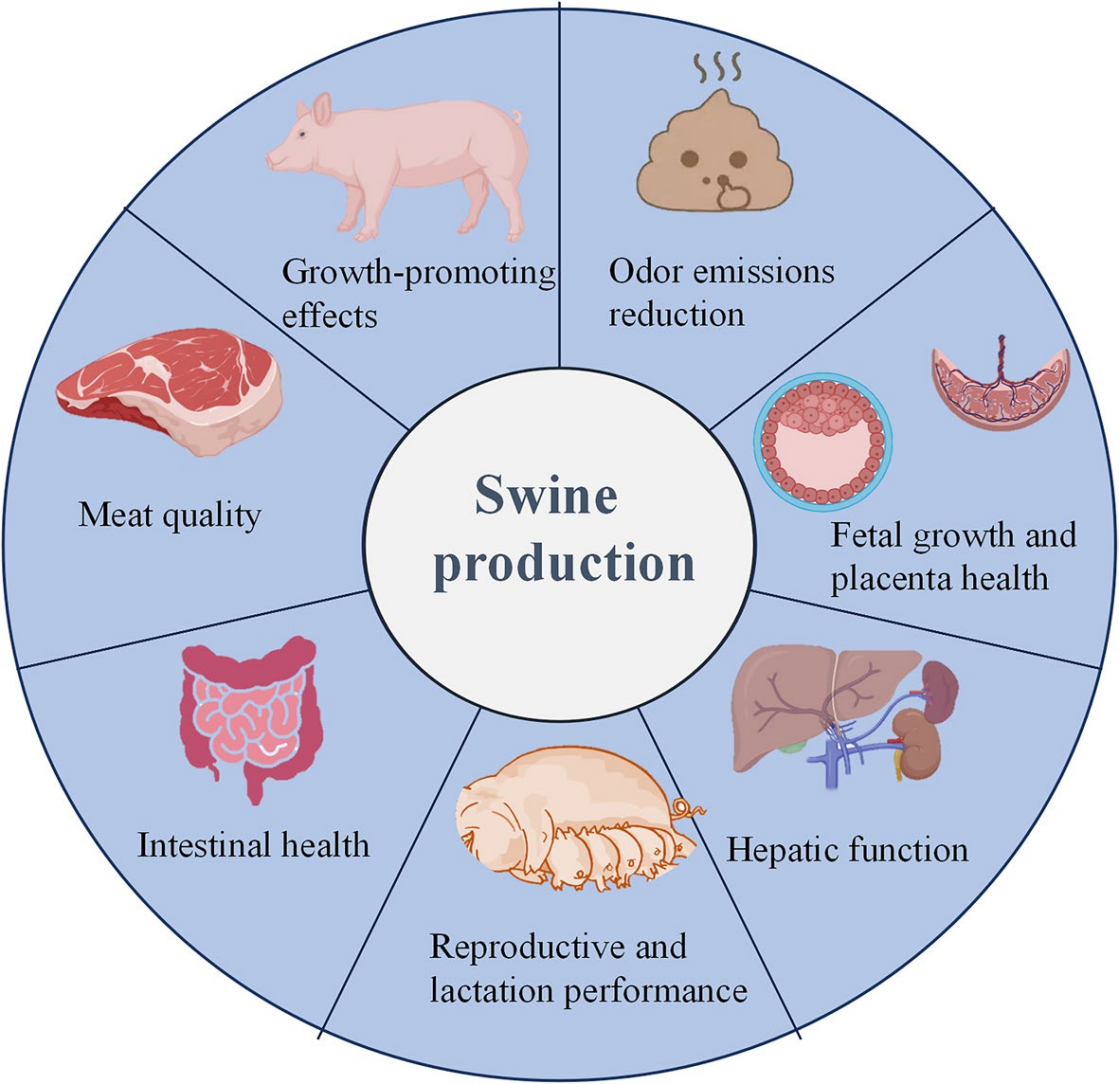
The summary of beneficial effects of dietary plant polyphenols in swine production. Dietary plant polyphenols could improve growth performance, meat quality, intestinal health, hepatic function, and odor emissions of swine, with ameliorated reproductive and lactation performance of sows and bolstered fetal growth and placenta health

Under the background of antibiotic resistance, polyphenols have recently received much attention as they are natural, non-toxic, environment-friendly, highly reproducible, and have a variety of physiological functions, such as antioxidant, anti-inflammatory, immunomodulatory, and metabolic regulatory functions. Numerous studies have reported that dietary supplementation with polyphenols improved the health status and growth performance of pigs, especially in piglets under adverse or stress conditions, and had conducive effects on the reproductive performance of sows. However, it should be noted that not all phenolic compounds were necessarily beneficial or effective in any case, and their physiological effects depended on a series of factors. Regarding the action of phenolic compounds, there were significant differences due to the inconsistency of sources, dosages, extraction method, dietary patterns, metabolism, feeding duration, and bioavailability of compounds, as well as the growth stage and health condition of pigs in some studies. Overall, this review highlighted the potential of using phenolic compounds as an alternative to antibiotics to improve meat and milk quality, intestinal health, hepatic function, odor pollution, and overall production performance of swine (as summarized in Fig. 7). Considering the current findings, various types of polyphenols warrant further research and application in swine production and provide more reference for the development and utilization of phenolic compounds.

Although natural plant polyphenols and associated feed additives have great potential in swine production, their research and development are still in the early stages. There are several challenges that need to be addressed in this field. Some of these challenges include the lack of systematic theoretical systems support, immature technology for extraction, separation, and processing, issues with product stability and quality control, the need for the establishment of the standard system, and precision nutrition technology. As such, future research into the development of plant polyphenols and other naturally active compounds should focus on the following specific aspects: (1) Promoting innovation in the basic theory and methods of natural plant bioactive substances by combining expertise from various disciplines, such as modern analytical chemistry, structural chemistry, biochemistry, animal physiology, animal nutrition, molecular biology, bioinformatics, and related fields. This involves understanding the relationship between the structure or dose of bioactive compounds found in plants and their effects on animals. It also includes studying their absorption and metabolic kinetics features, specific action targets, action pathways, and dynamic balance of supply and demand. These measures will help to optimize the systematic animal nutrition theory and comprehensibly achieve nutritional balance and precise nutrition of animals; (2) Supporting breakthroughs in extraction, purification, analysis, and identification processes for plant polyphenols. Using modern green revolution technology, such as synthetic biology, to modify the metabolic pathway of plants, thereby significantly enhancing the content of plant polyphenols. Taking advantage of the synthetic properties of microorganisms such as bacteria and microalgae, to increase productivity and reduce costs for polyphenols. These measures are anticipated to promote the industrialization and large-scale development of plant polyphenols and other natural bioactive compounds; (3) Establishing the high-throughput screening technology platform for plant polyphenols, accelerating the development and application of plant polyphenols by using an in vitro organoid culture system, building databases and quality standards, and expanding the sources for feed ingredients and feed additives; (4) Adjusting the supply amount of plant polyphenols and continuously optimizing the diet formulation based on the physiological conditions and growth stage of animals, while encouraging the priority of use of the polyphenols extracted from agricultural by-products as feed additives. These measures are expected to achieve efficient and healthy production of livestock and poultry breeding industry, reduce the production costs, and improve overall economic benefits; (5) Determining the measurement standards for plant polyphenols content, standardizing quantitative methods, and establishing a corresponding standard system for relevant platforms.

## Supplementary Information

**Additional file 1** Applications of polyphenols in swine production.

## Data Availability

Not applicable.

## Abbreviations

a*: Redness
ABCA1: ATP binding cassette subfamily A member 1
ACC: Acetyl-CoA carboxylase
ADFI: Average daily feed intake
ADG: Average daily gain
AhR: Aryl hydrocarbon receptor
AKT: Protein kinase B
ALA: α-Linolenic acid
AMPK: Adenosine-monophosphate-activated protein kinase
AP1: Activator protein 1
ASFV: African swine fever virus
b*: Yellowness
Bax: B-cell lymphoma-2-associated X protein
Bcl2: B-cell lymphoma-2
BW: Body weight
CAT: Catalase
CD: Crypt depth
COX-2: Cyclooxygenase-2
CPT1: Carnitine-palmitoyl transferase 1
DHA: Docosahexaenoic acid
DON: Deoxynivalenol
DSS: Dextran sulfate sodium
EPA: Eicosapentaenoic
ETEC: Enterotoxigenic *Escherichia coli*
F/G: Feed to gain ratio
GLUT2: Glucose transporter-2
GSH: Glutathione
GSH-Px: Glutathione peroxidase
H_2_O_2_: Hydrogen peroxide
HO-1: Heme oxygenase-1
HSL: Hormone-sensitive lipase
IgA: Immunoglobulin A
IGF-1: Type 1 insulin-like growth factor
IgG: Immunoglobulin G
IgM: Immunoglobulin M
IL-1β: Interleukin-1β
IL-6: Interleukin-6
IL-10: Interleukin-10
IMF: Intramuscular fat
IMP: Inosinic acid
iNOS: Inducible nitric oxide synthase
IPEC-1: Intestinal porcine epithelial cell line-J1
IPEC-J2: Intestinal porcine epithelial cell line-J2
IUGR: Intrauterine growth retardation
L*: Lightness
LD: *Longissimus dorsi*
LDH: Lactate dehydrogenase
LL: *Longissimus lumborum*
LPS: Lipopolysaccharide
LT: *Longissimus thoracis*
LXRα: Liver X receptorα
MAPK: Mitogen-activated protein kinases
MDA: Malondialdehyde
MDH: Malate dehydrogenase
MMPs: Matrix metalloproteinases
mTOR: Mammalian target of the rapamycin
MyHC: Myosin heavy chain
NF-κB: Nuclear factor-kappa B
NLRP3: Nod-like receptor pyrin domain containing 3
Nrf2: Nuclear factor erythroid 2-related factor 2
PEDV: Porcine epidemic diarrhea virus
PGC-1α: Proliferator-activated receptor γ coactivator‐1α
PI3K: Phosphatidylinositol 3-kinase
PPARα: Peroxisome proliferator-activated receptorα
PPARγ: Peroxisome proliferator-activated receptorγ
PRRSV: Porcine reproductive and respiratory syndrome virus
PRV: Pseudorabies virus
PUFA: Polyunsaturated fatty acid
ROS: Reactive oxygen species
SDH: Succinate dehydrogenase
SFA: Saturated fatty acid
SGLT1: Sodium-glucose transport protein 1
Sirt1: Sirtuin 1
SOD: Superoxide dismutase
TFEB: Transcription factor EB
TG: Triglyceride
TGEV: Transmissible gastroenteritis virus
TJ: Tight junction
TLR4: Toll-like receptor 4
TNF-α: Tumor necrosis factorα
T-SOD: Total superoxide dismutase
V/C: Villus height to crypt depth ratio
VH: Villus height
ZO-1: Zonula occludin-1
