# Supplementary material for: Natural plant polyphenols contribute to the ecological and healthy swine production
Source: J Anim Sci Biotechnol. 2024 Nov 4;15:146. doi: 10.1186/s40104-024-01096-3 (PMC11533317; doi:10.1186/s40104-024-01096-3)
Supplement: Supplementary file 1 — Additional file 1 Applications of polyphenols in swine production. [file 40104_2024_1096_MOESM1_ESM.docx]

**Additional files 1** Applications of polyphenols in swine production

| **Animals** | **Polyphenols** | **Stress model/**  **study design** | **Effects** | **Reference** |
| --- | --- | --- | --- | --- |
| Piglets  7-day-old | Puerarin | PEDV challenge  Dose 0.5 mg/kg BW  Duration: 12 days | ●raised BW and reduced morbidity of piglets  ●reduced AST, ALP, IL-6, IL-8, and TNF-α levels and the number of white blood cells and neutrophils in plasma  ●raised D-xylose level but lowered DAO level in plasma  ●raised the activities of T-SOD, GSH-Px, and CAT but lowered the levels of MPO and H_2_O_2_ in both the intestine and plasma  ●raised *Nrf2* mRNA level but lowered *HSP70* mRNA level in the ileum  ●increased the abundances of total eubacteria, *Enterococcus genus*, and *Lactobacillus genus* in the jejunum and cecum but reduced the abundance of *Clostridium coccoides* in the caecum | [1] |
| Piglets  7-day-old | Puerarin | ETEC challenge  Dose: 5 mg/kg  Duration: 10 days | ●no effects on ADG but raised ADFI  ●increased *Bifidobacterium* number in the colon and *Lactobacillus* number in the jejunum, cecum, and colon but decreased the *Escherichia coli* number in the jejunum and cecum  ●reduced MPO activity but increased the activities of CAT and SOD in the jejunum and ileum  ●lowered *IL-1β* mRNA level but raised *ICAM1*, *VCAM2*, *MX1*, *MX2*, and *GBP1* mRNA levels in the jejunum | [2] |
| Piglets  7-day-old | Ferulic acid | IUGR  Dose: 100 mg/kg  Duration: 21 days | ●reduced serum MDA concentration and serum and liver ROS concentrations  ●increased GSH-Px and T-SOD activities in serum and liver  ●decreased HDL-C and LDL-C levels in serum and increased TG and lipase levels in the liver  ●lowered *Keap1* gene expression but raised *SOD1*, *CAT*, *FAS*, *PPARα*, *LPL*, and *CD36* gene expressions in the liver | [3] |
| Piglets  7-day-old | Ferulic acid | IUGR  Dose: 1,000 mg/kg  Duration: 21 days | ●no effect on ADG but reduced F/G  ●reduced serum T-SOD and CAT concentrations  ●raised VH in the jejunum but reduced CD in the duodenum  ●elevated lactase and maltase activities in the duodenum and maltase activity in the jejunum  ●reduced cell apoptosis, cleaved caspase-3 and cleaved caspase-9 activities in the duodenum and ileum | [4] |
| Piglets  7-day-old | Tannic acid-chelated zinc | PEDV challenge  Dose: 50 mg/kg  Duration: 12 days | ●raised ADG and diminished diarrhea rate  ●raised duodenal and jejunal VH, V/C, and villus surface area but decreased duodenal, jejunal, and colonic CD  ●raised plasma CAT level and diminished plasma DAO, H_2_O_2_, and MDA levels  ●raised T-AOC and GSH-Px levels in duodenum, jejunum, and colon and T-SOD level in duodenum and jejunum but lowered MDA concentration in duodenum, jejunum, and colon | [5] |
| Piglets  7-day-old | Resveratrol | IUGR  Dose: 80 mg/kg  Duration: 14 days | ●no effect on BW  ●lowered PC, MDA, TG, free fatty acids, and glucose levels in the liver  ●elevated *SOD1*, *GSH-Px*, *PPARα*, *MTTP*, *CD36* and *LPL* mRNA abundances and lowered *Keap1* and *FATP-1* mRNA abundances in the liver | [6] |
| Weaned piglets  21-day-old | Baicalin | DON challenge  Dose: 1,000 mg/kg  Duration: 14 days | ●raised BW, ADG, and ADFI but lowered F/G  ●raised serum albumin level but lowered serum AST and ALP levels  ●lowered serum IL-8, IL-1β, IL-6, IFN-γ, and TNF-α levels and their mRNA levels in the jejunum and ileum  ●raised serum T-AOC, GSH, and GSH-Px levels but lowered serum MDA level  ●raised VH and V/C in the jejunum and ileum  ●raised mTOR protein concentration and mRNA level but lowered NF-κBp65 protein concentration and mRNA level in the jejunum and ileum | [7] |
| Weaned piglets  21-day-old | Puerarin | Diquat challenge  Dose: 1,000 mg/kg  Duration: 14 days | ●raised BW, ADG, and ADFI  ●raised VH in the jejunum and V/C in the jejunum and ileum  ●raised protein content and the ratio of RNA/DNA and protein/DNA in the ileum  ●raised proliferating cell nuclear antigen positive cells percentage in the jejunum and ileum  ●raised *Occludin* gene expression in jejunum and ileum  ●raised SOD, GSH-Px, T-AOC levels in the jejunum and GSH-Px and T-AOC levels in the ileum  ●raised Nrf2, HO-1, GCLC, and GCLM protein expressions in the jejunum | [8] |
| Weaned piglets  25-day-old | *Eucommia ulmoides* Flavones | Dose: 100 mg/kg  Duration: 35 days | ●raised BW and ADG but diminished F/G and diarrhea index  ●increased V/C in the jejunum and VH in the jejunum and ileum  ●raised ileal *Lactobacillus* number on day 15 but decreased ileal *Escherichia coli* number on days 15 and 35  ●higher serum total protein and IgG levels on day 15 | [9] |
| Weaned piglets  23-day-old | Soybean isoflavones | Dose: 550 mg/kg  Duration: 72 days | ●higher BW on day 72 and ADG and ADFI from days 42 to 72  ●higher VH and V/C in the jejunum  ●increased plasma SOD activity on days 28 and 42 and reduced MDA content in plasma and jejunum on day 42 | [10] |
| Weaned piglets  23-day-old | Daidzein | Dose: 50 mg/kg  Duration: 72 days | ●raised BW and ADG  ●increased plasma SOD activity on day 42 and decreased plasma MDA content on day 14 | [11] |
| Weaned piglets  21-day-old | Embelin | Dose: 400 and 600 mg/kg  Duration: 28 days | ●raised BW and ADG but lowered F/G and diarrhea rate  ●raised *IL-10* mRNA abundance but lowered *IL-1β* and *NF-κB* mRNA abundances in the jejunum and ileum  ●raised VH in the jejunum and V/C in the jejunum and ileum  ●lowered serum D-lactate and DAO levels  ●raised ZO-1 and Claudin-1 protein abundances but decreased PCAF and NF-κB activities in the jejunum and ileum | [12] |
| Weaned piglets  28-day-old | Emodin | LPS challenge  Dose: 300 mg/kg  Duration: 21 days | ●no effects on BW, ADG, ADFI, and F/G  ●raised V/C in the jejunum but lowered plasma DAO and D-lactate levels  ●raised CAT and GSH-Px levels but diminished MDA level in the jejunum  ●elevated *GPX1*, *SOD2*, *CAT*, and *IL-10* mRNA abundances but lowered *IL-1β*, *IL-6*, and *TNF-α* mRNA abundances in the jejunum  ●lifted the protein and mRNA abundances of Occludin, Claudin-1, and ZO-1 in the jejunum  ●lowered the protein abundances of P-NF-κB, P-IκBα, and COX-2 in the jejunum  ●raised the abundances of *Ruminococcus_1* and *Ruminococcaceae_UCG-014* and the concentrations of isobutyric acid, valeric acid, isovalerate, and acetic acid in the cecum | [13] |
| Weaned piglets  21-day-old | Quercetin | Dose: 1,000 mg/kg  Duration: 14 days | ●lowered fecal scores on days 7 and 14  ●raised T-AOC, CAT, and GSH/GSSG levels but lowered MDA level in the jejunum  ●raised Claudin-1 protein expression and improved VH and V/C but lowered CD and cell apoptosis in the jejunum  ●increased the relative abundances of Fibrobacteres, *Akkermansia muciniphila*, *Clostridium butyricum*, *Clostridium celatum*, and *Prevotella copri* but decreased the relative abundances of Proteobacteria, *Lactobacillus coleohominis*, and *Ruminococcus bromii* in faeces  ●raised propionic acid and butyric acid contents in the jejunum and colon | [14] |
| Weaned piglets  6.8 kg | Quercetin | DON challenge  Dose: 1,000 mg/kg  Duration: 14 days | ●mitigated impaired villus-crypt morphology, such as villus atrophy and fusion  ●raised the protein expressions of Claudin-4, Occludin, and Nrf2 in the duodenum | [15] |
| Weaned piglets  28-day-old | Proanthocyanidin | Dose: 30, 300, 600 and 1,200 mg/kg  Duration: 42 days | ●no effects on BW, ADG, and ADFI, but lowered F/G and diarrhea rate  ●increased the apparent digestibility of dry matter, ether extract, gross energy, and ash  ●elevated VH and V/C in the duodenum and jejunum and decreased CD in the duodenum  ●elevated the mRNA levels for duodenal *MUC1*, *MUC2*, and *FATP-1* and jejunal and ileal *FATP-4* | [16] |
| Weaned piglets  28-day-old | Grape seed procyanidins | Dose: 40, 70, and 100 mg/kg  Duration: 28 days | ●dietary 40 mg/kg gape seed procyanidins raised ADG but lowered F/G and diarrhea rate  ●dietary 40 and 70 mg/kg gape seed procyanidins raised amylase and lipase activities in the jejunum, T-SOD, and GSH-Px levels in the liver and LD muscle, and the mRNA levels for *SOD*, *GSH-Px*, and *CAT* in the liver  ●dietary 70 mg/kg gape seed procyanidins raised serum CAT, T-AOC, GSH-Px, and T-SOD levels and lowered serum MDA level; dietary 40 mg/kg gape seed procyanidins lowered MDA level in the liver and LD muscle | [17] |
| Weaned piglets  21-day-old | Grape seed procyanidins | Dose: 100 mg/kg  Duration: 28 days | ●raised SOD concentration and *SOD* and *CAT* mRNA expressions but lowered MDA concentration in the jejunum  ●elevated the richness of the bacterial community in the cecum and colon and the abundances of probiotic *Prevotellaceae_NK3B31*_group and *Prevotella_1* in the colon and rectum  ●increased the concentrations of acetic acid and butyric acid in the cecum and colon | [18] |
| Weaned piglets  21-day-old | Grape seed procyanidins | Dose: 100 mg/kg  Duration: 28 days | ●raised VH and V/C in the duodenum, jejunum, and ileum  ●raised the expression of genes of encoding EGFR, IGF-1, and IGF-1 receptor in the duodenum | [19] |
| Weaned piglets  21-day-old | Chlorogenic acid | Dose: 250, 500, and 1,000 mg/kg  Duration: 28 days | ●linearly raised ADG but linearly reduced F/G  ●increased VH and V/C and decreased CD in the duodenum  ●increased GSH-Px level in serum and duodenum but lowered MDA level in serum, jejunum, and ileum  ●increased *Occludin* gene expression in the duodenum, jejunum, and ileum  ●increased *Bacillus* and *Bifidobacterium* populations in the cecum and *Lactobacillus* population in the colon but decreased *Escherichia coli* population in the colon  ●increased butyric acid content in the cecum and propionic acid, butyric acid, and total SCFA contents in the colon. | [20] |
| Weaned piglets  24-day-old | Chlorogenic acid | Dose: 1,000 mg/kg  Duration: 14 days | ●raised ADG but lowered F/G and diarrhea incidence  ●increased the apparent digestibility of crude protein, crude fat, and ash  ●increased serum albumin, IGF-1, SOD, GSH-Px, and CAT levels but decreased serum urea nitrogen and MDA levels  ●increased sucrase, lactase, maltase, and alkaline phosphatase activities in the jejunum  ●raised the mRNA abundances for *SGLT1* and *ZNT1* in the duodenum and *SGLT1*, *GLUT2*, and *DMT1* in the jejunum | [21] |
| Weaned piglets  24-day-old | Chlorogenic acid | Dose: 1,000 mg/kg  Duration: 14 days | ●increased serum IgG and IL-10 contents but decreased serum IL-1β and TNF-α contents  ●increased SIgA content in the jejunum and ileum  ●increased VH, villus width, and V/C in the duodenum and jejunum  ●decreased the gene expressions of *caspase-3* and *caspase-9*, and the Bax/Bcl2 ratio but increased the *Bcl2* gene expression in the duodenum and jejunum | [22] |
| Weaned piglets  24-day-old | Chlorogenic acid | Dose: 1,000 mg/kg  Duration: 14 days | ●raised the length of the small intestine and enhanced DAO activity in the jejunum and ileum  ●raised acetate concentration in the ileum and cecum and improved propionate and butyrate concentrations in the cecum but decreased pH value in the ileum and cecum  ●increased the total 16S rRNA gene copies and alpha diversity, and the abundances of Firmicutes, Bacteroidetes, *Lactobacillus* spp., *Prevotella* spp., *Anaerovibrio* spp., and *Alloprevotella* spp. in the cecum, whereas decreased proteobacteria abundance | [23] |
| Weaned piglets  24-day-old | Chlorogenic acid | Dose: 1,000 mg/kg  Duration: 14 days | ●lower serum DAO, D-Lactate, and endotoxin levels  ●decreased histamine and MDA contents in the jejunum and ileum  ●increased the activities of GSH-Px and CAT and the gene expressions of *Nrf2* and *HO-1* in the duodenum and jejunum  ●raised the distribution of Claudin-1 in the duodenum, jejunum, and ileum, improved the gene expressions of *ZO-1* and *Claudin-1* in the jejunum and ileum, and *Occludin* in the jejunum  ●decreased the gene expressions of *IL-1β*, *TNF-α*, *TLR4*, and *NF-κB* in the jejunum and *IL-6* and *TNF-α* in the ileum | [24] |
| Weaned piglets  21-day-old | Chlorogenic acid | Diquat challenge  Dose: 1,000 mg/kg  Duration: 21 days | ●higher ADG and ADFI  ●decreased serum DAO, D-lactate, and MDA levels but increased serum SOD and GSH-Px levels  ●increased VH and the activity of alkaline phosphatase in the jejunum and ileum  ●increased the mRNA levels for *SGLT1* in the duodenum and *GLUT2* in the jejunum  ●enhanced the activities of sucrase, lactase, and maltase and the mRNA levels of *Nrf2* and *HO-1* but decreased the mRNA levels of *Bax*, *caspase-3*, and *caspase-9*, and the ratio of Bax/Bcl2 in the duodenum and jejunum  ●increased the mRNA levels for *Occludin* and *Claudin-1* in the duodenum, *Occludin*, *ZO-1*, and *Claudin-1* in the jejunum, and *ZO-1* and *Claudin-1* in the ileum | [25] |
| Weaned piglets  21-day-old | Chlorogenic acid | Diquat challenge  Dose: 1,000 mg/kg  Duration: 21 days | ●higher BW  ●raised VH in the jejunum and decreased cell apoptosis  ●decreased MDA level and mRNA levels for *IL-1β* and *TNF-α*, but increased GSH-Px and CAT levels in the jejunum | [26] |
| Weaned piglets  21-day-old | Caffeic acid | Dose: 250 and 500 mg/kg  Duration: 28 days | ●increased acetate acid, propionate acid, and total SCFA contents in the colon  ●increased *Bifidobacterium* and *Lactobacillus* populations and decreased *Escherichia coli* population in the colon  ●the mRNA levels of *Claudin-1*, *ZO-1*, *Occludin*, and *MUC2* in the colon were increased when diets containing 250 mg/kg caffeic acid  ●supplementation of caffeic acid at 500 mg/kg decreased the mRNA levels of *IL-1β*, *IL-6*, and *TNF-α* in the colon | [27] |
| Weaned piglets  7.43 kg | Caffeic acid | LPS challenge  Dose: 500 mg/kg  Duration: 28 days | ●increased BW on days 21 and 28 and ADG from days 1 to 21 in non-LPS-challenged piglets  ●increased BW on day 28 and ADG from days 21 to 28 in LPS-challenged piglets  ●increased serum T-AOC, CAT, SOD, and GSH-Px levels but decreased serum D-lactate, DAO, IL-1β, IL-6, TNF-α, MDA, and H_2_O_2_ levels  ●increased *Occludin*, *Claudin-1*, and *ZO-1* gene expressions but decreased *NF-κB*, *IL-6*, and *IL-1β* gene expressions in the colon  ●decreased *Bax*, *Fas* gene expressions and the ratio of Bax/Bcl2 but increased *Bcl2* gene expression in the colon  ●increased the alpha diversity and *Alloprevotella* and *Prevotellaceae_UCG-001* abundances but decreased *Rikenellaceae_RC9_gut_*group abundance in the colon  ●improved metabolic disorders of colonic BAs and SCFAs in LPS-challenged piglets, such as improved primary BAs and isovalerate contents. | [28] |
| Weaned piglets  21-day-old | Ellagic acid and berberine | Dose: 10 mg/kg  Duration: 14 days | ●raised ADG and ADFI but diminished fecal score  ●increased VH and V/C but diminished cell apoptotic in the jejunum  ●raised T-AOC and CAT levels but lowered MDA and GSSG levels in the jejunum  ●raised the relative mRNA abundances of *Occludin*, *Claudin-1*, and *ZO-1* in the jejunum  ●raised alpha diversity in the colon, the abundances of Firmicutes, *Lactobacillus*, and *phascolarctobacterium* in feces, and lowered the abundances of Bacteroidetes and *parabacteroides* in feces  ●increased the contents of propionate and butyrate in the jejunum and colon | [29] |
| Weaned piglets  30-day-old | Ellagic acid | Dose: 500 mg/kg  Duration: 40 days | ●raised ADG but decreased diarrhea rate  ●reduced serum DAO concentration  ●downregulated the gene expression related to immune response pathways in the jejunum  ●reduced *Prevotella_9* abundance in the cecum but increased *Lactobacillus delbrueckii* abundance in the cecum and *Lactobacillus reuteri* abundance in the rectum | [30] |
| Weaned piglets  23-day-old | Ellagic acid | Dose: 1,000 mg/kg  Duration: 14 days | ●increased ADG and decreased fecal scores  ●increased Occludin protein expression in the jejunum, ●increased T-AOC, CAT, and GSH/GSSG levels but decreased MDA level in the jejunum  ●increased VH and V/C but decreased cell apoptosis in the jejunum  ●increased the diversity of microbiota and the abundances of Ruminococcaceae and *Clostridium ramosum* in feces  ●increased acetic acid, propionic acid, butyric acid, and total SCFA contents in the jejunum and colon | [31] |
| Weaned piglets  21-day-old | Ellagic acid | Paraquat challenge  Dose: 50, 100, and 200 mg/kg  Duration: 21 days | ●no effects on BW, ADG, ADFI, and F/G  ●increased serum SOD level but decreased serum MDA and DAO levels  ●increased Nrf2, NQO1, HO-1, Claudin-3, and Occludin protein expressions in the jejunum and ileum.  ●raised VH, V/C, and goblet cell number but decreased CD in the jejunum and ileum | [32] |
| Weaned piglets  28-day-old | Ellagic acid | Paraquat challenge  Dose: 50, 100, and 200 mg/kg  Duration: 21 days | ●mitigated paraquat-induced hepatic fibrosis, steatosis, and high apoptotic rate and reduced serum ALT and AST levels  ●raised serum IL-4 and IL-13 levels, decreased serum IL-6 and TNF-α levels, ameliorated inflammatory cell infiltration into hepatic tissue, and lowered hepatic P-NF-κB protein abundance  ●raised SOD and GSH-Px activities and HO-1 and P-Nrf2 protein abundances in the liver  ●increased the abundances of *Lactobacillus reuteri* and *Lactobacillus amylovorus* in the cecum | [33] |
| Weaned piglets  21-day-old | Ferulic acid | Dose: 500 and 4,500 mg/kg  Duration: 35 days | ●no effects on BW, ADG, ADFI, and F/G  ●increased the protein expression and proportion of slow MyHC fiber and the gene expressions of *MyHC Ⅰ* and *MyHC IIa*, but decreased the protein expression and proportion of fast MyHC fiber and the gene expression of MyHC IIb in the LD muscle  ●elevated MDH and SDH activities  ●elevated the gene expressions of *AMPKα1*, *AMPKα2*, *TNNI1*, *PGC-1α*, *Sirt1*, *MEF2C* and the protein expressions of TNNI1, PGC-1α, Sirt1, MEF2C, P-AMPK in the LD muscle  ●lifted the gene expressions of *ATP5G*, *COX1*, *NRF1*, *TFAM*, *TFB1M*, and *Cytc* in the LD muscle and liver | [34] |
| Weaned piglets  21-day-old | Ferulic acid | Dose: 500 and 4,500 mg/kg  Duration: 35 days | ●increased CAT activity, the mRNA levels of *Nrf2*, *SOD*, *GST*, and *GR*, and the protein expressions of Nrf2 and NQO1 in the jejunum  ●dietary 500 mg/kg ferulic acid increased ZO-1 protein expression and the mRNA levels of *ZO-1*, *ZO-2*, *MUC1*, *MUC2*, *Occludin*, and *Claudin-1* in the jejunum | [35] |
| Weaned piglets  21-day-old | Ferulic acid | Dose: 500 and 4,500 mg/kg  Duration: 5 weeks | ●decreased MDA level but increased T-SOD activity in serum, LD muscle, and liver  ●increased the mRNA levels of *SOD1*, *SOD2*, *CAT*, *GST*, *GPX1*, and *GR* and the protein levels of Nrf2, NOQ1, and HO-1 in the LD muscle and liver  ●lowered TC and LDL-C levels in serum, TC level in the LD muscle, and TG level in the liver but raised HDL-C level in serum  ●raised the mRNA levels for *HSL*, *CPT1*, and *PPARα* in the liver | [36] |
| Weaned piglets  28-day-old | Ferulic acid or vanillic acid | LPS challenge  Dose: 4,000 mg/kg  Duration: 21 days | ●supplementation of vanillic acid raised BW and ADG  ●decreased the serum levels of IL-1β, IL-2, IL-6, TNF-α, and TBARS  ●raised *Occludin* gene expression in the ileum  ●increased the Firmicutes/Bacteroidetes ratio, reduced the abundances of the Prevotellaceae family, including *Prevotella 9* and *Prevotella 2* genera, and enriched the Lachoiraceaea family including the *Lachnospiraceae FCS020* group in the cecum  ●dietary vanillic acid reduced the abundance of *Prevotella 7* and *Prevotella 1* but enriched the abundance of *Lachnospira, Eubacterium eligens* group and *Eubacterium xylanophilum* group in the cecum | [37] |
| Weaned piglets  24-day-old | Gallic acid | Dose: 400 mg/kg  Duration: 28 days | ●raised BW and ADG regardless of high or low weaning weight  ●lowered diarrhea incidence, more particularly in low weaning weight piglets  ●reduced plasma MDA level in low weaning weight piglets | [38] |
| Weaned piglets  25-day-old | Gallic acid | Dose: 100, 200, and 400 mg/kg  Duration: 21 days | ●no effects on ADG, ADFI, and F/G but lowered diarrhea incidence  ●increased VH in the jejunum and raised V/C in the jejunum and ileum but decreased CD in the jejunum  ●increased SIgA content but decreased *TNF-α* and *NF-κB* gene expressions in the ileum | [39] |
| Weaned piglets  21-day-old | Protocatechuic acid | LPS challenge  Dose: 4,000 mg/kg  Duration: 21 days | ●increased ADG  ●decreased the levels of serum IL-1β, TNF-α, TBARS, IgM, and C-reactive protein | [40] |
| Weaned piglets  28-day-old | Protocatechuic acid | LPS challenge  Dose: 4,000 mg/kg  Duration: 21 days | ●no effects on BW, ADG, and ADFI but decreased F/G  ●lowered serum TBARS, IL-6, IL-2, and TNF-α levels  ●increased ZO-1 and Claudin-1 gene and protein expressions in the ileum  ●increased Firmicutes/Bacteroidetes ratio in the cecum, reduced the abundances of *Prevotella 9*, *Prevotella 2*, *Holdemanella*, and *Ruminococcus torques* group at the genus level, and increased the abundances of *Roseburia*, and *Desulfovibrio* at the genus level | [41] |
| Weaned piglets  21-day-old | Tannin | Dose: 1,000 mg/kg  Duration: 14 days | ●increased GSH-Px activity, ZO-1 and Keap1 protein expressions in the jejunum but reduced serum MDA level | [42] |
| Weaned piglets  21-day-old | Tannin | Dose: 2,000 mg/kg  Duration: 28 days | ●no effects on BW, ADG, ADFI, and F/G but linearly reduced diarrhea rate and diarrhea index  ●raised VH in the duodenum and jejunum and enhanced V/C in the ileum  ●lifted *Bacillus* counts in the cecum and colon  ●raised acetic acid, propionic acid, butyric acid, isovaleric acid, and total SCFA levels in the colon  ●raised the mRNA levels for *ZO-1*, *ZO-2*, and *Claudin-2* in the jejunum | [43] |
| Weaned piglets  21-day-old | Tannin | Dose: 2 and 10 g/kg  Duration: 28 days | ●no effects on BW, ADG, and F/G but linearly reduced diarrhea rate, index, and score  ●lowered serum DAO and D-lactate concentrations  ●raised V/C but lowered CD in the duodenum  ●elevated Occludin protein distribution in the duodenum, jejunum, and ileum  ●raised *ZO-2* and *Occludin* mRNA abundances in the duodenum and *ZO-1* mRNA abundance in the ileum | [44] |
| Weaned piglets  28-day-old | Coated tannin | Dose: 1,500 mg/kg  Duration: 20 days | ●increased ADG and ADFI but lowered F/G and diarrhea incidence  ●enhanced crude protein apparent digestibility and jejunal maltase and sucrase activities.  ●improved intestinal morphology, which was indicated by the intestinal villi and microvilli arranged more densely  ●raised ZO-1 and Claudin-1 protein expressions in the colon and lowered serum DAO concentration  ●augmented colonic Ruminococcaceae and Megasphaera abundances  ●enhanced the abundance of pathways related to butyrate metabolism and tryptophan metabolism and decreased the function of lipopolysaccharide biosynthesis proteins. | [45] |
| Weaned piglets  28-day-old | Hydrolysable tannins | Dose: 1,000 mg/kg  Duration: 28 days | ●raised ADG but reduced diarrhea rate  ●elevated trypsin and lipase activities in the jejunum  ●lifted jejunal VH and ileal V/C  ●raised propionic acid, butyric acid, and acetic acid concentrations in the colon | [46] |
| Weaned piglets  21-day-old | Magnolol | Dose: 400 mg/kg  Duration: 35 days | ●increased crude protein apparent digestibility  ●decreased the contents of urea, creatinine, and TG in serum  ●increased the numbers of *Lactobacillus*, *Bifidobacterium*, and *Ruminococcus* but decreased the *Escherichia coli* number in feces  ●decreased the fecal pH value and the contents of phenol, p-cresol, skatole, putrescine, cadaverine, and total biogenic amine  ●increased the fecal contents of acetate and SCFA | [47] |
| Weaned piglets  28-day-old | Resveratrol | Dose: 150 and 300 mg/kg  Duration: 42 days | ●no effects on BW, ADG, ADFI, and F/G  ●increased the gene and protein expressions of slow MyHC I, the activities of SDH and MDH, and the proportion of type I fiber, but decreased the activity of LDH and the proportion of type II fiber in the LD muscles  ●raised the mRNA and protein abundances of Sirt1 and PGC-1α and the protein abundance of P-AMPK in the LD muscles | [48] |
| Weaned piglets  28-day-old | Resveratrol | Dose: 150 and 300 mg/kg  Duration: 42 days | ●raised IgG and GSH-Px levels in serum and T-AOC and T-SOD levels in the liver but decreased MDA level in serum and liver  ●raised VH and V/C but lowered CD in the jejunum  ●increased the mRNA expressions of *IL-10* and *ZO-1* in the jejunum | [49] |
| Weaned piglets  35-day-old | Resveratrol | PRV challenge  Dose: 10, 30, and 90 mg/kg BW  Duration: 21 days | ●raised BW but reduced the mortality  ●elevated the serum concentrations of IFN-α, IFN-γ, TNF-α, and IL-12  ●relieved the inflammation in the brain | [50] |
| Weaned piglets  35-day-old | Resveratrol | OSO challenge  Dose: 300 mg/kg  Duration: 28 days | ●no effects on BW, ADG, ADFI, and F/G  ●raised trypsin, sucrase, lipase, and α-amylase activities in the jejunum  ●increased VH and V/C in the jejunum  ●raised *Occludin* gene level in the jejunum and ileum and improved Occludin protein distribution in the ileum but lowered plasma DAO and D-lactate levels  ●raised *SOD2* gene level in the jejunum and colon and *GPX1* and *IL-10* gene levels in the colon but lowered *TNF-α* gene level in the colon and *TNF-α* and *NF-κB* gene levels in the jejunum  ●raised Firmicutes abundance and the concentrations of acetic acid and butyrate but lowered the abundances of Bacteroidetes, *Prevotella_1*, *Clostridium_sensu_stricto_6*, and *Prevotellaceae_UCG003* in the colon | [51] |
| Weaned piglets  21-day-old | Resveratrol | IUGR  Dose: 300 mg/kg  Duration: 129 days | ●raised a* value and lowered b* value in the LL muscle and decreased drip loss at 24 h and 48 h, especially in the normal weight group  ●lowered PC and MDA concentrations but raised T-SOD, GSH-Px concentrations in plasma and LL muscle  ●lowered TG and free fatty acids levels but raised LPL level in the LL muscle  ●raised MyHC I *gene* expression in the LL muscle  ●raised the gene and protein expression for PPARα and the gene expressions for *SOD1*, *GCLM*, and *CPT1* but lowered the gene expressions for *SREBP1* and *SCD1* in the LL muscle | [52] |
| Weaned piglets  35-day-old | Resveratrol | Diquat challenge  Dose: 100 mg/kg  Duration: 14 days | ●raised ADG and ADFI  ●lifted T-AOC level but lowered H_2_O_2_ and MDA levels in the jejunum  ●raised Claudin-1, Occludin, and ZO-1 protein expression and TER value but lowered FD4 flux in the jejunum  ●ameliorated mitochondria swelling, vacuolation, and cracked cristae  ●raised mitochondrial membrane potential, the activities of mitochondrial complexes Ⅰ, Ⅱ, Ⅲ, and Ⅳ, and mitochondrial DNA content but lowered ROS production in the jejunum  ●elevated the protein expressions of PINK1, Parkin, and LC3-Ⅱ and the ratio of LC3-Ⅱ/LC3-Ⅰ in the jejunum | [53] |
| Weaned piglets  28-day-old | Resveratrol | Diquat challenge  Dose: 30 and 90 mg/kg  Duration: 21 days | ●increased VH and V/C but decreased CD in the jejunum  ●lowered plasma D-lactate and DAO concentrations  ●elevated the mRNA expressions of *Occludin*, *Claudin-1*, *ZO-1*, and *IL-10* but decreased *TNF-α* mRNA expression in the jejunum  ●raised SOD, GSH-Px, and CAT activities but diminished MDA content in the jejunum  ●increased the mRNA expressions of *SOD1*, *SOD2*, *CAT*, *GPX1*, *HO-1*, *Nrf2*, *NQO1*, *AhR*, and *CYP1A1* in the jejunum | [54] |
| Weaned piglets  28-day-old | Resveratrol | Diquat challenge  Dose: 30 and 90 mg/kg  Duration: 21 days | ●raised ADG and ADFI but decreased F/G and fecal score  ●decreased stress indicators, such as serum adrenocorticotropic hormone and cortisol levels  ●increased serum IgA, IL-4, IgM, SOD, GSH-Px, and CAT levels but lowered serum MDA, TNF-α, IL-6, and IL-1β levels | [55] |
| Weaned piglets  28-day-old | Resveratrol | Diquat challenge  Dose: 90 mg/kg  Duration: 22 days | ●lowered the relative abundances of Firmicutes and Actinobacteria, *Ruminococcaceae UCG-005*, and *Eubacterium coprostanoligenes*, but raised the relative abundances of *Clostridium sensu stricto 1* and *Lachnospiraceae unclassified* in the colon  ●improved the relative abundances of indole-3-carbinol, 5-hydroxyindole-3-acetic acid, indole, and uridine in the colon | [56] |
| Weaned piglets  28-day-old | Resveratrol | DON challenge  Dose: 300 mg/kg  Duration: 28 days | ●raised BW, ADG, and ADFI  ●increased VH and V/C in the jejunum  ●lowered plasma DAO and D-lactate levels  ●elevated SOD and T-AOC levels but lowered MDA level in the jejunum  ●raised mitochondrial membrane potential but lowered ROS production in the jejunum | [57] |
| Weaned piglets  21-day-old | Resveratrol | DON challenge  Dose: 300 mg/kg  Duration: 28 days | ●raised BW and ADG but lowered F/G  ●increased VH, V/C, and goblet cells in the jejunum  ●decreased plasma D-lactate concentration  ●raised *Occludin* and *Claudin-1* gene levels and Occludin, ZO-1, and Claudin-1 protein levels in the jejunum  ●reduced apoptosis cell numbers, caspase3 and Bax protein levels but raised Bcl2 protein level in the jejunum  ●reduced TNF-α, IL-1β, and IL-6 levels, and their gene levels in the jejunum  ●raised T-SOD and T-AOC level, *SOD1*, *GCLC*, *GCLM*, *HO-1*, and *NQO1* gene levels, and SOD1 and GCLC protein levels in the jejunum  ●increased butyrate concentration and the abundances of *Lactobacillus* and *Roseburia* but lowered the abundances of *Bacteroides* and *unidentified Enterobacteriaceae* in the colon | [58] |
| Weaned piglets  21-day-old | Resveratrol | DON challenge  Dose: 300 mg/kg  Duration: 28 days | ●raised T-AOC and GSH levels but lowered MDA level in plasma and ileum  ●increased the gene expressions of *SOD1*, *NQO1*, and *GCLM* but decreased the contents and gene expressions of IL-1β, IL-6, and TNF-α in the ileum  ●increased porcine β defensin 2, protegrin 1-5, and *MUC2* gene expressions and MUC2 and SIgA protein expressions in the ileum  ●elevated the abundances of class *Bacilli*, order *Lactobacillales*, family *Lactobacillaceae*, and species *Lactobacillus gasseri* in the colon | [59] |
| Weaned piglets  28-day-old | Resveratrol  (dry suspension) | Rotavirus challenge  Dose: 3, 10, and 30 mg/kg  Duration: 53 days | ●decreased MDA content in serum and liver, increased T-SOD and GSH-Px activities in serum and liver | [60] |
| Weaned piglets  28-day-old | Resveratrol  (dry suspension) | Dose: 100, 330, and 1,000 mg/kg  Duration: 14 days | ●no impacts on BW, ADG, ADFI, and F/G  ●raised peripheral blood lymphocyte and splenic lymphocyte percentages, and elevated IgG, IgM, and IgA levels in serum  ●raised serum T-AOC and IFN-γ levels but lowered serum TNF-α and IL-12 levels | [61] |
| Weaned piglets  21-day-old | Pterostilbene | Dose: 300 mg/kg  Duration: 7 days | ●reduced the number of apoptotic cells and the levels of superoxide anion and 8-OHdG in the liver  ●increased mitochondrial deoxyribonucleic acid content, the activities of citrate synthase, Sirt1, complexes I and III, and ATP synthase, and the protein expression of glucose-regulated protein 78, activating transcription factor 6, and phosphorylation of inositol-requiring enzyme 1 α | [62] |
| Weaned piglets  6.30 kg | Pterostilbene | LPS challenge  Dose: 300 mg/kg  Duration: 14 days | ●decreased plasma ALT and AST levels, reduced massive infiltration of neutrophils and inflammatory macrophages in the liver  ●reduced the contents of MDA, 8-OHdG, GSSG, MPO, the mRNA levels of *IL-1β*, *IL-6*, and *TNF-α*, and MPO protein expression in the liver  ●diminished hepatic apoptosis and caspase-3 activity  ●suppressed the PP2A/NF-κB/NLRP3 signaling pathway | [63] |
| Weaned piglets  21-day-old | Pterostilbene | Diquat challenge  Dose: 300 mg/kg  Duration: 15 days | ●raised BW  ●decreased plasma AST and ALT levels and hepatic caspase-3 and caspase-9 levels  ●reduced hepatic apoptosis  ●raised Nrf2 mRNA and protein abundance as well as Sirt1 activity and protein abundance but lowered the productions of TC, superoxide anion, and MDA in the liver  ●inhibited mitochondrial swelling, membrane potential collapse, and adenosine triphosphate depletion | [64] |
| Weaned piglets  21-day-old | Pterostilbene | Diquat challenge  Dose: 300 mg/kg  Duration: 14 days | ●reduced MDA, PC, and 8-OHdG contents but raised T-SOD and GSH-Px activities in the jejunum  ●lifted VH and V/C in the jejunum but decreased jejunal apoptosis rate  ●decreased plasma DAO and D-lactate levels but increased ZO-1 and Occludin protein expression in the jejunum  ●reduced the production of mitochondrial superoxide anions but raised SOD2 activity in the jejunum  ●elevated the activities of mitochondrial complex I, complex III, complex IV, and ATP synthase and the contents of ATP and mitochondrial DNA in the jejunum  ●raised Sirt1 activity and Sirt3 and TFAM protein expressions but lowered PGC-1α acetylation protein expression in the jejunum | [65] |
| Weaned piglets  21-day-old | Resveratrol or pterostilbene | Dose: 300 mg/kg  Duration: 7 days | ●no effects on ADG and ADFI; only pterostilbene supplementation lowered F/G  ●increased VH but reduced apoptotic cell numbers in the jejunum  ●elevated Occludin protein abundance in the jejunum  ●pterostilbene supplementation diminished plasma DAO and D-lactate concentrations  ●pterostilbene supplementation increased the activities of SOD, GSH-Px, and GST, the gene abundances of *NQO1* and *SOD2*, and the protein abundances of Nrf2, NQO1, and SOD2 but lowered MDA and 8-OHdG levels in the jejunum | [66] |
| Weaned piglets  21-day-old | Resveratrol or pterostilbene | IUGR  Dose: 300 mg/kg  Duration: 14 days | ●no effects on BW, ADG, ADFI, and F/G  ●raised VH but reduced apoptotic index in the jejunum  ●lowered DAO and D-lactate levels in plasma but lifted the mRNA and protein expressions of ZO-1 and Occludin in the jejunum  ●resveratrol supplementation only raised SOD and GSH levels, SOD2 mRNA and protein levels, and Nrf2 protein level in the jejunum; however, pterostilbene supplementation increased SOD, GST, GSH, and GSH/GSSG levels, *NQO1* and *SOD2* mRNA levels, and Nrf2, NOQ1, SOD1, SOD2 protein levels but lowered MDA level in the jejunum  ●resveratrol supplementation raised the abundances of Bacteroidetes, *Prevotella*, *Faecalibacterium*, and *Parabacteroides* but decreased the abundances of Proteobacteria, *Escherichia*, and *Actinobacillus* in the cecum; however, pterostilbene supplementation only decreased Proteobacteria abundance  ●resveratrol supplementation elevated acetate and butyrate concentrations in the cecum | [67] |
| Weaned piglets  28-day-old | Curcumin and resveratrol | Dose: 300 mg/kg  Duration: 28 days | ●increased ADG and ADFI but lowered F/G  ●increased the apparent digestibility of crude fat and dry matter  ●increased lactase and sucrase activities as well as VH and V/C in the jejunum and ileum  ●decreased plasma DAO and D-lactate concentrations  ●increased GSH, SOD, T-AOC, and GSH/GSSG levels but lowered MDA level in the jejunum and ileum  ●raised jejunal *SOD1*, *CAT*, and *HO-1* gene expressions and ileal *CAT* and *HO-1* gene expressions  ●increased *Occludin* gene expression in the jejunum and *Occludin*, *Claudin-1*, and *ZO-1* gene expressions in the ileum | [68] |
| Weaned piglets  28-day-old | Curcumin or/and resveratrol | Dose: 300 mg/kg  Duration: 28 days | ●lowered IL-1β and TNF-α concentrations in the jejunum and ileum but raised IgA and IgG concentrations in serum and ileum  ●lowered *IL-1β* mRNA level but raised *IL-10* mRNA level in the jejunum  ●lowered *TLR4* mRNA level in the jejunum and ileum and *TNF-α* mRNA level in the ileum  ●curcumin supplementation alone lowered *Escherichia coli* number in the jejunum and ileum  ●resveratrol supplementation alone increased the microbial diversity index in the ileum and the number of *Lactobacillus* in the jejunum and ileum | [69] |
| Weaned piglets  26-day-old | Curcumin | IUGR  Dose 400 mg/kg  Duration: 24 days | ●raised BW and ADFI  ●reduced serum TNF-α, IL-1β and IL-6 levels  ●attenuated hepatic insulin resistance and lipid accumulation  ●raised *Gys2*, *PPARα*, and *LXRα* mRNA levels and diminished *Irs1*, *Pik3c3*, *Gsk3α*, *FAS*, *CD36*, *SCD1*, and *SREBP* mRNA levels in the liver | [70] |
| Weaned piglets  26-day-old | Curcumin | IUGR  Dose: 400 mg/kg  Duration: 24 days | ●raised BW and FI  ●raised serum T-AOC, CAT, and GR levels but lowered serum AST and ALT levels  ●raised T-AOC, GSH-Px, and GR levels but lowered MDA and H_2_O_2_ levels in the liver  ●raised hepatic *Nrf2* and *GSH-Px* gene expressions and Nrf2 protein expression | [71] |
| Weaned piglets  26-day-old | Curcumin | IUGR  Dose: 200 mg/kg  Duration: 89 days | ●no effects on BW, ADG, and F/G but lowered ADFI  ●reduced drip loss at 24 h and 48 h but raised a* value in the leg muscle  ●increased the activities of CAT and SOD and lowered MDA and PC contents in the leg muscle  ●raised the mRNA abundances of *GST*, *HO-1*, *CAT*, *Nrf2*, *NQO1*, *GCLM*, and *GGT* and the protein abundances of Nrf2 and NQO1 in the leg muscle | [72] |
| Weaned piglets  26-day-old | Curcumin | IUGR  Dose: 200 mg/kg  Duration: 89 days | ●raised T-SOD and T-AOC concentrations, *Nrf2*, *SOD1*, *GCLC*, *GCLM*, and *NQO1* gene abundances, and Nrf2 and NQO1 protein abundances but lowered MDA concentration and *TNF-α*, *IL-6*, and *IFN-γ* gene abundances in the jejunum  ●raised *Occludin* gene abundance but lowered *caspase3* and *Bax* gene abundances in the jejunum | [73] |
| Weaned piglets  21-day-old | Eugenol | TGEV challenge  Dose: 400 mg/kg  Duration: 15 days | ●raised ADG but reduced F/G and diarrhea rate  ●raised serum IgG level and lowered serum TNF-α and D-lactate levels  ●lowered the gene and protein expression of NF-κB p65 in the jejunum and ileum  ●raised VH and V/C in the jejunum and ileum  ●raised Occludin and ZO-1 protein expressions and *GLUT-2* and *CaT-1* gene expressions in the jejunum | [74] |
| Weaned piglets  7.19 kg | Garcinol | Dose: 200, 400, and 600 mg/kg  Duration: 14 days | ●raised BW and ADG but reduced F/G and diarrhea incidence  ●raised serum T-AOC, SOD, CAT, and GSH-Px levels but lowered serum MDA, DAO, and D-lactate levels  ●raised VH and V/C in the jejunum and ileum but lowered CD in the jejunum  ●elevated ZO-1 and Occludin protein expressions in the jejunum, ZO-1, Occludin, and Claudin-1 protein expressions in the ileum, and *ZO-1*, *Occludin*, and *Claudin-1* mRNA expressions in the jejunum  ●lowered TNF-α, IL-1β, and IL-6 levels in serum and NF-κB p65 protein expression in the jejunum and ileum  ●raised the diversity and richness of microbiota and the abundance of *Lactobacillus* but lowered the abundance of *Escherichia coli* in the colon | [75] |
| Weaned piglets  25-day-old | Apple polyphenols | Dose: 400 and 800 mg/kg  Duration: 42 days | ●no effects on BW, ADG, ADFI, and F/G  ●raised T-AOC and HDL-C levels but lowered TC, TG, and LDL-C levels in serum  ●raised T-AOC and CAT levels and *CAT*, *GST*, and *SOD1* mRNA levels but lowered TC, TG, and LPL levels in the liver  ●diminished *ACC*, *HMG‐CoAR*, *CTP7A1*, *CD36* and *FATP-1* mRNA levels but raised *PGC-1α*, *Sirt1*, and *CPT1* mRNA levels in the liver | [76] |
| Weaned piglets  25-day-old | Apple polyphenols | Dose: 400 and 800 mg/kg  Duration: 42 days | ●raised VH and V/C in the jejunum  ●increased the Occludin protein expression in the jejunum  ●elevated the concentrations for T-AOC, T-SOD, and GSH-Px and the protein expressions for HO-1, NQO1, and nuclear Nrf2 in the jejunum  ●lowered *TNF-α*, *IL-1β*, *TLR4*, *NF-κB*, and *IL-8* gene expressions in the jejunum | [77] |
| Weaned piglets  30-day-old | Tea polyphenols | Dose: 2,000 mg/kg  Duration: 14 days | ●reduced the contents of fecal ammonia, p-cresol, phenol, and skatole  ●raised the *Lactobacillus* number but lowered the numbers of Bacteroidaceae and *Clostridium perfrigens* in feces  ●lowered pH value and raised the contents of acetate, lactic acid, and SCFA in feces | [78] |
| Weaned piglets  28-day-old | Tea polyphenols | Diquat challenge  Dose: 500 mg/kg  Duration: 21 days | ●raised ADG and ADFI from days 7 to 21  ●promoted the recovery of T lymphocyte proliferation and activation | [79] |
| Weaned piglets  35-day-old | Holly polyphenols | LPS challenge  Dose: 250 mg/kg  Duration: 16 days | ●reduced plasma AST and GGT levels  ●raised plasma albumin and total protein levels  ●raised GSH level but lowered MDA level in the liver  ●reduced TNF-α, IL-6 and IL-1β levels in plasma and liver, and *IL-6*, *TNF-α*, *COX-2*, *MyD88*, *TRAF6* and *NOD1* mRNA levels in the liver | [80] |
| Weaned piglets  35-day-old | Holly polyphenols | LPS challenge  Dose: 250 mg/kg  Duration: 16 days | ●no effects on ADG, ADFI, and F/G  ●reduced plasma TNF-α and IL-6 levels  ●raised lactase activity and the mRNA levels of *Claudin-1* and *HSP70* in the jejunum  ●elevated cecal valerate level and colonic acetate and isovalerate levels  ●raised Firmicutes abundance but reduced Bacteroidetes abundance in the colon  ●increased *Prevotella_9* abundance in the cecum and *Lactobacillus* abundance in the cecum and colon | [81] |
| Weaned piglets  35-day-old | Holly polyphenols | Diquat challenge  Dose: 250 mg/kg  Duration: 21 days | ●lifted VH and V/C in the jejunum and ileum  ●increased the activities of lactase, sucrase, and maltase in the jejunum and ileum  ●raised the jejunal ratio of protein/DNA and ileal protein content  ●increased the activities of GSH-Px and GSH content in the jejunum and reduced MDA content in the ileum  ●lowered TFR1 protein expression but raised HSPβ1, SLC7A11, and GPX4 protein expression in the jejunum and ileum | [82] |
| Weaned piglets  35-day-old | Holly polyphenols | Diquat challenge  Dose: 100 mg/kg  Duration: 21 days | ●improved hepatic morphology and reduced the activities of plasma AST, ALT, and GGT  ●raised T-AOC and GSH levels in the liver  ●lowered TFR1 mRNA and protein levels, raised *GPX4*, *SLC7A11*, *HSPβ1* mRNA levels, and improved GPX4 protein level in the liver | [83] |
| Piglets  10.6 kg | Olive polyphenols | Dose: 340 mg/kg  Duration: 21 days | ●elevated GGT level in serum and diminished 8-OHdG level in the liver | [84] |
| Piglets  10 kg | Grape seed and grape marc meal extract | Dose: 1,000 mg/kg  Duration: 28 days | ●no effects on BW, ADG, and ADFI but lowered F/G  ●lowered the counts of fecal *Streptococcus* spp. and *Clostridium Cluster* XIVa  ●lowered the expression of pro-inflammatory genes in the duodenum, ileum, and colon | [85] |
| Piglets  42-day-old | Grape seed and grape marc meal extract | Dose: 1,000 mg/kg  Duration: 28 days | ●no effects on BW, ADG, and ADFI but decreased F/G  ●lowered the transactivation of NF-κB and Nrf2 in the duodenum | [86] |
| Weaned piglets  25-day-old | Polyphenols (source from olive mill wastewater) | Dose 1,000 mg/kg  Duration: 40 days | ●raised ADG from days 0 to 20 and days 21 to 40, but lowered diarrhea score  ●lowered plasma TBARS and PC concentrations | [87] |
| Weaned piglets  21-day-old | Polyphenols (source from cocoa beans or grape seeds) | ETEC challenge  Dose: 1,000 mg/kg  Duration: 16 days | ●no effects on ADG, ADFI, and F/G but decreased diarrhea | [88] |
| Weaned piglets  21-day-old | Benzoic acid, thymol, eugenol mixtures | Dose: 3,000 mg/kg  Duration: 44 days | ●no effects on BW, ADG, ADFI, and F/G  ●raised apparent digestibility of dry matter, organic matter, crude protein, gross energy, and digestible energy on days 15 to 29  ●lowered the abundances of *Escherichia–Shigella* and *Campylobacter* in the cecum on day 14 | [89] |
| Weaned piglets  32-day-old | Cinnamaldehyde, carvacrol, and thymol mixtures | LPS challenge  Dose: 50 mg/kg  Duration: 21 days | ●no effects on ADG, ADFI, and F/G but reduced diarrhea rate  ●reduced MDA content in the duodenum  ●reduced maltase activity and raised *IL-6* mRNA level in the jejunum | [90] |
| Growing pigs  24.47 kg | Micelle silymarin | Dose: 250, 1,000, and 2,000 mg/kg  Duration: 42 days | ●linearly increased ADG on days 1 to 21 and days 1 to 42  ●linearly raised apparent digestibility of dry matter, nitrogen, and digestible energy  ●linearly raised *Lactobacillus* population in feces on day 21, and lowered *Escherichia coli* population on day 1 and 42 | [91] |
| Growing pigs  26.95 kg | Quercetin | LPS challenge  Dose: 1,000 mg/kg  Duration: 42 days | ●higher ADG  ●higher dry matter and nitrogen apparent digestibility  ●reduced blood IL-6 level but increased blood IgG and white blood cell levels and lymphocytes percentage | [92] |
| Growing pigs  25.32 kg | Magnolol | Dose: 400 mg/kg  Duration: 35 days | ●no effects on BW, ADG, ADFI, and F/G  ●raised serum albumin, total protein, IgG, IgM, and IL-22 levels  ●increased serum arginine level and glutamate level in the muscle  ●increased the mRNA levels for *SLC38A2*, *SLC1A5*, and *SLC7A1* in the jejunum and *SLC1A5* and *SLC7A1* in the ileum | [93] |
| Growing pigs  25.32 kg | Magnolol | Dose: 400 mg/kg  Duration: 35 days | ●lowered skatole concentrations in the cecum, colon, and feces  ●decreased the abundance of *Lachnospira*, *Faecalibacterium*, *Paramuribaculum*, *Faecalimonas*, *Desulfovibrio*, *Bariatricus*, and *Mogibacterium* in the colon  ●altered tryptophan metabolism pathway | [94] |
| Growing pigs  26.56 kg | Eugenol or cinnamaldehyde | Dose: 1,000 mg/kg  Duration: 35 days | ●no effects on BW, ADG, ADFI, and F/G  ●decreased fecal ammonia and hydrogen sulfide contents as well as *Escherichia coli* count | [95] |
| Growing-finishing pigs  22.27 kg | Daidzein | Dose: 37.5 and 62.5 mg/kg  Duration: 96 days | ●raised BW and ADG  ●elevated serum IGF-1, testosterone, SOD, and T-AOC levels  ●increased IMF proportion but lowered drip loss and shear force in the LT muscle  ●elevated *MyHC I* mRNA level but decreased *MyHC IIb* mRNA level in the LT muscle  ●lowered *HSL* mRNA level but raised *FAS* and *ACC1* mRNA levels in the LT muscle  ●lowered fat accumulation and *ACC1* mRNA level in the liver | [96] |
| Growing-finishing pigs  26.95 kg | Dihydromyricetin | Dose: 100, 300, and 500 mg/kg  Duration: 105 days | ●raised Claudin-1 and Occludin protein expressions in the jejunum but lowered serum DAO concentration  ●raised the mRNA expressions for *MUC1* and *MUC2* in the jejunum  ●lifted amylase and maltase activities and the mRNA expressions of *SGLT1*, *GLUT2*, and *PepT1* in the jejunum  ●reduced *Escherichia coli* population in the cecum and colon but raised *Lactobacillus* population in the cecum  ●increased butyric acid content in the cecum and colon and valeric acid content in the cecum  ●lowered IL-1β content in serum and TNF-α protein expression in the jejunum but raised IL-10, IgM, and IgA contents in serum and sIgA and IL-10 contents in the jejunum | [97] |
| Growing-finishing pigs  26.95 kg | Dihydromyricetin | Dose:100, 300, and 500 mg/kg  Duration: 105 days | ●no effects on BW, ADG, ADFI, and carcass traits but lowered F/G  ●raised crude protein content and the value for a* at 45 min and 24 h but lowered shear force and the values for L* and b* at 24 h in the LD muscle  ●higher slow MyHC and myoglobin protein abundances and the *MyHC Ⅰ*, *MyHC Ⅱa*, and myoglobin mRNA abundances in the LD muscle, and the lower fast MyHC protein abundance and *MyHC Ⅱb* mRNA abundance were observed  ●down-regulated HSPβ1 protein expression and up-regulated TNNC1 protein expression  ●raised SDH level and reduced LDH level in the LD muscle  ●raised *CaMKKβ*, *AMPKα1*, and *NRF1* gene expressions and NRF1, PGC1-α, Sirt1, and P-CaMKKβ protein expressions | [98] |
| Growing-finishing pigs  26.95 kg | Dihydromyricetin | Dose: 100, 300, and 500 mg/kg  Duration: 105 days | ●raised T-SOD, GSH-Px, GSH, and HDL-C levels in serum, CAT and T-SOD levels in the LD muscle, and GSH level in liver, but decreased TG and leptin levels in serum, TG level in the LD muscle, and MDA and TG levels in the liver  ●increased the protein expressions of total Nrf2, nuclear Nrf2, NQO1, and HO-1 and lowered Keap1 protein expression in the LD muscle and liver  ●increased the protein expressions of P-AMPK and P-ACC in the LD muscle and liver | [99] |
| Growing-finishing pigs | Dihydromyricetin | Dose: 300 mg/kg  Duration: 105 days | ●raised the activities of the T-AOC, CAT, and GSH-Px but decreased MDA content in the jejunum  ●up-regulated the protein expressions of HO-1, NQO1, nuclear Nrf2, and P-ERK in the jejunum | [100] |
| Growing-finishing pigs  26.69 kg | Chlorogenic acid | Dose: 25, 50, and 100 mg/kg  Duration: 100 days | ●BW was raised when diets supplemented with 100 mg/kg chlorogenic acid  ●dietary 50 and 100 mg/kg chlorogenic acid lifted the dressing percentage and perirenal fat and improved the *ACC* mRNA levels in the LT muscle  ●dietary 50 mg/kg raised value for pH_24 h_ in the LT muscle  ●dietary 100 mg/kg chlorogenic acid increased the contents of  crude fat, glycogen, total amino acids, flavor amino acids, and GSH-Px but decreased the contents of inosine, hypoxanthine, and MDA in the LT muscle  ●Higher *MyHC IIa*, *HO-1*, *NQO-1*, and *Nrf2* mRNA levels and lower 5’-nucleotidase cytosolic II mRNA level in the LT muscle were observed following 100 mg/kg included in the diets | [101] |
| Growing-finishing pigs  33.23 kg | Ellagic acid | Dose: 75 and 150 mg/kg  Duration: 84 days | ●increased the protein expression of slow MyHC but lowered the protein expression of fast MyHC in the LT muscle  ●elevated the number of slow-twitch muscle fibers and reduced the number of fast-twitch muscle fibers  ●raised SDH and MDH activities but decreased LDH activity in the LT muscle  ●raised mitochondrial DNA content, the mRNA expressions of *ATP5G*, *TFAM*, *AMPKα1*, *PGC-1α*, and *Sirt1*, and the protein expressions of P-LKB1, P-AMPK, Sirt1, and PGC-1α in the LT muscle | [102] |
| Growing-finishing pigs  24.67 kg | Resveratrol | Dose: 600 mg/kg  Duration: 119 days | ●no effects on BW, ADG, ADFI, F/G and carcass traits  ●lowered serum TG, TC, and LDL-C concentrations  ●lifted IMF content and *PPARγ*, *FAS*, *ACC*, and *LPL* mRNA abundances but diminished *CPT1*, *Sirt1*, and *PPARα* mRNA abundances in the LD muscle | [103] |
| Finishing pigs  66.2 kg | Naringin | Dose: 500, 1,000, and 1,500 mg/kg  Duration: 50 days | ●no effect on BW, ADG, ADFI, and F/G  ●raised lean meat percentage and loin eye area  ●dietary 1,500 mg/kg naringin raised pH_45 min_ and IMP content in the LD muscle  ●dietary 1,000 and 1,500 mg/kg naringin increased SOD and T-AOC levels in the LD muscle and SOD and GSH-Px levels in the liver  ●raised *MyHC IIa* gene level and lowered *MyHC IIb* gene level in the LD muscle | [104] |
| Finishing pigs 51 kg | Micelle silymarin | Dose: 500, 1,000, and 2,000 mg/kg  Duration: 72 days | ●linearly raised ADG from 5 to 10 weeks but no effect on carcass traits  ●linearly raised apparent digestibility of nitrogen in weeks 5 and 10  ●linearly diminished cooking loss and b* value at 45 min postmortem and linearly increased a* value at 45 min and 24 h postmortem in the LT muscle  ●linearly reduced TBARS and PC levels in the LT muscle  ●linearly raised *Lactobacillu*s population in feces in week 5 | [105] |
| Finishing pigs  45.11 kg | Mulberry leaf flavonoids | Dose: 200, 400, 800, and 1,600 mg/kg  Duration: 58 days | ●linearly raised BW and ADG but reduced F/G  ●raised lean meat percentage, loin-eye area, and marbling score  ●lowered fiber cross-sectional area, shear force, and a* value in the LL muscle  ●raised ∑n-3 PUFA level and reduced the ratio of ∑n-6 PUFA/∑n-3 PUFA in the LL muscle  ●raised serum SOD and CAT levels but reduced serum MDA level  ●raised *ABCA1*, *ACCα*, *FAS*, and *SREBP1* mRNA abundances and P-PPARγ, P-SREBP1, LXRα, and ABCA1 protein abundances in the LL muscle | [106] |
| Finishing pigs  45.11 kg | Mulberry leaf flavonoids | Dose: 200, 400, 800, and 1,600 mg/kg  Duration: 58 days | ●decreased the plasma concentrations of TC, TG, and free fatty acids as well as the serum activity of 3-hydroxy-3-methylglutaryl coenzyme A reductase  ●increased the serum activities of LPL and adipose triglyceride lipase  ●decreased the adipocyte area in the dorsal subcutaneous adipose tissue and increased the adipocyte area in the visceral adipose tissue  ●decreased C20:1 content but increased ALA and ∑n-3 PUFA contents in the dorsal subcutaneous adipose tissue, abdominal subcutaneous adipose, and visceral adipose tissues  ●promoted the activation of the PPARγ-LXRα-ABCA1 signaling pathway | [107] |
| Finishing pigs  67.47 kg | Grape seed proanthocyanidin extract | Dose: 200 mg/kg  Duration: 49 days | ●no effects on BW, ADG, ADFI, F/G and carcass traits  ●increased the value for pH_24h_ and a*, decreased shear force, lactate content, and glycolytic potential in LD muscle  ●raised crude protein content, the proportions of ALA, C18:2n6, EPA, ∑PUFA, and ∑n-3 PUFA, and the ratio of ∑PUFA/∑SFA in the LD muscle  ●increased *MyHC I*, *MyHC IIa*, and myoglobin mRNA levels, the protein expression of slow MyHC, and the percentage of slow-twitch fibers in the LD muscle  ●raised SDH, T-AOC, T-SOD, CAT, and GSH-Px concentrations but lowered MDA concentration in the LD muscle | [108] |
| Finishing pigs  94.9 kg | Grape seed proanthocyanidin extract | Dose: 200 mg/kg  Duration: 30 days | ●raised GSH, T-AOC, and GSH-Px levels but diminished MDA level in serum, LD muscle, and liver  ●raised the mRNA expressions of *SOD1*, *SOD2*, *CAT*, and *GST* and the protein expressions of Nrf2, HO-1, and NQO1 in the LD muscle and liver  ●diminished serum TG and TC levels but raised serum HDL-C level  ●raised the mRNA expressions of *CPT1*, *PPARα*, *HSL*, *PGC-1α*, and *Sirt1* and the protein abundance of P-AMPK in the LD muscle and liver | [109] |
| Finishing pigs  71.89 kg | Chlorogenic acid | Dose: 400 mg/kg  Duration: 35 days | ●increased ADG but decreased F/G  ●lowered backfat thickness and fat percentage but increased lean percentage  ●increased *SNAT2* gene expression in the LD and BF muscles and *LAT1* gene expression in the BF muscle  ●raised serum contents of essential amino acids and total amino acids and promoted more amino acids to translocate to skeletal muscles  ●raised P-AKT, P-mTOR, S6K1, and 4EBP1 protein abundances in the LD muscle | [110] |
| Finishing pigs  71.89 kg | Chlorogenic acid | Dose: 400 mg/kg  Duration: 30 days | ●diminished the value for b* in LD muscle and raised the content for IMP in the LD and BF muscles  ●improved the proportions of flavor amino acids, essential amino acids, and total amino acids in the LD and BF muscles  ●raised the mRNA expressions of *SOD1*, *Nrf2*, and *GPX1* in the  LD and BF muscles  ●lowered the muscle fiber diameter of LD and BF muscles  ●raised the mRNA expressions of *MyHC I*, *MyHC IIa*, *MyoD*, and *MyoG* in the LD muscle | [111] |
| Finishing pigs  50.88 kg | Chlorogenic acid | Dose: 500 mg/kg  Duration: 60 days | ●raised the diversity of the colonic microbiota and the colonic abundances of *Neisseria*, *Actinomyces*, *Lachnobacterium*, *Porphyromonas*, *Pseudonocardia*, and *Mobiluncus* at the genus level but lowered the abundances of *Enterococcus* and *Wohlfahrtiimonas* at the genus level  ●increased the levels of serum aspartic acid, threonine, alanine, arginine, and colonic 5-hydroxytryptamine | [112] |
| Finishing pigs  67.6 kg | Ferulic acid | Dose: 25 ppm/kg  Duration: 27 days | ●increased ADG but lowered F/G  ●raised loin muscle area and decreased backfat thickness  ●decreased fast and glycolytic muscle fiber percentage in the LT muscle | [113] |
| Finishing pigs  82.65 kg | Rosmarinic acid or ursolic acid | Dose: 500 mg/kg  Duration: 34 days | ●no effects on BW, ADG, ADFI, ADFI, and carcass traits  ●increased TG level in the soleus muscle  ●rosmarinic acid treatment increased the gene expression levels of *FAS*, *SREBP1c*, and *PPARγ* but decreased the *ACCα* gene expression level, while ursolic acid treatment increased the *FATP-1* gene expression level  ●rosmarinic acid treatment increased the microbial richness and diversity with a concurrent enrichment in the relative abundance of *Bacteroides* and *g-UCG-005*, while ursolic acid only enriched the relative abundance of *Prevotella* | [114] |
| Finishing pigs  65 kg | Resveratrol | Dose: 200, 400, and 600 mg/kg  Duration: 41 days | ●no effects on BW, ADG, and ADFI  ●raised *MyHC I* and *MyHC IIa* mRNA levels but lowered *MyHC IIb* mRNA level in the LT muscle  ●raised SDH and MDH activities but diminished LDH activity  ●raised AdipoQ, AdipoR1, AdipoR2, AMPKα1, AMPKα2, and PGC-1α mRNA and protein levels in the LT muscle | [115] |
| Finishing pigs  150-day-old | Thymol | Dose: 100 mg/kg  Duration: 42 days | ●no effects on BW and F/G but lowered BWG  ●reduced drip loss in the LD muscle  ●raised *MyHC I* mRNA level and MyHC IIa protein level but lowered MyHC IIb protein level in the LD muscle  ●raised SDH level but lowered LDH level  ●elevated PGC-1α mRNA and protein levels | [116] |
| Finishing pigs  71.25 kg | Apple polyphenols | Dose: 400 and 800 mg/kg  Duration: 49 days | ●lowered hepatic fat deposition, reduced hepatic MDA, TC, and TG levels, raised hepatic *Nrf2*, *GSH-Px*, *CAT*, *SOD1*, *HSL*, *CPT1*, *PPARα*, *CTP7A1*, and low-density lipoprotein receptor mRNA levels, and lowered C16:0 and C20:4n-6 proportions and Δ9-18 dehydrogenase activity in the liver | [117] |
| Finishing pigs  71.25 kg | Apple polyphenols | Dose: 400 and 800 mg/kg  Duration: 49 days | ●no effects on BW, ADG, ADFI, and F/G but decreased backfat thickness and abdominal adipose tissue index  ●decreased urea nitrogen level in the blood, and TC level and the values for L* and b* in the LD muscle  ●raised crude protein and IMP contents, the proportions of essential amino acids, flavor amino acids, and total amino acids, and the mRNA levels of *SLC7A1*, *SLC7A2*, *SLC7A7*, *SLC1A2* in the LD muscle  ●raised DHA and ∑PUFA proportions and the ratio of ∑PUFA/∑SFA in the LD muscle | [118] |
| Finishing pigs  71.25 kg | Apple polyphenols | Dose: 400 and 800 mg/kg  Duration: 49 days | ●raised the gene expressions of *MyHC I* and *MyHC IIa* and the protein expression of MyHC I but lowered the MyHC IIb protein expression in the LD muscle  ●lowered LDH level but raised SDH and MDH levels in the LD muscle  ●raised the mRNA expressions of *AMPKα1*, *Sirt1*, *PGC-1α*, *TFAM*, *TFB1M*, *Cytc*, *ATP5G*, and *CPT1* and the protein expressions of Sirt1, PGC-1α, Cytc, and P-AMPK in the LD muscle  ●raised T-SOD, GSH-Px, and CAT levels and *SOD1*, *SOD2*, *CAT*, *GSH-Px*, and *Nrf2* mRNA levels but decreased MDA level and *Keap1* mRNA level in the LD muscle | [119] |
| Finishing pigs  71.25 kg | Apple polyphenols | Dose: 400 and 800 mg/kg  Duration: 49 days | ●raised V/C but lowered CD in the ileum  ●increased ZO-1, Occludin, and Claudin-1 protein expressions in the jejunum  ●lifted IgA level in serum and SIgA level in the ileum  ●raised *Lactobacillus* and *Bifidobacterium* populations in the cecum and *Bifidobacterium* population in the colon but lowered *Escherichia coli* population in the colon  ●raised T-AOC and CAT levels in serum, T-AOC level, *SOD1* mRNA level, and Nrf2, NQO1, and HO-1 protein levels in the jejunum but lowered MDA level and *Keap1* mRNA level in the jejunum and ileum | [120] |
| Finishing pigs  140 kg | Polyphenols (source from olive mill wastewater) | Dose: 30 mg/kg  Duration: 120 days | ●raised the number of intra-epithelial and lamina propria leukocytes in the intestine  ●reduced COX-2 level in the cecum and colon  ●lowered superoxide anion production in blood leukocytes and alveolar macrophages | [121] |
| Finishing pigs  88.9 kg | Red-osier dogwood polyphenol extract | Dose: 5,000 mg/kg  Duration: 15 days | ●no effects on ADG, ADFI, and F/G  ●increased the diversity and numbers of microbiota and the abundances of Bacilli, Lactobacillales, Lactobacillaceae*,* *Lactobacillus delbrueckii*, and *Lactobacillus mucus* in the ileum | [122] |
| Sows | Daidzein | Dose: 200 mg/kg  Duration: from days 1 to 34 of gestation | ●enhanced the number of viable embryos  ●elevated estrogen, progesterone, and IGF-1 levels in the amniotic fluid  ●raised GSH-Px level but lowered TNF-α level in the amniotic fluid  ●raised arginine, creatine, and citrate levels in the amniotic fluid | [123] |
| Sows | Daidzein | Dose: 200 mg/kg  Duration: from days 1 to 35 of gestation | ●increased the total number of embryos and serum progesterone and estradiol-17β levels  ●raised serum T-AOC and GSH-Px levels but reduced serum IL-6 and MDA levels  ●raised CAT, GSH-Px, T-SOD, and T-AOC levels but diminished IL-1β, TNF-α, and IL-6 levels in the ovarian tissue  ●raised Nrf2, HO-1, and NQO1 protein expressions but decreased TLR4, P-NFκB, P-AKT, and P-IκBα protein expressions in the ovarian tissue | [124] |
| Sows | Daidzein | Dose: 200 mg/kg  Duration: from days 1 to 110 of gestation | ●increased the total number of piglets born per litter and the number of piglets born alive per litter  ●increased serum estrogen and progesterone concentrations of sows on day 35 of gestation and decreased farrowing duration  ●increased serum IgG, SOD, and GSH-Px levels on day 35 of gestation and serum SOD and T-AOC levels on day 85 of gestation  ●elevated the mRNA levels for *LC38A1* and *IGF-1* in the placenta | [125] |
| Sows | Soy isoflavones | Dose: 10, 20, and 40 mg/kg  Duration: from day 90 of gestation to day 21 of lactation | ●linearly increased ADFI of sows  ●linearly increased serum SOD and T-AOC levels but lowered serum MDA level of sows on day 10 of lactation  ●linearly raised BW on days 10 to 21 of lactation, and ADG on days 3 to 10 and 3 to 21 of lactation in suckling piglets | [126] |
| Sows | Soy isoflavones and astragalus polysaccharide | Dose: 200 mg/kg  Duration: from days 1 to 21 of lactation | ●raised ADFI and total lactation yield of sows  ●lowered serum TG, TC, and MDA contents of sows  ●raised serum IgA, GH, IGF-1, PRL, and SOD contents of sows | [127] |
| Sows | Silymarin | Dose: 250 and 500 mg /kg  Duration: from day 85 of gestation to day 17 of lactation | ●raised ADFI of sows  ●raised urea content in regular milk  ●decreased serum MDA content on day 90 of gestation and day of farrowing but raised serum T-AOC content on day 17 of lactation | [128] |
| Sows | Silymarin | Dose: 40 g/day  Duration: from day 108 of gestation to weaning | ●raised colostrum yield and ADFI but reduced farrowing duration of sows  ●raised litter weight and BW at weaning and ADG of piglets  ●raised serum CAT level on day 18 of lactation and serum GSH-Px and PRL levels on day 7 of lactation in sows  ●lowered serum TNF-α level on day 7 of lactation and serum IL-1β and urea levels on day 18 of lactation in sows  ●raised protein and urea levels in milk on day 18 of lactation and improved lactose level in colostrum | [129] |
| Sows | Micelle silymarin | Dose: 500, 1,000, and 2,000 mg/kg  Duration: from day 1 of gestation to day 21 of lactation | ●linearly elevated born alive rate of piglets  ●linearly raised litter weight at weaning, litter weight gain, BW, and ADG of piglets  ●linearly reduced BW loss but raised average daily milk yields of sows during lactation  ●raised fat content in milk on day 14 of lactation  ●reduced serum AST activity in sow on day of 21 lactation  ●lifted serum activity of SOD at parturition and lowered GSSG concentration, and GSSG/GSH ratio on day 21 of lactation in sows  ●raised serum CAT and T-AOC concentrations but diminished serum MDA concentration in suckling piglets on day of 14 lactation | [130] |
| Sows | Resveratrol | Dose: 300 mg/kg  Duration: from day 20 of gestation to day 21 of lactation | ●reduced drip loss and lactic acid level but increased the pH_24 h_ of LT muscle of offspring  ●increased IMF content, SOD activity, and *SOD2* mRNA expression but diminished MDA content in the LT muscle of offspring  ●increased the mRNA and protein expression of MyHC I and decreased the mRNA and protein expression of MyHC IIb in the LT muscle of offspring | [131] |
| Sows | Resveratrol | Dose: 300 mg/kg  Duration: from day 75 of gestation to day 21 of lactation | ●raised the number of live births and litter weight at weaning, and raised the relative abundances of *Lactobacillus* and *Alloprevotella* but decreased the relative abundance of *Escherichia-shigella* in piglet feces  ●raised plasma SOD level on day 110 of gestation and day 14 of lactation, plasma GSH-Px and T-AOC levels on day 14 of lactation, and decreased plasma MDA level on day 14 of lactation in sows  ●raised the contents of IgA, IgG, and IgM in the colostrum  ●raised the plasma GH and progesterone levels in sows | [132] |
| Sows | Resveratrol | Dose: 300 mg/kg  Duration: from day 20 of gestation to day 21 of lactation | ●raised ADG of suckling piglets from days 14 to 21 and days 21 to 28 of lactation, and lowered fecal scores of piglets from days 15 to 21 after birth and diarrhea from days 3 to 5 of post-weaning  ●increased VH and V/C in the jejunum of weaning and post-weaning piglets, lowered IL-6 and TNF-α levels in the jejunum in both weaning and post-weaning piglets and raised the proportion of butyrate-producing bacteria, such as *Flavonifractor*, *Odoribacter* and *Oscillibacter* in the feces of piglets | [133] |
| Sows | Resveratrol | Dose: 300 mg/kg  Duration: from day 20 of gestation to day 21 of lactation | ●raised lactose level in colostrum and total solids and fat levels in 21-d milk  ●raised HDL-C, LDL-C, lipase, and insulin levels in plasma of suckling piglets  ●elevated the enzyme activities of HSL, ACC, and LPL and the mRNA levels of *ACCα*, *LPL*, *FATP-1*, and *C/EBPα* in the adipose tissue of suckling piglets | [134] |
| Sows | Resveratrol | Dose: 300 mg/kg  Duration: from day 20 of gestation to day 21 of lactation | ●increased litter weight and piglet weight at weaning  ●increased plasma CAT and GSH-Px levels in newborn piglets and weaning piglets  ●raised plasma SOD level on days 14 and 21 of lactation and plasma CAT and GSH-Px levels on day 110 of gestation in sows  ●increased SOD, GSH-Px, and CAT levels and decreased MDA and H_2_O_2_ levels in the placenta  ●increased the gene expressions of *CAT*, *GPX1*, *GPX4*, *SOD1*, *HO-1*, *GCLM*, *MGST1*, *UGT1A1* and the protein expressions of Nrf2, Sirt1, and P-NFκB in the placenta  ●increased SOD and GSH-Px levels but decreased MDA and H_2_O_2_ levels in milk | [135] |
| Sows | Hydroxytyrosol and linseed oil | Dose: 1.5 mg/kg  Duration: from day 35 of gestation to childbirth | ●higher ADG and fractional growth rate of offspring from days 15 to 60 after birth  ●lowered plasma TC, HDL-C, and LDL-C levels of offspring on day 120 after birth  ●raised ∑n-3 PUFA level in subcutaneous fat and liver as well as lowered ∑n-6/∑n-3 PUFA ratio in subcutaneous fat, LD, and BF muscles of offspring on day 60 after birth | [136] |
| Sows | Hydroxytyrosol and linseed oil | Dose: 1.5 mg/kg  Duration: from days 35 to 100 of gestation | ●raised ALA, EPA, DHA, and ∑n-3 PUFA levels in the LD muscle of fetuses | [137] |
| Sows | Hydroxytyrosol | Dose: 1.5 mg/kg  Duration: from days 35 to 100 of gestation | ●improved antioxidant status and glucose metabolism and reduced DNA methylation in IUGR fetus | [138] |
| Sows | Hydroxytyrosol | Douse 1.5 mg/kg  Duration: from days 35 to 100 of gestation | ●elevated ∑n-3 PUFA and ∑n-6 PUFA levels in the fetal LD muscle with IUGR; however, it increased the ∑n-6 PUFA/∑n-3 PUFA ratio and the desaturation index | [139] |
| Sows | Garcinol | Dose: 200 and 600 mg/kg  Duration: from day 90 of gestation to day 21 of lactation | ●no effects on BW and backfat thickness of sows  ●raised litter birth weight, litter weaning weight, and litter gain, and reduced piglet mortality  ●A higher T-AOC, SOD, GSH-Px, and CAT levels, and a lower MDA level in the plasma of sows were observed on both day 110 of gestation and day 21 of lactation when they were fed with 600 mg/kg garcinol  ●dietary 600 mg/kg garcinol facilitated acid-base balance of blood of newborn piglets and raised plasma IgA and IgG levels in piglets on day 14 of lactation  ●supplementation with 600 mg/kg garcinol lifted IgA and IgG levels in colostrum and crude protein, IgA, and IgG levels in 17-d milk | [140] |
| Sows | Grape seed polyphenols | Dose: 200 and 300 mg/kg  Duration: from day 80 of gestation to day 21 of lactation | ●the number of dead fetuses was reduced, the litter survival rate and preweaning survivability were improved, and the serum levels of progesterone and estradiol of sows increased when sows fed diets containing 300 mg/kg grape seed polyphenols  ●raised GSH-Px and SOD levels in serum on day 110 of gestation, and IgM and IgG levels in colostrum in sows | [141] |
| Sows | *Lonicera flos* and *Sucutellaria baicalensis* mixed extracts | Dose: 500 mg/kg  Duration: from day 85 of gestation to childbirth | ●increased the number of alive and healthy piglets and the litter weight at birth  ●increased the platelet counts, the levels of PRL and GH, and the content of IL-2 in umbilical cord serum  ●increased IgA and IgM contents in the colostrum | [142] |

Abbreviations: a* = redness; ABCA1 = ATP binding cassette subfamily A member 1; ACC = acetyl-CoA carboxylase; ACCα = acetyl coenzyme A-α; ADFI = average daily feed intake; ADG = average daily gain; AdipoQ = Adiponectin; AdipoR1 = Adiponectin receptor 1; AdipoR2 = Adiponectin receptor 2; AhR = aryl hydrocarbon receptor; ALA = α-linolenic acid; ALT = alanine aminotransferase; AMPKα1 = AMP-activated protein kinase α 1; AMPKα2 = AMP-activated protein kinase α 2; AST = aspartate aminotransferase; ATCH = adrenocorticotropic hormone; ATP5G = ATP synthase membrane subunit c locus; b* = yellowness; bile acids = BAs; Bax = B-cell lymphoma-2-associated X protein; Bcl2 = B-cell lymphoma-2; BF = *biceps femoris*; BW = body weight; BWG = body weight gain; caspase-3 = cysteinyl aspartic acid protease-3; caspase-9 = cysteinyl aspartic acid protease-9; CaMKKβ = calmodulin-dependent protein kinase kinase-β; CAT = catalase; CaT-1 = cationic amino acid transporter 1; CD = crypt depth; CD36 = cluster of differentiation 36; C/EBPα = CCAAT-enhancer-binding protein α; COX1 = cytochrome c oxidase subunit 1; COX-2 = cyclooxygenase-2; CPT1 = carnitine-palmitoyl transferase 1; CTP7A1 = cholesterol 7α-hydroxylase; CYP1A1 = cytochrome P450 family 1 member A1; Cytc = cytochrome c; DAO = diamine oxidase; DHA = docosahexaenoic acid; DMT1 = divalent metal transporter-1; DON = deoxynivalenol; EGFR = epidermal growth factor receptor; EPA = eicosapentaenoic acid; ETEC = enterotoxigenic *Escherichia coli*; FAS = fatty acid synthase; FATP-1 = fatty acid transport protein-1; FATP-4 = fatty acid transport protein-4; FD4 = fluorescein isothiocyanate dextran; F/G = feed to gain ratio; FI = feed intake; GBP1 = guanylate-binding protein-1; GCLC = glutamate-cysteine ligase catalytic subunit; GCLM = glutamate-cysteine ligase modifier subunit; GGT = gamma-glutamyl transferase; GH = growth hormone; GLUT-2 = glucose transporter-2; GPX1 = glutathione peroxidase 1; GPX4 = glutathione peroxidase 4; GR = glutathione reductase; GSH = glutathione; GSH-Px, glutathione peroxidase; Gsk3α = glycogen synthase kinase 3 α; GSSG = oxidized glutathione; GST = glutathione S-transferase; Gys2 = glycogen synthase 2; HDL-C = high-density lipoprotein cholesterol; HMG‐CoAR = 3-hydroxy-3-methyl-glutaryl coenzyme A reductase; HO-1 = heme oxygenase-1; HSL = hormone-sensitive lipase; HSPβ1 = heat shock protein β1; HSP70 = heat shock protein 70; ICAM1 = intercellular cell adhesion molecule 1; IFN-γ = interferon γ; IgA = immunoglobulin A; IGF-1 = insulin-like growth factor-1; IgG = immunoglobulin G; IgM = immunoglobulin M; IL-1β = interleukin-1 β; IL-2 = interleukin-2; IL-4 = interleukin-4; IL-6 = interleukin-6; IL-10 = interleukin-10; IL-12 = interleukin-12; IL-13: interleukin-13; IL-22 = interleukin-22; IMF = intramuscular fat; IMP = inosinic acid; Irs1 = insulin receptor substrate 1; IUGR = intrauterine growth retardation; L* = lightness; LAT1 = L-type amino acid transporter 1; LD = *longissimus dorsi*; LDH = lactate dehydrogenase; LDL-C = low-density lipoprotein cholesterol; LL = *longissimus lumborum*; LPL = lipoprotein lipase; LPS = lipopolysaccharide; LT = *longissimus thoracic*; LXRα = liver X receptor α; MDA = malondialdehyde; MDH = malate dehydrogenase; MEF2C = myocyte enhancer factor 2C; MGST1 = microsomal glutathione S-transferase 1; MPO = myeloperoxidase; MTTP = microsomal triglyceride transfer protein; MUC1 = mucin 1; MUC2 = mucin 2; MX1 = myxovirus resistance protein 1; MX2 = myxovirus resistance protein 2; MyD88 = myeloid differentiation factor 88; MyHC = myosin heavy chain; MyoD = myogenic differentiation factor 1; MyoG = myogenin; NLRP3 = Nod-like receptor pyrin domain containing 3; NOD1 = nucleotide-binding oligomerization domain protein 1; NQO1 = quinone oxidoreductase 1; NRF1 = nuclear respiratory factor 1; Nrf2 = nuclear factor-erythroid 2-related factor 2; OSO = oxidized soybean oil; P-ACC= phosphorylated ACC; P-AKT = phosphorylated protein kinase B; P-AMPK = phosphorylated AMPK; PC = protein carbonyl; PCAF = P300/CBP associating factor; P-CaMKKβ = phosphorylated CaMKKβ; PEDV = porcine epidemic diarrhea virus; P-ERK = phosphorylated extracellular signal-regulated protein kinase; PepT1 = oligopeptide transporter 1; PGC-1α = peroxisome proliferator-activated receptor gamma coactivator 1 α; PINK1 = putative kinase 1; P-IκBα, phosphorylated inhibitor of kappa B α; Pik3c3 = phosphatidylinositol 3-kinase catalytic subunit type 3; P-LKB1 = phosphorylated LKB1; P-mTOR = phosphorylated mammalian target of rapamycin; P-NF-κB = phosphorylated nuclear factor kappa B; P-Nrf2 = phosphorylated nuclear factor-erythroid 2-related factor 2; PPARα = peroxisome proliferator-activated receptor α; P-PPARγ = phosphorylated peroxisome-proliferator activated receptor γ; PP2A = protein phosphatase 2A; PRL = prolactin; PRV = Pseudorabies virus; P-SREBP1 = phosphorylated sterol regulatory element binding proteins 1; PUFA = polyunsaturated fatty acid; ROS = reactive oxygen species; S6K1 = p70 ribosomal S6 kinase protein ; SCD1 = stearoyl-CoA desaturase1; SCFAs = short-chain fatty acids; SDH = succinate dehydrogenase; SFA = saturated fatty acid; SGLT1 = sodium-glucose transport protein 1; SIgA = secretory immunoglobulin A; Sirt1 = sirtuin1; SLC1A2 = solute carrier family 1 member 2; SLC1A5 = solute carrier family 1 member 5; SLC7A1 = solute carrier family 7 member 1; SLC7A2 = solute carrier family 7 member 2; SLC7A7 = solute carrier family 7 member 7; SLC7A11 = solute carrier family 7 member 11; SLC38A1 = sodium-coupled neutral amino acid transporter 1; SLC38A2 = solute carrier family 38 member 2; SNAT2 = sodium coupled neutral amino acid transporter 2; SOD = superoxide dismutase; SOD1 = superoxide dismutase 1; SOD2 = superoxide dismutase 2; SREBP = sterol regulatory element binding proteins; SREBP1 = sterol regulatory element binding proteins 1; T-AOC = total antioxidant capacity; TBARS = thiobarbituric acid reactive substances; TC = total cholesterol; TER = transepithelial electrical resistance; TFAM = mitochondrial transcription factor A; TFB1M = mitochondrial transcription factor B1; TFR1 = transferrin receptor protein 1; TG = triglyceride; TGEV = transmissible gastroenteritis virus; TLR4 = Toll-like receptor 4; TNF-α = tumor necrosis factor α; TNNI1 = troponin I-SS; TRAF6 = TNF receptor-associated factor 6; TNNC1 = Troponin C-slow; T-SOD = total superoxide dismutase; UGT1A1 = UDP glucuronosyltransferase family 1 member A1; V/C = villus height to crypt depth ratio; VCAM2 = vascular cell adhesion molecule 2; VH = villus height; ZNT1 = zinc transporter-1; ZO-1 = zonula occluden-1; ZO-2 = zonula occluden-2; 8-OHdG = 8-hydroxy-2’-deoxyguanosine; 4EBP1 = eukaryotic translation initiation factor 4E-binding protein 1

**Reference:**

1. Wu MJ, Yi D, Zhang Q, Wu T, Yu K, Peng M, et al. Puerarin enhances intestinal function in piglets infected with porcine epidemic diarrhea virus. Sci Rep. 2021;11:6552.

2. Zeng YT, Li R, Dong Y, Yi D, Wu T, Wang L, et al. Dietary supplementation with puerarin improves intestinal function in piglets challenged with *Escherichia coli* K88. Animals (Basel). 2023;13(12):1908.

3. Wu X, Liang HM, Tang Y, Chen DW, Yu B, He J, et al. Dietary ferulic acid supplementation improves antioxidant capacity and lipid metabolism in liver of piglets with intrauterine growth retardation. Anim Biotechnol. 2023;34(9):4900–9.

4. Wan J, Yu Q, Luo JQ, Zhang L, Ruan Z. Effects of ferulic acid on the growth performance, antioxidant capacity, and intestinal development of piglets with intrauterine growth retardation. J Anim Sci. 2022;100(5):skac144.

5. Zhang ZF, Wang ST, Zheng LY, Hou YQ, Guo SS, Wang L, et al. Tannic acid-chelated zinc supplementation alleviates intestinal injury in piglets challenged by porcine epidemic diarrhea virus. Front Vet Sci. 2022;9:1033022.

6. Cheng K, Ji SL, Jia PL, Zhang H, Wang T, Song ZH, et al. Resveratrol improves hepatic redox status and lipid balance of neonates with intrauterine growth retardation in a piglet model. Biomed Res Int. 2020;2020:7402645.

7. Liao P, Li YH, Li MJ, Chen XF, Yuan DX, Tang M, et al. Baicalin alleviates deoxynivalenol-induced intestinal inflammation and oxidative stress damage by inhibiting NF-κB and increasing mTOR signaling pathways in piglets. Food Chem Toxicol. 2020;140:111326.

8. Li M, Yuan DX, Liu YH, Jin H, Tan BE. Dietary puerarin supplementation alleviates oxidative stress in the small intestines of diquat-challenged piglets. Animals (Basel). 2020;10(4):631.

9. Yuan DH, Wang J, Xiao DF, Li JF, Liu YH, Tan BE, et al. *Eucommia ulmoides* flavones as potential alternatives to antibiotic growth promoters in a low-protein diet improve growth performance and intestinal health in weaning piglets. Animals (Basel). 2020;10(11):1998.

10. Li YP, Jiang XR, Wei ZX, Cai L, Yin JD, Li XL. Effects of soybean isoflavones on the growth performance, intestinal morphology and antioxidative properties in pigs. Animal. 2020;14(11):2262–70.

11. Li YP, Jiang XR, Cai L, Zhang YL, Ding HB, Yin JD, et al. Effects of daidzein on antioxidant capacity in weaned pigs and IPEC-J2 cells. Anim Nutr. 2022;11:48–59.

12. Yao WL, Wang TX, Huang L, Bao ZX, Wen S, Huang FR. Embelin alleviates weaned piglets intestinal inflammation and barrier dysfunction via PCAF/NF-κB signaling pathway in intestinal epithelial cells. J Anim Sci Biotechnol. 2022;13:139.

13. Xun WJ, Ji MY, Ma ZH, Deng TJ, Yang W, Hou GY, et al. Dietary emodin alleviates lipopolysaccharide-induced intestinal mucosal barrier injury by regulating gut microbiota in piglets. Anim Nutr. 2023;14:152–62.

14. Xu BY, Qin WX, Xu YZ, Yang WB, Chen YW, Huang JC, et al. Dietary quercetin supplementation attenuates diarrhea and intestinal damage by regulating gut microbiota in weanling piglets. Oxid Med Cell Longev. 2021;2021:6221012.

15. Li EK, Li C, Horn N, Ajuwon KM. Quercetin attenuates deoxynivalenol-induced intestinal barrier dysfunction by activation of Nrf2 signaling pathway in IPEC-J2 cells and weaned piglets. Curr Res Toxicol. 2023;5:100122.

16. Liu JH, Qiao Y, Yu B, Luo YH, Huang ZQ, Mao XB, et al. Functional characterization and toxicological study of proanthocyanidins in weaned pigs. Toxins. 2023;15(9):558.

17. Fang LY, Li MY, Zhao LY, Han SY, Li Y, Xiong BH, et al. Dietary grape seed procyanidins suppressed weaning stress by improving antioxidant enzyme activity and mRNA expression in weanling piglets. J Anim Physiol Anim Nutr (Berl). 2020;104(4):1178–85.

18. Wei XT, Li LX, Yan HS, Li QH, Gao JJ, Hao RR. Grape seed procyanidins improve intestinal health by modulating gut microbiota and enhancing intestinal antioxidant capacity in weaned piglets. Livestock Sci. 2022;264:105066.

19. Yan HS, Gao WW, Li QH, Li HQ, Hao RR. Effect of grapeseed procyanidins on small intestinal mucosa morphology and small intestinal development in weaned piglets. Anim Prod Sci. 2020;60(16):1894–901.

20. Zhang Y, Wang Y, Chen DW, Yu B, Zheng P, Mao XB, et al. Dietary chlorogenic acid supplementation affects gut morphology, antioxidant capacity and intestinal selected bacterial populations in weaned piglets. Food Funct. 2018;9(9):4968–78.

21. Chen JL, Li Y, Yu B, Chen DW, Mao XB, Zheng P, et al. Dietary chlorogenic acid improves growth performance of weaned pigs through maintaining antioxidant capacity and intestinal digestion and absorption function. J Anim Sci. 2018;96(3):1108–18.

22. Chen JL, Xie HM, Chen DW, Yu B, Mao XB, Zheng P, et al. Chlorogenic acid improves intestinal development via suppressing mucosa inflammation and cell apoptosis in weaned pigs. ACS Omega. 2018;3(2):2211–9.

23. Chen JL, Yu B, Chen DW, Zheng P, Luo YH, Huang ZQ, et al. Changes of porcine gut microbiota in response to dietary chlorogenic acid supplementation. Appl Microbiol Biotechnol. 2019;103:8157–68.

24. Chen JL, Yu B, Chen DW, Huang ZQ, Mao XB, Zheng P, et al. Chlorogenic acid improves intestinal barrier functions by suppressing mucosa inflammation and improving antioxidant capacity in weaned pigs. J Nutr Biochem. 2018;59:84–92.

25. Chen JL, Chen DW, Yu B, Luo YH, Zheng P, Mao XB, et al. Chlorogenic acid attenuates oxidative stress-induced intestinal mucosa disruption in weaned pigs. Front Vet Sci. 2022;9:806253.

26. Chen JL, Luo YH, Li Y, Chen DW, Yu B, He J. Chlorogenic acid attenuates oxidative stress-induced intestinal epithelium injury by co-regulating the PI3K/Akt and IκBα/NF-κB signaling. Antioxidants (Basel). 2021;10(12):1915.

27. Chen XL, Qiao TL, Mao ZY, Jia G, Zhao H, Liu GM, et al. Caffeic acid improves intestinal barrier functions by regulating colonic bacteria and tight junction protein expression and alleviating inflammation in weaning piglets. Anim Biotechnol. 2023;34(8):3693–99.

28. Wen XB, Wan F, Wu Y, Liu L, Liu YP, Zhong RQ, et al. Caffeic acid supplementation ameliorates intestinal injury by modulating intestinal microbiota in LPS-challenged piglets. Food Funct. 2023;14(16):7705–17.

29. Qin WX, Yu ZD, Li ZC, Liu HF, Li W, Zhao JN, et al. Dietary berberine and ellagic acid supplementation improve growth performance and intestinal damage by regulating the structural function of gut microbiota and scfas in weaned piglets. Microorganisms 2023;11(5):1254.

30. Lu YJ, Zhao MW, Mo JY, Lan GQ, Liang J. Dietary supplementation ellagic acid on the growth, intestinal immune response, microbiota, and inflammation in weaned piglets. Front Vet Sci. 2022;9:980271.

31. Qin WX, Xu BY, Chen YW, Yang WB, Xu YZ, Huang JC, et al. Dietary ellagic acid supplementation attenuates intestinal damage and oxidative stress by regulating gut microbiota in weanling piglets. Anim Nutr. 2022;11:322–333.

32. Xiao YX, Huang R, Wang N, Deng YK, Tan BE, Yin YL, et al. Ellagic acid alleviates oxidative stress by mediating Nrf2 signaling pathways and protects against paraquat-induced intestinal injury in piglets. Antioxidants (Basel). 2022;11(2):252.

33. Qi M, Wang N, Xiao YX, Deng YK, Zha AD, Tan BE, et al. Ellagic acid ameliorates paraquat-induced liver injury associated with improved gut microbial profile. Environ Pollut. 2022;293:118572.

34. Wang YX, Chen XL, Huang ZQ, Chen DW, Yu B, Chen H, et al. Effects of dietary ferulic acid supplementation on growth performance and skeletal muscle fiber type conversion in weaned piglets. J Sci Food Agric. 2021;101(12):5116–23.

35. Chen XL, Wang YX, Chen DW, Yu B, Huang ZQ. Dietary ferulic acid supplementation improves intestinal antioxidant capacity and intestinal barrier function in weaned piglets. Anim Biotechnol. 2022;33(2):356–61.

36. Wang YX, Chen XL, Huang ZQ, Chen DW, Yu B, Yu J, et al. Dietary ferulic acid supplementation improves antioxidant capacity and lipid metabolism in weaned piglets. Nutrients. 2020;12(12):3811.

37. Hu RZ, Wu SS, Li BZ, Tan JJ, Yan JH, Wang Y, et al. Dietary ferulic acid and vanillic acid on inflammation, gut barrier function and growth performance in lipopolysaccharide-challenged piglets. Anim Nutr. 2022;8:144–52.

38. Zhao XM, Wang JZ, Gao G, Bontempo V, Chen CQ, Schroyen M, et al. The influence of dietary gallic acid on growth performance and plasma antioxidant status of high and low weaning weight piglets. Animals (Basel). 2021;11(11):3323.

39. Cai L, Li YP, Wei ZX, Li XL, Jiang X. Effects of dietary gallic acid on growth performance, diarrhea incidence, intestinal morphology, plasma antioxidant indices, and immune response in weaned piglets. Anim Feed Sci Technol. 2020;261:114391.

40. Wu SS, Hu RZ, He ZY, Liu J, Liu M, Tan JJ, et al. Effect of protocatechuic acid on growth performance, inflammatory status and immune indices in weaned piglets. J Anim Sci. 2019;97(Supplement_3):361–2.

41. Hu RZ, He ZY, Liu M, Tan JJ, Zhang HF, Hou DX, et al. Dietary protocatechuic acid ameliorates inflammation and up-regulates intestinal tight junction proteins by modulating gut microbiota in LPS-challenged piglets. J Anim Sci Biotechnol. 2020;11:92.

42. Wang MW, Huang HJ, Wang L, Yin LM, Yang HS, Chen CQ, et al. Tannic acid attenuates intestinal oxidative damage by improving antioxidant capacity and intestinal barrier in weaned piglets and IPEC-J2 cells. Front Nutr. 2022;9:1012207.

43. Song YY, Luo Y, Yu B, He J, Zheng P, Mao XB, et al. Tannic acid extracted from gallnut prevents post-weaning diarrhea and improves intestinal health of weaned piglets. Anim Nutr. 2021;7(4):1078–86.

44. Yu J, Song YY, Yu B, He J, Zheng P, Mao XB, et al. Tannic acid prevents post-weaning diarrhea by improving intestinal barrier integrity and function in weaned piglets. J Anim Sci Biotechnol. 2020;11:87.

45. Xu TT, Ma X, Zhou XC, Qian MQ, Yang ZR, Cao PW, et al. Coated tannin supplementation improves growth performance, nutrients digestibility, and intestinal function in weaned piglets. J Anim Sci. 2022;100(5):skac088.

46. Liu HS, Hu JX, Mahfuz S, Piao XS. Effects of hydrolysable tannins as zinc oxide substitutes on antioxidant status, immune function, intestinal morphology, and digestive enzyme activities in weaned piglets. Animals (Basel). 2020;10(5):757.

47. Mei HD, Li YF, Tian QM, Li ZM, Rong T, Ma XY, et al. Effects of magnolol on nutrient apparent digestibility, serum biochemical indices, and their metabolite contents of weaned piglets. Chin J Anim Nutr. 2022;34(08):4919–31.

48. Zeng ZY, Chen XL, Huang Z, Chen DW, He J, Chen H, et al. Effects of dietary resveratrol supplementation on growth performance and muscle fiber type transformation in weaned piglets. Anim Feed Sci Technol. 2020;265:114499.

49. Chen XL, Zeng ZY, Huang ZQ, Chen DW, He J, Chen H, et al. Effects of dietary resveratrol supplementation on immunity, antioxidative capacity and intestinal barrier function in weaning piglets. Anim Biotechnol. 2021;32(2):240–5.

50. Zhao XH, Tong WZ, Song X, Jia RY, Li LX, Zou YF, et al. Antiviral effect of resveratrol in piglets infected with virulent pseudorabies virus. Viruses 2018;10(9):457.

51. Gao YN, Meng QW, Qin JW, Zhao QQ, Shi BM. Resveratrol alleviates oxidative stress induced by oxidized soybean oil and improves gut function via changing gut microbiota in weaned piglets. J Anim Sci Biotechnol. 2023;14:54.

52. Cheng K, Yu CY, Li ZH, Li SM, Yan EF, Song ZH, et al. Resveratrol improves meat quality, muscular antioxidant capacity, lipid metabolism and fiber type composition of intrauterine growth retarded pigs. Meat Sci. 2020;170:108237.

53. Cao ST, Shen ZJ, Wang CC, Zhang QH, Hong QH, He YH, et al. Resveratrol improves intestinal barrier function, alleviates mitochondrial dysfunction and induces mitophagy in diquat challenged piglets. Food Funct. 2019;10(1):344-54.

54. Xun WJ, Fu QY, Shi LG, Cao T, Jiang HZ, Ma ZH. Resveratrol protects intestinal integrity, alleviates intestinal inflammation and oxidative stress by modulating AhR/Nrf2 pathways in weaned piglets challenged with diquat. Int Immunopharmacol. 2021;99:107989.

55. Xun WJ, Fu QY, Hou GY, Shi LG, Cao T. Protective effects of dietary resveratrol supplementation against oxidative stress in diquat-challenged piglets. Ital J Anim Sci. 2020;19(1):1523–32.

56. Fu QY, Tan Z, Shi LG, Xun WJ. Resveratrol attenuates diquat-induced oxidative stress by regulating gut microbiota and metabolome characteristics in piglets. Front Microbiol. 2021;12:695155.

57. Hong QH, Li X, Lin Q, Shen ZJ, Feng J, Hu CH. Resveratrol improves intestinal morphology and anti-oxidation ability in deoxynivalenol-challenged piglets. Animals (Basel). 2022;12(3):311.

58. Qiu YQ, Yang J, Wang L, Yang XF, Gao KG, Zhu C, et al. Dietary resveratrol attenuation of intestinal inflammation and oxidative damage is linked to the alteration of gut microbiota and butyrate in piglets challenged with deoxynivalenol. J Anim Sci Biotechnol. 2021;12:71.

59. Qiu YQ, Nie XZ, Yang J, Wang L, Zhu C, Yang XF, et al. Effect of resveratrol supplementation on intestinal oxidative stress, immunity and gut microbiota in weaned piglets challenged with deoxynivalenol. Antioxidants (Basel). 2022;11(9):1775.

60. Cui QK, Fu QT, Zhao XH, Song X, Yu JK, Yang Y, et al. Protective effects and immunomodulation on piglets infected with rotavirus following resveratrol supplementation. PloS One. 2018;13(2):e0192692.

61. Fu QT, Cui QK, Yang Y, Zhao XH, Song X, Wang GX, et al. Effect of resveratrol dry suspension on immune function of piglets. Evid Based Complement Alternat Med. 2018;2018:5952707.

62. Zhang H, Chen YN, Li Y, Jia PL, Ji SL, Chen YP, et al. Protective effects of pterostilbene against hepatic damage, redox imbalance, mitochondrial dysfunction, and endoplasmic reticulum stress in weanling piglets. J Anim Sci. 2020;98(10):skaa328.

63. Li Y, Zhang H, Tu F, Cao J, Hou X, Chen YN, et al. Effects of resveratrol and its derivative pterostilbene on hepatic injury and immunological stress of weaned piglets challenged with lipopolysaccharide. J Anim Sci. 2022;100(12):skac339.

64. Zhang H, Chen YN, Chen YP, Jia PL, Ji SL, Xu JX, et al. Comparison of the effects of resveratrol and its derivative pterostilbene on hepatic oxidative stress and mitochondrial dysfunction in piglets challenged with diquat. Food Funct. 2020;11(5):4202–15.

65. Chen YN, Zhang H, Ji SL, Jia PL, Chen YP, Li Y, et al. Resveratrol and its derivative pterostilbene attenuate oxidative stress-induced intestinal injury by improving mitochondrial redox homeostasis and function via SIRT1 signaling. Free Radic Biol Med. 2021;177:1–14.

66. Zhang H, Chen YN, Chen YP, Ji SL, Jia PL, Li Y, et al. Comparison of the protective effects of resveratrol and pterostilbene against intestinal damage and redox imbalance in weanling piglets. J Anim Sci Biotechnol. 2020;11:52.

67. Chen YN, Zhang H, Chen YP, Jia PL, Ji SL, Zhang YY, et al. Resveratrol and its derivative pterostilbene ameliorate intestine injury in intrauterine growth-retarded weanling piglets by modulating redox status and gut microbiota. J Anim Sci Biotechnol. 2021;12:70.

68. Gan ZD, Wei WY, Wu JM, Zhao YW, Zhang LL, Wang T, et al. Resveratrol and curcumin improve intestinal mucosal integrity and decrease m6A RNA methylation in the intestine of weaning piglets. ACS Omega. 2019;4(17):17438–46.

69. Gan ZD, Wei WY, Li Y, Wu JM, Zhao YW, Zhang LL, et al. Curcumin and resveratrol regulate intestinal bacteria and alleviate intestinal inflammation in weaned piglets. Molecules. 2019;24(7):1220.

70. Niu Y, He JT, Zhao YW, Shen MM, Zhang LL, Zhong X, et al. Effect of curcumin on growth performance, inflammation, insulin level, and lipid metabolism in weaned piglets with IUGR. Animals (Basel). 2019;9(12):1098.

71. Niu Y, He JT, Ahmad H, Shen MM, Zhao YW, Gan ZD, et al. Dietary curcumin supplementation increases antioxidant capacity, upregulates Nrf2 and Hmox1 levels in the liver of piglet model with intrauterine growth retardation. Nutrients. 2019;11(12):2978.

72. Zhang LG, Zhang JQ, Yan EF, He JT, Zhong X, Zhang LL, et al. Dietary supplemented curcumin improves meat quality and antioxidant status of intrauterine growth retardation growing pigs via Nrf2 signal pathway. Animals (Basel). 2020;10(3):539.

73. Yan EF, Zhang JQ, Han HL, Wu JM, Gan ZD, Wei CH, et al. Curcumin alleviates IUGR jejunum damage by increasing antioxidant capacity through Nrf2/Keap1 pathway in growing pigs. Animals (Basel). 2019;10(1):41.

74. Wang K, Chen DW, Yu B, He J, Mao XB, Huang ZQ, et al. Eugenol alleviates transmissible gastroenteritis virus-induced intestinal epithelial injury by regulating NF-κB signaling pathway. Front Immunol. 2022;13:921613.

75. Wang TX, Yao WL, Li J, Shao YF, He QY, Xia J, et al. Dietary garcinol supplementation improves diarrhea and intestinal barrier function associated with its modulation of gut microbiota in weaned piglets. J Anim Sci Biotechnol. 2020;11:12.

76. Xu XJ, Chen XL, Huang ZQ, Chen DW, Yu B, Chen H, et al. Dietary apple polyphenols supplementation enhances antioxidant capacity and improves lipid metabolism in weaned piglets. J Anim Physiol Anim Nutr (Berl). 2019;103(5):1512–20.

77. Guo ZY, Chen XL, Huang ZQ, Chen DW, Yu J, Yan H, et al. Apple polyphenols improve intestinal barrier function by enhancing antioxidant capacity and suppressing inflammation in weaning piglets. Anim Sci J. 2022;93(1):e13747.

78. Hara H, Orita N, Hatano S, Ichikawa H, Hara Y, Matsumoto N, et al. Effect of tea polyphenols on fecal flora and fecal metabolic products of pigs. J Vet Med Sci. 1995;57(1):45–9.

79. Deng QL, Xu J, Yu B, He J, Zhang KY, Ding XM, et al. Effect of dietary tea polyphenols on growth performance and cell-mediated immune response of post-weaning piglets under oxidative stress. Arch Anim Nutr. 2010;64(1):12–21.

80. Huang FY, Yang Y, Wang LM, Wang H, Li P, Xiao K, et al. Holly polyphenols attenuate liver injury, suppression inflammation and oxidative stress in lipopolysaccharide-challenged weaned pigs. Food Agric Immunol. 2022;33(1):35–46.

81. Xu X, Hua HW, Wang LM, He PW, Zhang L, Qin Q, et al. Holly polyphenols alleviate intestinal inflammation and alter microbiota composition in lipopolysaccharide-challenged pigs. Br J Nutr. 2020;123(8):881–91.

82. Xu X, Wei Y, Hua HW, Jing XQ, Zhu HL, Xiao K, et al. Polyphenols sourced from *Ilex Latifolia* Thunb. relieve intestinal injury via modulating ferroptosis in weanling piglets under oxidative stress. Antioxidants (Basel). 2022;11(5):966.

83. He PW, Hua HW, Tian W, Zhu HL, Liu YL, Xu X. Holly (*Ilex latifolia* Thunb.) polyphenols extracts alleviate hepatic damage by regulating ferroptosis following diquat challenge in a piglet model. Front Nutr. 2020;7:604328.

84. Leskovec J, Rezar V, Nemec Svete A, Salobir J, Levart A. Antioxidative effects of olive polyphenols compared to vitamin E in piglets fed a diet rich in n-3 PUFA. Animals (Basel). 2019;9(4):161.

85. Fiesel A, Gessner DK, Most E, Eder K. Effects of dietary polyphenol-rich plant products from grape or hop on pro-inflammatory gene expression in the intestine, nutrient digestibility and faecal microbiota of weaned pigs. BMC Vet Res. 2014;10:196.

86. Gessner DK, Fiesel A, Most E, Dinges J, Wen G, Ringseis R, et al. Supplementation of a grape seed and grape marc meal extract decreases activities of the oxidative stress-responsive transcription factors NF-κB and Nrf2 in the duodenal mucosa of pigs. Acta Vet Scand. 2013;55:18.

87. Papakonstantinou GI, Meletis E, Petrotos K, Kostoulas P, Tsekouras N, Kantere MC, et al. Effects of a natural polyphenolic product from olive mill wastewater on oxidative stress and post-weaning diarrhea in piglets. Agriculture. 2023;13(7):1356.

88. Verhelst R, Schroyen M, Buys N, Niewold T. Dietary polyphenols reduce diarrhea in enterotoxigenic *Escherichia coli* (ETEC) infected post-weaning piglets. Livest Sci. 2014;160:138–40.

89. Silva Júnior CD, Martins CC, Dias FT, Sitanaka NY, Ferracioli LB, Moraes JE, et al. The use of an alternative feed additive, containing benzoic acid, thymol, eugenol, and piperine, improved growth performance, nutrient and energy digestibility, and gut health in weaned piglets. J Anim Sci. 2020;98(5):skaa119.

90. Zhang YY, Li Q, Wang ZX, Dong Y, Yi D, Wu T, et al. Dietary supplementation with a complex of cinnamaldehyde, carvacrol, and thymol negatively affects the intestinal function in LPS-challenged piglets. Front Vet Sci: 2023;10:1098579.

91. Hossain MM, Hwang HS, Jang SY, Yu S, Kim IH. Supplemental impact of silymarin in growing pig diet on the growth performance, total tract digestibility, faecal microflora, faecal noxious gas emission and absorption rate in blood. J Anim Physiol Anim Nutr (Berl). 2024;108(1):206–14.

92. Park JH, Sureshkumar S, Kim IH. Influences of dietary flavonoid (quercetin) supplementation on growth performance and immune response of growing pigs challenged with Escherichia coli lipopolysaccharide. J Anim Sci Technol. 2020;62(5):605.

93. Liu YC, Li YF, Yu M, Tian ZM, Deng JJ, Ma XY, et al. Magnolol supplementation alters serum parameters, immune homeostasis, amino acid profiles, and gene expression of amino acid transporters in growing pigs. Int J Mol Sci. 2023;24(18):13952.

94. Li YF, Liu YC, Mu CL, Zhang CY, Yu M, Tian ZM, et al. Magnolol-driven microbiota modulation elicits changes in tryptophan metabolism resulting in reduced skatole formation in pigs. J Hazard Mater. 2024;467:133423.

95. Yan L, Kim I. Effect of eugenol and cinnamaldehyde on the growth performance, nutrient digestibility, blood characteristics, fecal microbial shedding and fecal noxious gas content in growing pigs. Asian-Australas J Anim Sci. 2012;25(8):1178.

96. Sun ZW, Li DF, Li Y, Chen DW, Yu B, Yu J, et al. Effects of dietary daidzein supplementation on growth performance, carcass characteristics, and meat quality in growing-finishing pigs. Anim Feed Sci Technol. 2020;268:114591.

97. Wei C, Chen XL, Chen DW, He J, Zheng P, Chen H, et al. Effects of dietary dihydromyricetin supplementation on intestinal barrier and humoral immunity in growing-finishing pigs. Anim Biotechnol. 2022;33(7):1398–406.

98. Guo ZY, Chen XL, Huang ZQ, Chen DW, Li MZ, Yu B, et al. Dihydromyricetin improves meat quality and promotes skeletal muscle fiber type transformations via AMPK signaling in growing–finishing pigs. Food Funct. 2022;13(6):3649–59.

99. Guo ZY, Chen XL, Huang ZQ, Chen DW, Yu B, Chen H, et al. Dietary dihydromyricetin supplementation enhances antioxidant capacity and improves lipid metabolism in finishing pigs. Food Funct. 2021;12(15):6925–35.

100. Wei C, Chen XL, Chen DW, Yu B, Zheng P, He J, et al. Dihydromyricetin enhances intestinal antioxidant capacity of growing-finishing pigs by activating ERK/Nrf2/HO-1 signaling pathway. Antioxidants (Basel). 2022;11(4):704.

101. Xie KH, Sun YX, Deng LL, Yu B, Luo YH, Huang ZQ, et al. Effects of dietary chlorogenic acid supplementation on growth performance, meat quality, and muscle flavor substances in finishing pigs. Foods. 2023;12(16):3047.

102. Li HW, Chen XL, Chen DW, Yu B, He J, Zheng P, et al. Ellagic acid alters muscle fiber-type composition and promotes mitochondrial biogenesis through the AMPK signaling pathway in healthy pigs. J Agric Food Chem. 2022;70(31):9779–89.

103. Zhang HZ, Chen DW, He J, Zheng P, Yu J, Mao XB, et al. Long-term dietary resveratrol supplementation decreased serum lipids levels, improved intramuscular fat content, and changed the expression of several lipid metabolism-related miRNAs and genes in growing-finishing pigs. J Anim Sci. 2019;97(4):1745–56.

104. Wang Q, Wang J, Qi RL, Qiu X, Sun QY, Huang JX. Naringin supplementation affects performance, carcass traits, meat quality and oxidative stability of finishing pigs. S Afr J Anim Sci. 2020;50(1):78–87.

105. Zhang QQ, Kim IH. Micelle silymarin supplementation to fattening diet augments daily gain, nutrient digestibility, decreases toxic gas emissions, and ameliorates meat quality of fattening pigs. Czech J Anim Sci. 2022;67(4):125–36.

106. Liu YY, Xiao Y, Xie JC, Peng YL, Li F, Chen C, et al. Dietary supplementation with flavonoids from mulberry leaves improves growth performance and meat quality, and alters lipid metabolism of skeletal muscle in a chinese hybrid pig. Anim Feed Sci Technol. 2022;285:115211.

107. Liu YY, Peng YL, Chen C, Ren HB, Zhu J, Deng Y, et al. Flavonoids from mulberry leaves inhibit fat production and improve fatty acid distribution in adipose tissue in finishing pigs. Anim Nutr. 2023;16:147–57.

108. Xu M, Chen XL, Huang ZQ, Chen DW, Li MZ, He J, et al. Effects of dietary grape seed proanthocyanidin extract supplementation on meat quality, muscle fiber characteristics and antioxidant capacity of finishing pigs. Food Chem. 2022;367:130781.

109. Feng YD, Chen XL, Chen DW, He J, Zheng P, Luo YH, et al. Dietary grape seed proanthocyanidin extract supplementation improves antioxidant capacity and lipid metabolism in finishing pigs. Anim Biotechnol. 2023;34(8):4021–31.

110. Wang WL, Li FN, Duan YH, Guo QP, Zhang LY, Yang YH, et al. Effects of dietary chlorogenic acid supplementation derived from *lonicera macranthoides* hand-mazz on growth performance, free amino acid profile, and muscle protein synthesis in a finishing pig model. Oxid Med Cell Longev. 2022;2022:6316611.

111. Wang WL, Wen CY, Guo QP, Li JZ, He SP, Yin YL. Dietary supplementation with chlorogenic acid derived from *Lonicera macranthoides* Hand-Mazz improves meat quality and muscle fiber characteristics of finishing pigs via enhancement of antioxidant capacity. Front Physiol. 2021;12:650084.

112. Wu Y, Liu WH, Li Q, Li YF, Yan YL, Huang F, et al. Dietary chlorogenic acid regulates gut microbiota, serum-free amino acids and colonic serotonin levels in growing pigs. Int J Food Sci Nutr. 2018;69(5):566–73.

113. Valenzuela-Grijalva N, Jiménez-Estrada I, Mariscal-Tovar S, López-García K, Pinelli-Saavedra A, Peña-Ramos EA, et al. Effects of ferulic acid supplementation on growth performance, carcass traits and histochemical characteristics of muscle fibers in finishing pigs. Animals (Basel). 2021;11(8):2455.

114. Zhang QJ, Gong JT, Xiang HK, Hu RZ, Yang XZ, Lv J, et al. Effects of main active components of rosemary on growth performance, meat quality and lipid metabolism in finishing pigs. Anim Nutr. 2023;15:341–9.

115. Huang YN, Xia Q, Cui YY, Qu QH, Wei YM, Jiang QY. Resveratrol increase the proportion of oxidative muscle fiber through the AdipoR1-AMPK-PGC-1α pathway in pigs. J Funct Foods. 2020;73:104090.

116. Luo P, Luo L, Zhao WJ, Wang LS, Sun LJ, Wu HY, et al. Dietary thymol supplementation promotes skeletal muscle fibre type switch in longissimus dorsi of finishing pigs. J Anim Physiol Anim Nutr (Berl). 2020;104(2):570–8.

117. Xu XJ, Chen XL, Huang ZQ, Chen DW, He J, Zheng P, et al. Effects of dietary apple polyphenols supplementation on hepatic fat deposition and antioxidant capacity in finishing pigs. Animals (Basel). 2019;9(11):937.

118. Xu XJ, Chen XL, Chen DW, Yu B, Yin JD, Huang ZQ. Effects of dietary apple polyphenol supplementation on carcass traits, meat quality, muscle amino acid and fatty acid composition in finishing pigs. Food Funct. 2019;10(11):7426–34.

119. Chen XL, Jia G, Liu GM, Zhao H, Huang ZQ. Effects of apple polyphenols on myofiber-type transformation in longissimus dorsi muscle of finishing pigs. Anim Biotechnol. 2021;32(2):246–53.

120. Huang TT, Che QJ, Chen XL, Chen DW, Yu B, He J, et al. Apple polyphenols improve intestinal antioxidant capacity and barrier function by activating the Nrf2/Keap1 signaling pathway in a pig model. J Agric Food Chem. 2022;70(24):7576–85.

121. Varricchio E, Coccia E, Orso G, Lombardi V, Imperatore R, Vito P, et al. Influence of polyphenols from olive mill wastewater on the gastrointestinal tract, alveolar macrophages and blood leukocytes of pigs. Ital J Anim Sci. 2019;18(1):574–86.

122. Zheng SG, Song JC, Qin X, Yang K, Liu M, Yang CB, et al. Dietary supplementation of red-osier dogwood polyphenol extract changes the ileal microbiota structure and increases *Lactobacillus* in a pig model. AMB Express. 2021;11(1):1–12.

123. Xie KH, Li Y, Chen DW, Yu B, Luo YH, Mao XB, et al. Daidzein supplementation enhances embryo survival by improving hormones, antioxidant capacity, and metabolic profiles of amniotic fluid in sows. Food Funct. 2020;11(12):10588–600.

124. Xie KH, Li Y, He GR, Zhao XF, Chen DW, Yu B, et al. Daidzein supplementation improved fecundity in sows via modulation of ovarian oxidative stress and inflammation. J Nutr Biochem. 2022;110:109145.

125. Li Y, He GR, Chen DW, Yu B, Yu J, Zheng P, et al. Supplementing daidzein in diets improves the reproductive performance, endocrine hormones and antioxidant capacity of multiparous sows. Anim Nutr. 2021;7(4):1052–60.

126. Li DS, Dang DX, Xu SY, Tian YM, Wu D, Su Y. Soy isoflavones supplementation improves reproductive performance and serum antioxidant status of sows and the growth performance of their offspring. J Anim Physiol Anim Nutr (Berl). 2022;106(6):1268–76.

127. Wu HZ, Yang J, Wang SB, Zhang X, Hou JW, Xu F, et al. Effects of soybean isoflavone and astragalus polysaccharide mixture on colostrum components, serum antioxidant, immune and hormone levels of lactating sows. Animals (Basel). 2021;11(1):132.

128. Wei LK, Hou GF, Long CM, Chen FM, Bai XL, Li R, et al. Dietary silymarin ameliorating reproductive and lactation performance of sows via regulating body antioxidant and metabolism. Digital Chin Med 2022;5(3):286–94.

129. Jiang XJ, Lin S, Lin Y, Fang ZF, Xu SY, Feng B, et al. Effects of silymarin supplementation during transition and lactation on reproductive performance, milk composition and haematological parameters in sows. J Anim Physiol Anim Nutr (Berl). 2020;104(6):1896–903.

130. Zhang QQ, Ahn JM, Kim IH. Micelle silymarin supplementation to sows’ diet from day 109 of gestation to entire lactation period enhances reproductive performance and affects serum hormones and metabolites. J Anim Sci. 2021;99(12):skab354.

131. Meng QW, Sun SS, Bai YS, Luo Z, Li ZY, Shi BM, et al. Effects of dietary resveratrol supplementation in sows on antioxidative status, myofiber characteristic and meat quality of offspring. Meat Sci. 2020;167:108176.

132. Zhao Y, Huang YJ, Gao KG, Wen XL, Hu SL, Wang L, et al. Maternal resveratrol regulates the growth performance, antioxidant capacity, and intestinal health of suckling piglets through intestinal microorganisms at high summer temperatures. Front Nutr. 2022;9:971496.

133. Meng QW, Sun SS, Luo Z, Shi BM, Shan AS, Cheng BJ. Maternal dietary resveratrol alleviates weaning-associated diarrhea and intestinal inflammation in pig offspring by changing intestinal gene expression and microbiota. Food Funct. 2019;10(9):5626–43.

134. Sun SS, Meng QW, Luo Z, Shi BM, Bi CP, Shan AS. Effects of dietary resveratrol supplementation during gestation and lactation of sows on milk composition of sows and fat metabolism of sucking piglets. J Anim Physiol Anim Nutr (Berl). 2019;103(3):813–21.

135. Meng QW, Guo T, Li GQ, Sun SS, He SQ, Cheng BJ, et al. Dietary resveratrol improves antioxidant status of sows and piglets and regulates antioxidant gene expression in placenta by Keap1-Nrf2 pathway and Sirt1. J Anim Sci Biotechnol. 2018;9:34.

136. Heras-Molina A, Pesantez-Pacheco JL, Astiz S, Garcia-Contreras C, Vazquez-Gomez M, Encinas T, et al. Maternal supplementation with polyphenols and omega-3 fatty acids during pregnancy: Effects on growth, metabolism, and body composition of the offspring. Animals (Basel). 2020;10(11):1946.

137. Heras-Molina A, Escudero R, Pesántez-Pacheco JL, García-Contreras C, Vázquez-Gómez M, Astiz S, et al. Maternal supplementation with polyphenols and omega-3 fatty acids during pregnancy: Prenatal effects on fetal fatty acid composition in the Iberian pig. Animals (Basel). 2022;12(16):2140.

138. Garcia-Contreras C, Vazquez-Gomez M, Barbero A, Pesantez JL, Zinellu A, Berlinguer F, et al. Polyphenols and IUGR pregnancies: Effects of maternal hydroxytyrosol supplementation on placental gene expression and fetal antioxidant status, DNA-methylation and phenotype. Int J Mol Sci. 2019;20(5):1187.

139. Garcia-Contreras C, Vazquez-Gomez M, Pardo Z, Heras-Molina A, Pesantez JL, Encinas T, et al. Polyphenols and IUGR pregnancies: Effects of maternal hydroxytyrosol supplementation on hepatic fat accretion and energy and fatty acids profile of fetal tissues. Nutrients. 2019;11(7):1534.

140. Wang TX, Yao WL, Xia J, Li J, Shao YF, Huang FR. Dietary supplementation with garcinol during late gestation and lactation facilitates acid–base balance and improves the performance of sows and newborn piglets. J Anim Sci. 2019;97(11):4557–66.

141. Wang XR, Jiang GT, Kebreab E, Yu QF, Li JH, Zhang X, et al. Effects of dietary grape seed polyphenols supplementation during late gestation and lactation on antioxidant status in serum and immunoglobulin content in colostrum of multiparous sows. J Anim Sci. 2019;97(6):2515–23.

142. Fang CK, Tang XP, Zhang QT, Yu QF, Deng ST, Wu SS, et al. Effects of dietary *Lonicera flos* and *Sucutellaria baicalensis* mixed extracts supplementation on reproductive performance, umbilical cord blood parameters, colostrum ingredients and immunoglobulin contents of late-pregnant sows. Animals (Basel). 2024;14(14):2054.
